# Total Synthesis and Anti-Inflammatory Evaluation of Osajin, Scandenone and Analogues

**DOI:** 10.3390/ph17010086

**Published:** 2024-01-09

**Authors:** Rui Wang, Ran Ma, Ke Feng, Hongchen Lu, Wei Zhao, Hongzhen Jin

**Affiliations:** 1 State Key Laboratory of Medicinal Chemical Biology, Key Laboratory of Molecular Drug Research and KLMDASR of Tianjin, College of Pharmacy, Nankai University, Tianjin 300350, China; 1120200572@mail.nankai.edu.cn (R.W.); 2120221619@mail.nankai.edu.cn (R.M.); 2120211302@mail.nankai.edu.cn (K.F.); 2120231693@mail.nankai.edu.cn (H.L.); 2 Tianjin International Joint Academy of Biomedicine, Tianjin 300457, China; 3 School of Health and Life Sciences, University of Health and Rehabilitation Sciences, Qingdao 266071, China

**Keywords:** flavonoids, natural products, anti-inflammatory, Suzuki coupling reaction

## Abstract

In this study, the total synthesis of osajin, scandenone and their analogues have been accomplished. The key synthetic steps include aldol/intramolecular iodoetherification/elimination sequence reactions and a Suzuki coupling reaction to assemble the tricyclic core, chemoselective propargylation and Claisen rearrangement reactions to obtain natural compounds. In addition, we also designed and synthesized twenty-five natural product analogues. All synthetic compounds were screened for anti-inflammatory activity against tumor necrosis factor-α (TNF-α) and interleukin-6 (IL-6) in lipopolysaccharide (LPS)-stimulated RAW264.7 macrophages. Collectively, Compound **39e** and **39d** were considered as promising lead compounds for further development.

## 1. Introduction

Flavonoids are naturally occurring secondary metabolites predominantly originating from fruits, herbs, fungi and vegetables which are characterized by a 2-phenyl-4*H*-chromene structure [1,2]. Flavonoids, a class of natural polyphenolics, are classified into flavanols, flavanones, flavones, isoflavones, flavonols and anthocyanidins [1]. Modern pharmacological evaluations and animal studies have demonstrated their anticancer, anti-inflammatory, antioxidant, antimicrobial and antiviral activities [3,4,5,6]. In the last decades, various synthetic and natural flavonoid derivatives have been actively investigated as drugs used in treating human disease [7,8]. Despite the fact that a large number of flavonoids have been isolated and identified, there is still need for further exploitation of bioactive lead compounds with novel structures.

**Figure 1:**
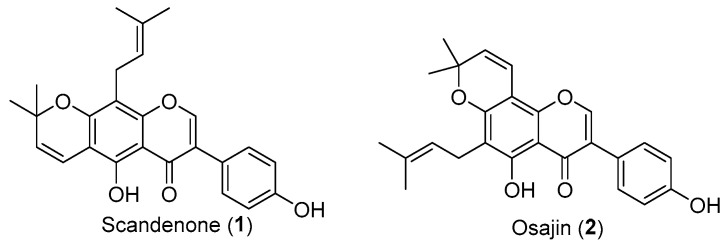
The structure of scandenone (**1**) and osajin (**2**).

Osajin and scandenone are natural flavonoid compounds that exist in various plants and Chinese herbal medicines such as *Derris scandens*, *Flemingia philippinensis* and *Millettia pulchra* (Figure 1) [9,10,11]. These compounds were elucidated based on detailed analysis of NMR and HRMS data [12]. Previous studies have shown that these natural products impede the growth of various cancerous cells and possess anti-inflammatory activities [13,14]. Due to their interesting biological activities, further research and development are necessary for the discovery of lead compounds. Herein, the total synthesis of osajin and scandenone is described. In addition, we synthesized and characterized twenty-five natural product analogues through different synthetic routes. All the newly synthesized compounds were tested for their anti-inflammatory activities against tumor necrosis factor-α (TNF-α) and interleukin-6 (IL-6) in lipopolysaccharide (LPS)-stimulated RAW264.7 macrophages.

## 2. Results and Discussion

**Scheme 1:**
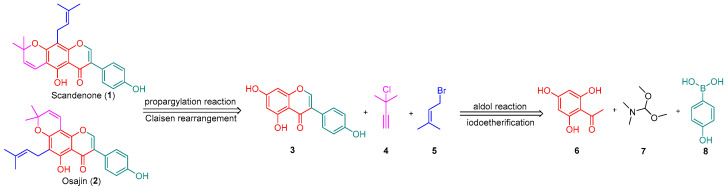
Retrosynthetic analysis of scandenone (**1**) and osajin (**2**), different colours represent different structure.

The isolated compounds are characterized by a C_6_-C_3_-C_6_ skeleton structure with a 2″,2″-dimethylpyran ring and a linear side chain. The retrosynthetic analysis of target molecules is depicted in Scheme 1. Osajin and scandenone were envisaged to be obtained via chemoselective propargylation, intramolecular cyclization and Claisen rearrangement sequence reactions of compound **3** [15]. The key intermediate **3** could be derived from the materials 1-(2,4,6-trihydroxyphenyl)ethan-1-one (**6**), 1,1-dimethoxy-*N*,*N*-dimethylmethanamine (**7**) and (4-hydroxyphenyl)boronic acid (**8**) through aldol/intramolecular iodoetherification/elimination/Suzuki coupling sequential chemical reactions (Scheme 1) [16].

**Scheme 2:**
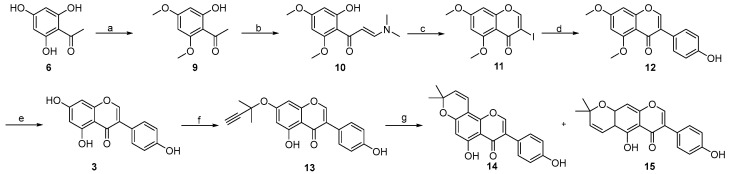
Synthesis of the intermediates **14** and **15**. Regents and conditions: (**a**) dimethyl sulfate, K_2_CO_3_, acetone, 60 °C, 4 h, 90%; (**b**) DMF-DMA, DMF, 80 °C, 1 h, 88%; (**c**) iodine, pyridine, MeOH, rt, 3 h, 78%; (**d**) (4-hydroxyphenyl)boronic acid, PdCl_2_(dppf), K_2_CO_3_, 1,4-Dioxane/H_2_O, 50 °C, 2 h, 86%; (**e**) 40% HBr (aq), 140 °C, 4 h, 78%; (**f**) 3-chloro-3-methylbut-1-yne, CuI, K_2_CO_3_, KI, DMF, rt, 5 h, 72% and (**g**) diethylaniline, 250 °C, 1 h, **14** (52%), **15** (41%).

The key intermediate **10** was prepared via coupling of compounds **6** and **7** based on the aldol reaction, which may suffer from poor functional group tolerance to exposed hydroxyl groups. For this reason, the free hydroxyl group of substance **6** was protected using a methyl group to give **9** with satisfactory yield, and the methyl-protected compound **9** was reacted with **7** to afford **10** in 88% yield. Compound **10** was further reacted to obtain **11** via addition reaction and we detected the low yield (from 20% to 36%) by trying different solvents and temperatures at the beginning. We introduced one equivalent pyridine into the reaction system, which improved the yield to 78%. The coupling of compounds **11** and **8** resulted in compound **12** via the Suzuki coupling reaction, which was deeply explored under different reaction systems. We were only afforded compound **12** with a 40% yield under the conditions of Pd(OAc)_2_/MeOH/Na_2_CO_3_ (Table 1, entry 1) [17]. The yield of the reaction has not been significantly improved by changing the solvent and base (entries 2 and 3). The yield was increased up to 60% when the temperature of the reaction system reached 50 °C (entry 4). Following this, we selected Pd(PPh_3_)_4_ as catalyst for further investigation [18]. After the systematic research of the reaction conditions, we found that increasing the temperature of this reaction system obviously improved the yield of **12** (entries 5–7). When the temperature was further increased, the yield was not remarkably improved (entries 7 and 8). The optimal condition was obtained by using PdCl_2_(dppf) as a catalyst and 1,4-Dioxane/H_2_O as a solvent at 50 °C (entry 9) [19]. The coupled product **12** proceeded smoothly in the presence of a 40% aqueous solution of HBr in refluxing water to provide the tricyclic core **3**. Chemoselective propargylation of the C7-hydroxyl of compound **3** provided **13** under the conditions of KI/K_2_CO_3_/CuI/ in 72% yield, which suffered an aromatic Claisen rearrangement, resulting in cyclization compound **14** (52% yield) and **15** (41% yield) under an elevated temperature condition of 250 °C (Scheme 2) [20].

**Scheme 3:**
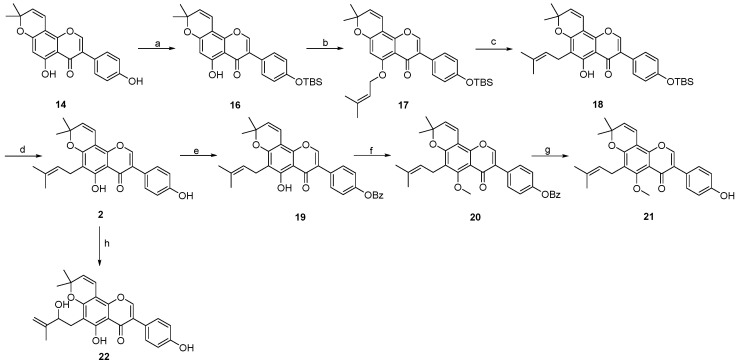
Synthesis of osajin (**2**) and compounds **19**–**22**. Regents and conditions: (**a**) tert-butyldimethylsilyl chloride, imidazole, DCM, rt, 30 h, 93%; (**b**) 3,3-dimethylallyl bromide, NaH, DMF, rt, 1 h, 69%; (**c**) montmorillonite K10, DCM, rt, 1 h, 65%; (**d**) TBAF, THF, rt, 0.5 h, 90%; (**e**) benzoyl chloride, DMAP, trimethylamine, DCM, rt, 2 h, 90%; (**f**) AgO, CH_3_I, chloroform, 80 °C, 6 h, 79%; (**g**) NaHCO_3_, MeOH, rt, 2 h, 87% and (**h**) (i). Rose bengal, MeOH, hv, O_2_, rt, 10 h; (ii). PPh_3_, rt, 1 h, 56%.

**Scheme 4:**
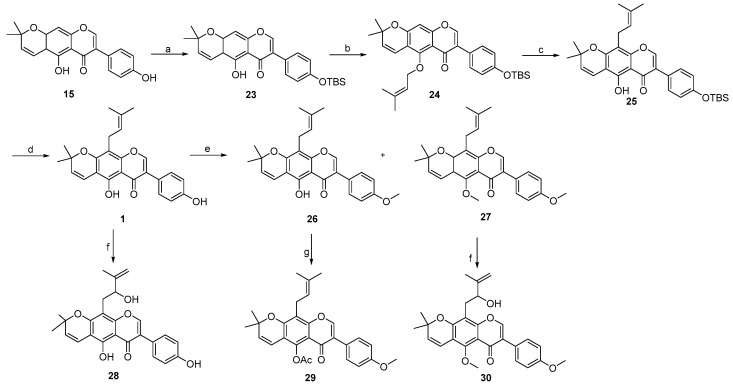
Synthesis of scandenone (**1**) and compounds **26**–**30**. Regents and conditions: (**a**) TBSCl, imidazole, DCM, rt, 25 h, 95%; (**b**) 3,3-dimethylallyl bromide, NaH, DMF, rt, 1 h, 74%; (**c**) Eu(fod)_3_, chloroform, 60 °C, 8 h, 76%; (**d**) TBAF, THF, rt, 0.5 h, 93%; (**e**) dimethyl sulfate, K_2_CO_3_, acetone, 60 °C, 4 h, **26** (46%), **27** (44%); (**f**) (i). Rose bengal, MeOH, hv, O_2_, rt, 10 h; (ii). PPh_3_, rt, 1 h, **28** (65%), **30** (57%) and (**g**) acetic anhydride, TEA, DMAP, rt, 4 h, 96%.

Having prepared the critical intermediate **14** successfully, we sought to establish skeletons of natural products. The C4′-hydroxy group of intermediate **14** was protected using a tert-butyldimethylsilyl (TBS) group to give **16**, which was further reacted to obtain **17** via a nucleophilic substitution reaction (64% yield in two steps). Compound **17** was subjected to Claisen rearrangement reaction to obtain **18** in 65% yield (along with 25% isopentenyl exfoliation product **16** as a byproduct). Deprotection of the TBS group of **18** was smoothly accomplished upon treatment with a large excess of tetrabutylammonium fluoride (TBAF) in THF to afford **2** in 90% yield at room temperature. Based on the above, we successfully synthesized the natural product osajin (**2**) (5.82% overall yield). NMR spectroscopic data of the synthetic osajin (**2**) were well matched with those reported in the literature (Supplementary Table S2) [12]. Flavonoids containing the hydroxyisoprenyl structure usually exhibit multiple biological activities. The Schenck ene reaction of the natural product **2** with the photosensitizer yielded secondary allylic alcohol **22**. Beyond that, we synthesized natural product analogues **19**–**21** through etherification, esterification and hydrolysis reactions (Scheme 3). The TBS-protected compound **23** was prenylated with 3,3-dimethylallyl bromide **5** to afford **24** (74% yield), which was further reacted in the presence of Eu(fod)_3_ to obtain the desired rearrangement product **25** (76% yield). Subsequent deprotection of the 4′-OH of **25** with TBAF afforded the natural products **1** in 93% yield (7.07% overall yield). NMR spectroscopic data of the synthetic scandenone (**1**) were well matched with those reported in the literature (Supplementary Table S1) [12]. In previous synthesis, the conversion of compound **3** to **41** is accompanied by the production of byproduct **41** and **42** (Supplementary Scheme S1), which limited the yield of this reaction [21]. We synthesized natural compounds through a series of reactions and the new synthesis methods provide approaches for the structural modification of flavonoids. In the same way, we synthesized a new series of analogues **26**–**30** via acetylation or methylation of the free hydroxy groups and the Schenck ene reaction (Scheme 4) [22].

**Scheme 5:**
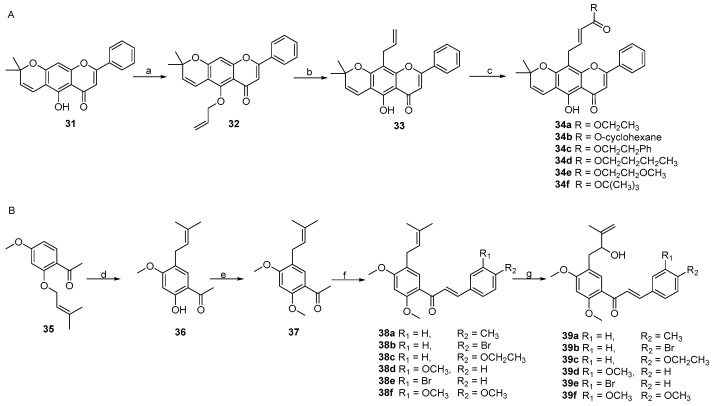
(**A**) Synthesis of compounds **34a**–**34g**. (**B**) Synthesis of compounds **39a**–**39f**. Regents and conditions: (**a**) allyl bromide, NaH, DMF, rt, 3 h, 76%; (**b**) diethylaniline, 230 °C, 0.5 h, 85%; (**c**) Grubbs catalyst 2^nd^ generation, DCM, rt, 5 h, yield ranging from 85% to 92%; (**d**) diethylaniline, 230 °C, 1 h, 89%; (**e**) CH_3_I, K_2_CO_3_, DMF, rt, 2 h, 93%; (**f**) aromatic aldehydes, NaOH, EtOH, 50 °C, 24 h, yield ranging from 70% to 91% and (**g**) (i). Rose bengal, MeOH, hv, O_2_, rt, 10 h and (ii). PPh_3_, rt, 1 h, yield ranging from 50% to 62%.

In previous work, we explored the new synthetic routes for the structural modification of flavonoids. Herein, we synthesized and characterized a series of natural product analogues and tested their anti-inflammatory activity. Compound **31** was converted to **33** in two steps via a nucleophilic substitution reaction with allyl bromide followed by Claisen rearrangement at a high temperature (65% yield in two steps). Compound **33** was treated with available reagents under alkene metathesis reaction conditions, leading to **34a**–**34g** (yield ranging from 85% to 92%) (Scheme 5A). Compound **35** was subjected to Claisen rearrangement/methylation/aldol sequence reactions to establish the chalcone skeleton **38a**–**38f** [23]. We introduced different substituents on the benzene ring of compound **38** to increase the structural diversity. Subsequently, we synthesized a range of analogues **39a**–**3** that possess a hydroxyisoprenyl structure based on the Schenck ene reaction (overall yield ranging from 29% to 47%) (Scheme 5B) [22].

**Figure 2:**
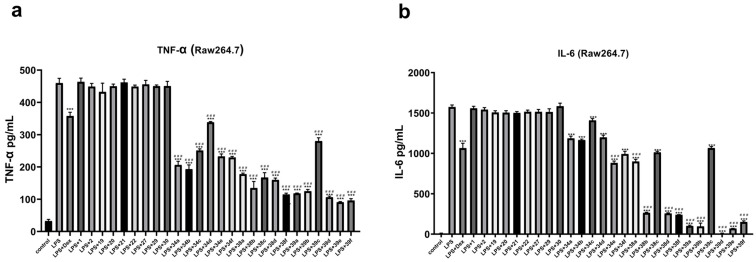
(**a**) The synthetic compounds suppress the production of TNF-α. (**b**) The synthetic compounds suppress the production of IL-6. Effects of analogues on the production of serum TNF-α and IL-6 induced via LPS. The results are shown as means ± SD (*n* = 3) of at least three independent experiments. The results are shown as *** *p* < 0.001 versus LPS and ^###^ *p* < 0.001 versus LPS + Dex.

**Figure 3:**
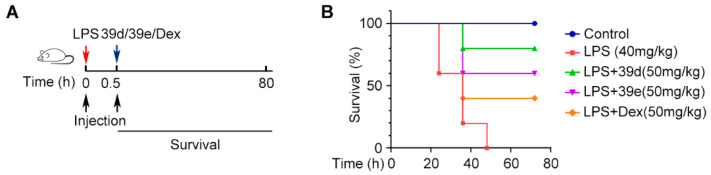
(**A**) Schematic of the mouse sepsis model and administration methods. (**B**) The effect of **39d**/**39e** on the survival rate of septic mice (*n* = 5).

We attempted to evaluate the anti-inflammatory effects of the synthetic compounds by measuring the levels of TNF-α and IL-6 in LPS-stimulated RAW264.7 macrophages with dexamethasone as a reference control [24]. Most of the compounds suppressed various degrees of cytokine liberation contrasted with the LPS control. The activity of the compounds was improved by introducing different side chains (Scheme 5A, **34a**–**34f**). Subsequently, we constructed the structure of chalcone and removed the 2″,2″-dimethylpyran ring. Compounds **38a**–**38f** yielded secondary allylic alcohol **39a**–**39f** with the photosensitizer via the Schenck ene reaction. Compounds containing a chalcone skeleton and the hydroxyisoprenyl side chain exhibited remarkable anti-inflammatory activity, and compounds **39e** and **39d** exhibited the highest inhibitory activity among them, respectively (Figure 2a,b). Meanwhile, we verified the excellent anti-inflammatory activity in vivo with a biological evaluation of mice. Compounds **39d** and **39e** presented much stronger inhibitory effects against the LPS-induced inflammatory response compared to the reference control dexamethasone (Figure 3). These compounds were considered as promising lead compounds for further development to discover new therapeutic agents with anti-inflammatory properties.

## 3. Materials and Methods

### 3.1. General Information

All reactions were carried out under an inert nitrogen atmosphere with dry solvents under anhydrous conditions unless otherwise stated. Flash chromatography was performed using silica gel (200–400 mesh). Thin layer chromatography (TLC) was performed using Silica gel 60 F254 plates and visualized using UV light.

^1^H and ^13^C spectra were recorded with Bruker Avance II 400 [400 MHz] and calibrated using the residual solvent as an internal reference [ ^1^H NMR: CDCl_3_ (7.26); ^13^C NMR: CDCl_3_ (77.16); DMSO-d_6_ = 2.50; DMSO-d_6_ = 39.52]. Signals were described as s = singlet, d = doublet, t = triplet, q = quartet and m = multiplet. High resolution mass spectra (HRMS) were recorded on an IonSpec QFT mass spectrometer with ESI ionization.

### 3.2. Materials

All commercially available chemicals and solvents were used as received without further purification unless otherwise stated.

### 3.3. Procedure for the Synthesis of Osajin and Scandenone

To a stirred solution of 1-(2,4,6-trihydroxyphenyl)ethan-1-one (5.00 g, 29.73 mmol) and K_2_CO_3_ (9.04 g, 65.44 mmol) in anhydrous acetone (80 mL), dimethyl sulfate (5.78 mL, 60.95 mmol) was slowly added at 60 °C for 4 h. After cooling down to room temperature, the reaction mixture was filtered and washed with acetone. The filtrate was extracted with EtOAc (300 mL). The organic layer was washed three times with brine (120 mL × 3), dried over anhydrous Na_2_SO_4_, filtered and concentrated in vacuo. The crude product was purified via flash chromatography (hexane: EtOAc = 20:1) to afford **9** (5.25 g, 90% yield) as a white solid.

1-(2-hydroxy-4,6-dimethoxyphenyl)ethan-1-one **9**:

^1^H NMR (400 MHz, Acetone-*d*_6_) δ 13.97 (s, 1H), 6.07 (d, J = 2.7 Hz, 1H), 6.03 (d, J = 2.7 Hz, 1H), 3.92 (s, 3H), 3.85 (s, 3H), 2.56 (s, 3H).

^13^C NMR (100 MHz, Acetone-*d*_6_) δ 203.88, 168.43, 167.41, 164.21, 106.52, 94.44, 91.40, 56.08, 33.05.

HRMS (ESI): *m*/*z* calcd for C_10_H_12_O_4_ [M+H^+^]: 197.0808; found: 197.0801.

Compound **9** (2 g, 10.19 mmol) and N,N-dimethylformamide dimethylacetal (DMF-DMA) (2.71 mL, 20.39 mmol) were stirred in anhydrous DMF (25 mL) at 80 °C for 1 h. The resulting mixture was cooled to room temperature, and H_2_O was added to the reaction mixture. The aqueous layer was extracted three times with EtOAc (100 mL × 3). The combined organic layers were washed with brine (70 mL × 3), dried over anhydrous Na_2_SO_4_, filtered and concentrated in vacuo. The crude product was purified via flash chromatography (hexane: EtOAc = 15:1) to afford **10** (2.25 g, 88% yield) as a yellow solid.

(*E*)-3-(dimethylamino)-1-(2-hydroxy-4,6-dimethoxyphenyl)prop-2-en-1-one **10**:

^1^H NMR (400 MHz, CDCl_3_) δ 15.66 (s, 1H), 7.90 (d, J = 12.3 Hz, 1H), 6.24 (d, J = 12.3 Hz, 1H), 6.05 (d, J = 2.4 Hz, 1H), 5.89 (d, J = 2.4 Hz, 1H), 3.82 (s, 3H), 3.78 (s, 3H), 3.12 (s, 3H), 2.95–2.83 (s, 3H).

^13^C NMR (100 MHz, CDCl_3_) δ 189.95, 167.88, 164.00, 161.65, 154.36, 105.36, 96.76, 93.97, 90.54, 55.57, 55.34.

HRMS (ESI): *m*/*z* calcd for C_13_H_17_NO_4_ [M+H^+^]: 252.1230; found: 252.1219.

To a solution of compound **10** (2.50 g, 9.95 mmol) in MeOH (25 mL), iodine (3.28 g, 12.93 mmol) and pyridine (0.79 g, 9.95 mmol) were added in sequence at room temperature for 3 h. The solution was washed with saturated Na_2_S_2_O_3_ (30 mL), and the aqueous layer was extracted three times with DCM (100 mL × 3). The combined organic layers were washed with brine (30 mL × 3), dried over anhydrous Na_2_SO_4_, filtered and concentrated in vacuo. The crude product was purified via flash chromatography (hexane: EtOAc = 20:1) to afford **11** (1.31 g, 78% yield) as a yellow solid.

3-iodo-5,7-dimethoxy-4*H*-chromen-4-one **11**:

^1^H NMR (400 MHz, CDCl_3_) δ 8.06 (s, 1H), 6.41 (d, J = 2.3 Hz, 1H), 6.37 (d, J = 2.3 Hz, 1H), 3.91 (s, 3H), 3.87 (s, 3H).

^13^C NMR (100 MHz, CDCl_3_) δ 171.26, 164.26, 160.93, 159.80, 155.36, 107.49, 96.59, 92.44, 89.73, 56.42, 55.83.

HRMS (ESI): *m*/*z* calcd for C_11_H_9_O_4_l [M+H^+^]: 332.9618; found: 332.9611.

To a solution of compound **11** (1.20 g, 3.61 mmol) and (4-hydroxyphenyl)boronic acid (0.75 g, 5.42 mmol) in 20 mL of methanol at 50 °C, PdCl_2_(dppf) (0.26 g, 10 mol%) and K_2_CO_3_ (1.50 g, 10.84 mmol) were added. The resulting suspension was degassed and stirred in an inert atmosphere for 2 h before being concentrated in vacuo. The resulting crude product was purified via flash chromatography (hexane: EtOAc = 1:1) to afford **12** (0.93 g, 86% yield) as a white solid.

3-(4-hydroxyphenyl)-5,7-dimethoxy-4*H*-chromen-4-one **12**:

^1^H NMR (400 MHz, Acetone-*d*_6_) δ8.45 (s, 1H), 7.99 (s, 1H), 7.42 (d, J = 8.6 Hz, 2H), 6.88 (d, J = 8.6 Hz, 2H), 6.59 (d, J = 2.4 Hz, 1H), 6.50 (d, J = 2.4 Hz, 1H), 3.95 (s, 3H), 3.90 (s, 3H).

^13^C NMR (100 MHz, Acetone-*d*_6_) δ 178.98, 169.20, 166.73, 165.02, 162.41, 155.29, 135.56, 130.96, 129.29, 128.91, 120.02, 101.17, 97.88, 60.77, 60.59.

HRMS (ESI): *m*/*z* calcd for C_17_H_14_O_5_ [M+H^+^]: 299.0914; found: 299.0908.

The 40% HBr (aq) (10 mL) was added to compound **12** (0.50 g, 1.68 mmol) and the mixture was stirred at 140 °C for 4 h. EtOAc was added to the reaction mixture. The aqueous layer was extracted three times with EtOAc (50 mL × 3). The combined organic layers were washed with brine (30 mL × 3), dried over anhydrous Na_2_SO_4_, filtered and concentrated in vacuo. The crude product was purified via flash chromatography (hexane: EtOAc = 2:1) to afford **3** (0.34 g, 78% yield) as a yellow solid.

5,7-dihydroxy-3-(4-hydroxyphenyl)-4*H*-chromen-4-one **3**:

^1^H NMR (400 MHz, DMSO-*d*_6_) δ 12.95 (s, 1H), 10.87 (s, 1H), 9.59 (s, 1H), 8.30 (s, 1H), 7.37 (d, J = 6.7 Hz, 2H), 6.82 (d, J = 6.7 Hz, 2H), 6.38 (d, J = 2.1 Hz, 1H), 6.22 (d, J = 2.1 Hz, 1H).

^13^C NMR (100 MHz, DMSO-*d*_6_) δ 180.70, 164.75, 162.48, 158.07, 157.90, 154.42, 130.63, 122.77, 121.70, 115.54, 104.95, 99.44, 94.14.

HRMS (ESI): *m*/*z* calcd for C_15_H_10_O_5_ [M+H^+^]: 271.0601; found: 271.0601.

To a suspension of compound **3** (5 g, 18.50 mmol) in DMF (50 mL), K_2_CO_3_ (5.11 g, 37 mmol), KI (4.61 g, 27.75 mmol), CuI (0.18 g, 0.93 mmol) and 3-chloro-3-methylbut-1-yne (2.29 mL, 20.4 mmol) were added at room temperature for 5 h. The reaction mixture was quenched with saturated aqueous NH_4_Cl (20 mL). The layers were separated, and the aqueous layer was extracted three times with EtOAc (50 mL × 3). The combined organic layers were washed with brine (30 mL × 3), dried over anhydrous Na_2_SO_4_, filtered and concentrated in vacuo. The crude product was purified via flash chromatography (hexane: EtOAc = 30:1) to afford **13** (4.48 g, 72% yield) as a yellow solid.

5-hydroxy-3-(4-hydroxyphenyl)-7-((2-methylbut-3-yn-2-yl)oxy)-4*H*-chromen-4-one **13**:

^1^H NMR (400 MHz, CDCl_3_) δ 12.71 (s, 1H), 7.87 (s, 1H), 7.34 (d, J = 7.9 Hz, 2H), 6.94–6.77 (m, 3H), 6.71 (d, J = 2.3 Hz, 1H), 5.78 (s, 1H), 2.70 (s, 1H), 1.74 (s, 6H).

^13^C NMR (100 MHz, CDCl_3_) δ 181.21, 162.13, 161.98, 157.42, 156.14, 153.08, 130.35, 123.85, 122.74, 115.77, 106.81, 102.83, 97.36, 84.68, 75.35, 72.86, 29.57.

HRMS (ESI): *m*/*z* calcd for C_20_H_16_O_5_ [M+H^+^]: 337.1071; found: 337.1052.

A solution of compound **13** (1.50 g, 4.46 mmol) in diethylaniline (20 mL) was stirred at 250 °C for 1 h. The resulting mixture was cooled to room temperature and EtOAc was added to the reaction mixture. The organic layers were extracted three times with 1N HCl solution (100 mL × 3), and the organic layers were washed with brine (30 mL × 3), dried over anhydrous Na_2_SO_4_, filtered and concentrated in vacuo. The crude product was purified via flash chromatography (hexane: EtOAc = 20:1) to afford **14** (0.79 g, 52% yield) and **15** (0.62 g, 41% yield) as a yellow solid.

5-hydroxy-3-(4-hydroxyphenyl)-8,8-dimethyl-4*H*,8*H*-pyrano [2,3-f]chromen-4-one **14**:

^1^H NMR (400 MHz, CDCl_3_) δ 12.89 (s, 1H), 7.89 (s, 1H), 7.36 (d, J = 8.5 Hz, 2H), 6.86 (d, J = 8.5 Hz, 2H), 6.69 (d, J = 10.0 Hz, 1H), 6.31 (s, 1H), 5.60 (d, J = 10.0 Hz, 1H), 5.53 (s, 1H), 1.48 (s, 6H).

^13^C NMR (100 MHz, CDCl_3_) δ 181.12, 162.19, 159.68, 156.09, 152.56, 152.27, 130.37, 127.55, 123.75, 122.78, 115.72, 114.58, 106.06, 101.20, 100.43, 78.19, 28.23.

5-hydroxy-7-(4-hydroxyphenyl)-2,2-dimethyl-2*H*,6*H*-pyrano [3,2-g]chromen-6-one **15**:

^1^H NMR (400 MHz, CDCl_3_) δ 13.10 (s, 1H), 7.81 (s, 1H), 7.35 (d, J = 8.1 Hz, 2H), 6.84 (d, J = 8.1 Hz, 2H), 6.73 (d, J = 10.0 Hz, 1H), 6.34 (s, 1H), 5.62 (d, J = 10.0 Hz, 1H), 5.43 (s, 1H), 1.48 (s, 6H).

^13^C NMR (100 MHz, CDCl_3_) δ 181.06, 159.64, 157.39, 156.89, 156.02, 152.66, 130.36, 128.23, 123.64, 122.96, 115.70, 115.49, 106.14, 105.65, 94.93, 78.11, 28.32.

To a suspension of compound **15** (1 g, 2.97 mmol) in DCM (10 mL), tert-Butyldimethylsilyl chloride (TBSCl) (1.55 mL, 8.92 mmol) and imidazole (0.61 g, 8.92 mmol) were added at room temperature for 25 h before being concentrated in vacuo. The crude product was purified via flash chromatography (hexane: EtOAc = 200:1) to afford **23** (1.28 g, 95% yield) as a yellow solid.

7-(4-((tert-butyldimethylsilyl)oxy)phenyl)-5-hydroxy-2,2-dimethyl-2*H*,6*H*-pyrano [3,2-g]chromen-6-one **23**:

^1^H NMR (400 MHz, CDCl_3_) δ 13.18 (s, 1H), 7.82 (s, 1H), 7.39 (d, J = 8.6 Hz, 2H), 6.90 (d, J = 8.6 Hz, 2H), 6.73 (d, J = 10.1 Hz, 1H), 6.33 (s, 1H), 5.62 (d, J = 10.1 Hz, 1H), 1.47 (s, 6H), 1.00 (s, 9H), 0.22 (s, 6H).

^13^C NMR (100 MHz, CDCl_3_) δ 180.91, 159.52, 157.31, 156.97, 156.04, 152.60, 130.05, 128.18, 123.57, 123.62, 120.24, 115.52, 106.14, 105.59, 94.87, 78.05, 28.32, 25.69, 18.24, −4.36.

HRMS (ESI): *m*/*z* calcd for C_26_H_30_O_5_Si[M+H^+^]: 451.1935; found: 451.1950.

To a solution of **23** (1.80 g, 3.99 mmol) in anhydrous DMF (30 mL), NaH (0.19 g, 4.79 mmol) and 3,3-dimethylallyl bromide **5** (0.51 mL, 4.40 mmol) were added sequentially. The resulting mixture was stirred at room temperature for 1 h. It was then quenched with brine (60 mL). The aqueous layer was extracted three times with EtOAc (100 mL × 3). The combined organic layers were washed three times with brine (50 mL × 3), dried over anhydrous Na_2_SO_4_, filtered and concentrated in vacuo. The crude product was purified via flash chromatography (hexane: EtOAc = 50:1) to afford **24** (2.22 g, 74% yield) as a yellow solid.

7-(4-((tert-butyldimethylsilyl)oxy)phenyl)-2,2-dimethyl-5-((3-methylbut-2-en-1-yl)oxy)-2*H*,6*H*-pyrano [3,2-g]chromen-6-one **24**:

^1^H NMR (400 MHz, CDCl_3_) δ 7.75 (d, J = 2.0 Hz, 1H), 7.40 (d, J = 8.6 Hz, 2H), 6.88 (d, J = 8.6Hz, 2H), 6.74 (d, J = 10.1 Hz, 1H), 6.59 (s, 1H), 5.68 (d, J = 10.1 Hz, 1H), 5.65–5.56 (m, 1H), 4.57 (d, J = 7.4 Hz, 2H), 1.75 (s, 3H), 1.64 (s, 3H), 1.46 (s, 6H), 0.99 (s, 9H), 0.22 (s, 6H).

^13^C NMR (100 MHz, CDCl_3_) δ 175.11, 158.70, 157.86, 155.66, 154.65, 150.47, 138.23, 130.28, 130.13, 125.61, 125.03, 120.43, 120.01, 117.09, 114.01, 113.50, 100.54, 77.56, 72.21, 28.26, 25.89, 25.71, 18.23, 18.11, −4.35.

HRMS (ESI): *m*/*z* calcd for C_31_H_38_O_5_Si[M+H^+^]: 519.2561; found: 519.2554.

To a solution of **24** (1.2 g, 2.31 mmol) in anhydrous chloroform (30 mL), Eu(fod)_3_ (21 mg, 0.015 mmol) was added at 60 °C for 20 h, and then concentrated on a rotary evaporator. The crude product was subjected to flash chromatography (hexane: EtOAc = 600:1) to afford **25** (0.92 g, 76% yield) as a yellow solid.

7-(4-((tert-butyldimethylsilyl)oxy)phenyl)-5-hydroxy-2,2-dimethyl-10-(3-methylbut-2-en-1-yl)-2*H*,6*H*-pyrano [3,2-g]chromen-6-one **25**:

^1^H NMR (400 MHz, CDCl_3_) δ 13.12 (s, 1H), 7.90 (s, 1H), 7.40 (d, J = 8.5 Hz, 2H), 6.90 (d, J = 8.5 Hz, 2H), 6.74 (d, J = 10.2 Hz, 1H), 5.62 (d, J = 10.2 Hz, 1H), 5.18 (t, J = 7.4 Hz, 1H), 3.40 (d, J = 7.4 Hz, 2H), 1.81 (s, 3H), 1.68 (s, 3H), 1.47 (s, 6H), 1.00 (s, 9H), 0.22 (s, 6H).

^13^C NMR (100 MHz, CDCl_3_) δ 181.26, 156.89, 155.97, 154.98, 154.73, 152.63, 131.70, 130.04, 128.01, 123.81, 123.24, 122.02, 120.22, 115.91, 107.44, 105.95, 105.45, 77.80, 28.23, 25.80, 25.70, 21.31, 18.24, 17.91, −4.36.

HRMS (ESI): *m*/*z* calcd for C_31_H_38_O_5_Si[M+H^+^]: 519.2561; found: 519.2574.

To a solution of **25** (0.8 g, 1.54 mmol) in anhydrous THF (15 mL), tetrabutylammonium fluoride (TBAF) (1 M solution in THF, 15 mL, 15 mmol) was added. The resulting solution was stirred at room temperature for 0.5 h. The reaction mixture was poured into 50 mL ice water and stirred for 20 min. The aqueous layer was extracted three times with EtOAc (100 mL × 3). The combined organic layers were washed three times with brine (70 mL × 3), dried over anhydrous Na_2_SO_4_, filtered and concentrated in vacuo. The crude product was purified via flash chromatography (hexane: EtOAc = 20:1) to afford **1** (0.58 g, 93% yield) as a white solid.

NMR Data from Supplementary Table S1.

HRMS (ESI): *m*/*z* calcd for C_25_H_24_O_5_[M+H^+^]: 405.1697; found: 405.1636.

3-(4-((tert-butyldimethylsilyl)oxy)phenyl)-5-hydroxy-8,8-dimethyl-4*H*,8*H*-pyrano [2,3-f]chromen-4-one 16: Compound 16 was synthesized by following a similar procedure as that of **23**.

^1^H NMR (400 MHz, CDCl_3_) δ 12.95 (s, 1H), 7.89 (s, 1H), 7.49–7.34 (d, J = 8.6 Hz, 2H), 6.91 (d, J = 8.6 Hz, 2H), 6.68 (d, J = 10.0 Hz, 1H), 6.29 (s, 1H), 5.59 (d, J = 10.0 Hz, 1H), 1.47 (s, 6H), 1.00 (s, 9H), 0.22 (s, 6H).

^13^C NMR (100 MHz, CDCl_3_) δ 180.95, 162.31, 159.58, 156.10, 152.45, 152.28, 130.07, 127.47, 123.71, 123.50, 120.25, 114.62, 106.08, 101.13, 100.36, 78.11, 28.23, 25.69, 18.24, −4.36.

HRMS (ESI): *m*/*z* calcd for C_26_H_30_O_5_Si[M+H^+^]: 451.1935; found: 451.1950.

3-(4-((tert-butyldimethylsilyl)oxy)phenyl)-8,8-dimethyl-5-((3-methylbut-2-en-1-yl)oxy)-4*H*,8*H*-pyrano [2,3-f]chromen-4-one 17: Compound 17 was synthesized by following a similar procedure as that of **24**.

^1^H NMR (400 MHz, CDCl_3_) δ 7.75 (s, 1H), 7.39 (d, J = 8.4 Hz, 2H), 6.85 (d, J = 8.4 Hz, 2H), 6.72 (d, J = 10.0 Hz, 1H), 6.31 (s, 1H), 5.73–5.43 (m, 2H), 4.61 (d, J = 6.4 Hz, 2H), 1.76 (s, 3H), 1.72 (s, 3H), 1.48 (s, 6H), 0.98 (s, 9H), 0.20 (s, 6H).

^13^C NMR (100 MHz, CDCl_3_) δ 175.44, 160.53, 157.51, 155.57, 154.14, 149.75, 137.46, 130.43, 127.25, 125.93, 125.06, 119.88, 119.38, 115.19, 109.79, 102.27, 97.59, 77.97, 66.46, 28.23, 25.81, 25.72, 18.40, 18.23, −4.36.

HRMS (ESI): *m*/*z* calcd for C_26_H_30_O_5_Si[M+H^+^]: 519.2561; found: 519.2554.

To a solution of compound **17** (2.5 g, 4.82 mmol) in dry DCM (30 mL), montmorillonite K10 (2.5 g, 1 wt) was added under argon. After stirring for 1 h at room temperature, the reaction mixture was filtered and concentrated. The crude product was purified via flash chromatography (hexane: EtOAc = 600:1) to afford **18** (2.23 g, 89% yield) as a yellow solid.

3-(4-((tert-butyldimethylsilyl)oxy)phenyl)-5-hydroxy-8,8-dimethyl-6-(3-methylbut-2-en-1-yl)-4*H*,8*H*-pyrano [2,3-f]chromen-4-one **18**:

^1^H NMR (400 MHz, CDCl_3_) δ 13.17 (s, 1H), 7.87 (s, 1H), 7.39 (d, J = 8.7 Hz, 2H), 6.91 (d, J = 8.7 Hz, 2H), 6.70 (d, J = 10.0 Hz, 1H), 5.59 (d, J = 10.0 Hz, 1H), 5.33–5.09 (m, 1H), 3.35 (d, J = 7.3 Hz, 2H), 1.81 (s, 3H), 1.68 (s, 3H), 1.48 (s, 6H), 1.00 (s, 9H), 0.23 (s, 6H).

^13^C NMR (100 MHz, CDCl_3_) δ 180.97, 159.40, 157.21, 155.98, 152.29, 150.52, 131.63, 130.08, 127.14, 123.81, 123.49, 121.98, 120.22, 115.02, 112.83, 105.63, 100.73, 77.86, 28.15, 25.84, 25.70, 21.32, 18.24, 17.96, −4.35.

5-hydroxy-3-(4-hydroxyphenyl)-8,8-dimethyl-6-(3-methylbut-2-en-1-yl)-4H,8H-pyrano [2,3-f]chromen-4-one 2: Compound 2 was synthesized by following a similar procedure as that of **1**.

NMR Data from Supplementary Table S2.

HRMS (ESI): *m*/*z* calcd for C_25_H_24_O_5_[M+H^+^]: 405.1697; found: 405.1703.

## 4. Conclusions

In conclusion, we have accomplished the total synthesis of osajin, scandenone and analogues from commercially available starting materials. The key reactions for the preparation of these compounds involve the aldol reaction, Claisen rearrangement, Schenck ene reaction and Suzuki coupling reaction. Additionally, we have designed and synthesized twenty-five natural product analogues, which were screened for anti-inflammatory activity against TNF-α and IL-6 in LPS-stimulated RAW264.7 macrophages. From this series of compounds, compounds **39e** and **39d** emerged as promising lead candidates for the development of novel anti-inflammatory drugs.

## Figures and Tables

**Table 1:** Optimization of the Suzuki coupling reactions ^a^.

Entry	Catalyst	Solvent	Base	Temperature (°C)	Yield (%) ^c^
1	Pd(OAc)_2_	MeOH	Na_2_CO_3_	rt ^b^	40
2	Pd(OAc)_2_	DMF	Na_2_CO_3_	rt	45
3	Pd(OAc)_2_	DMF	K_2_CO_3_	rt	43
4	Pd(OAc)_2_	DMF	K_2_CO_3_	50	60
5	Pd(PPh_3_)_4_	DMF	K_2_CO_3_	rt	46
6	Pd(PPh_3_)_4_	DMF	K_2_CO_3_	40	57
7	Pd(PPh_3_)_4_	DMF	K_2_CO_3_	60	67
8	Pd(PPh_3_)_4_	DMF	K_2_CO_3_	80	70
9	PdCl_2_(dppf)	1,4-Dioxane/H_2_O ^d^	K_2_CO_3_	50	84
10	PdCl_2_(dppf)	1,4-Dioxane/H_2_O	K_2_CO_3_	80	86

^a^ Standard conditions: **11** (0.05 mmol) and palladium catalyst in solvent (5 mL) under argon. ^b^ rt: room temperature. ^c^ Yield of the isolated product. ^d^ The ratio of 1,4-Dioxane to H_2_O is 3:1.

## Data Availability

Data is contained within the article and Supplementary Material.

## Supplementary Materials

The following supporting information can be downloaded at: https://www.mdpi.com/article/10.3390/ph17010086/s1, Table S1: Comparison of ^1^H NMR Spectral Data. Table S2: Comparison of ^1^H NMR Spectral Data. Scheme S1: Previous synthesis of osajin and scandenone. Figure S1: Cell viability of RAW264.7 macrophages induced with synthetic compounds after 24 h via CCK-8 assay. Experimental procedures and characterization data (^1^H NMR, ^13^C NMR and HRMS^n^) can be found in the Supplementary Materials.
