# Supplementary material for: Total Synthesis and Anti-Inflammatory Evaluation of Osajin, Scandenone and Analogues
Source: Pharmaceuticals (Basel). 2024 Jan 9;17(1):86. doi: 10.3390/ph17010086 (PMC10819276; doi:10.3390/ph17010086)
Supplement: Supplementary file 1 [file pharmaceuticals-17-00086-s001.zip › pharmaceuticals-2802002-supplementary.pdf]

Total Synthesis and Anti-inflammatory Evaluation of Osajin, Scandenone and Analogues

Rui Wang [1], Ran Ma [1,2], Ke Feng [1], Hongchen Lu [1,2], Wei Zhao [1,*] and Hongzhen Jin [1,3,*]

1 State Key Laboratory of Medicinal Chemical Biology, Key Laboratory of Molecular Drug Research and KLMDASR of Tianjin, College of Pharmacy, Nankai University, Haihe Education Park, Tongyan Road, Tianjin 300350, China
2 Tianjin International Joint Academy of Biomedicine, Tianjin 300457, China
3 School of Health and Life Sciences, University of Health and Rehabilitation Sciences, Qingdao 266071, China
* Correspondence: wzhao@nankai.edu.cn (W.Z.); jinhongzhen@nankai.edu.cn (H.J.)

Table of Contents

1. General Information……………………………………………3
2. Comparison of ^{1}H NMR Spectra of Scandenone and Osajin..…………………………………………………………3
3. Synthesis of osajin and scandenone...……………………………..4
4. Experimental Procedures………………………………………4
5. Biological study…………………………………………………29
6. NMR Spectra.…………………………………………………..29
7. References……………………………………………………..110

1. General Information

All reactions were carried out under an inert nitrogen atmosphere with dry solvents under anhydrous conditions unless otherwise stated. Flash chromatography was performed using Silica gel (200-400 mesh). Thin layer chromatography (TLC) was performed using Silica gel 60 F254 plates and visualized using UV light.
^{1}H and ^{13}C spectra were recorded with Bruker Avance II 400 [400 MHz] and calibrated using residual solvent as an internal reference [^{1}H NMR: $CDCl_3$ (7.26); ^{13}C NMR: $CDCl_3$ (77.16); DMSO-d_6 = 2.50; DMSO-d_6 = 39.52]. Signals were described as s = singlet, d = doublet, t = triplet, q = quartet, m = multiplet. High resolution mass spectra (HRMS) were recorded on an IonSpec QFT mass spectrometer with ESI ionization.

2. Comparison of ^{1}H NMR Spectra of Scandenone and Osajin[1]

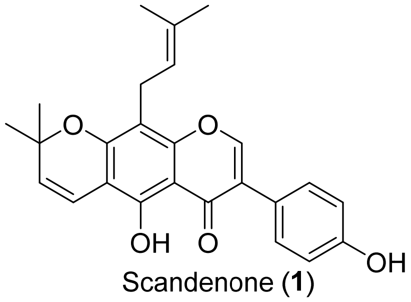

Scandenone (**1**)

Table S1. Comparison of ^{1}H NMR Spectral Data.

Position	Isolated Scandenone (400 MHz, $CDCl_3$) δ_1 (ppm)	Synthetic Scandenone (400 MHz, $CDCl_3$) δ_2(ppm)	Deviation $\Delta\delta = \delta_1 - \delta_2$ (ppm)
2	7.89	7.89	0
2'/6'	7.35	7.33	0.02
3'/5'	6.84	6.82	0.02
5-OH		13.03	
4''	6.74	6.74	0
5''	5.63	5.63	0
5''-CH_3	1.47	1.47	0
1'''	3.40	3.40	0
2'''	5.17	5.18	-0.01
4'''	1.81	1.82	0
5'''	1.68	1.69	0

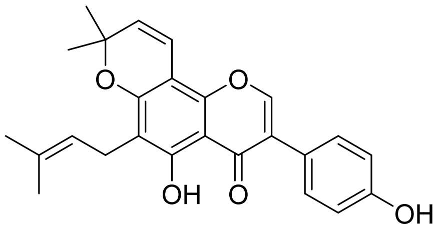

Osajin (**2**)

Table S2. Comparison of ^{1}H NMR Spectral Data.

Position	Isolated osajin (400 MHz, $CDCl_3$) δ_1 (ppm)	Synthetic osajin (400 MHz, $CDCl_3$) δ_2(ppm)	Deviation $\Delta\delta = \delta_1 - \delta_2$ (ppm)
2	7.88	7.88	0
5-OH	5.00	5.02	-0.02
2'/6'	7.39	7.39	0
3'/5'	6.89	6.89	0
5-OH	13.14	13.14	0
4''	6.70	6.70	0
5''	5.59	5.61	-0.02
6''-CH_3	1.50	1.50	0
1'''	3.37	3.37	0
2'''	5.26	5.26	0
4'''	1.83	1.83	0
5'''	1.70	1.70	0

3. Synthesis of osajin and scandenone[2].

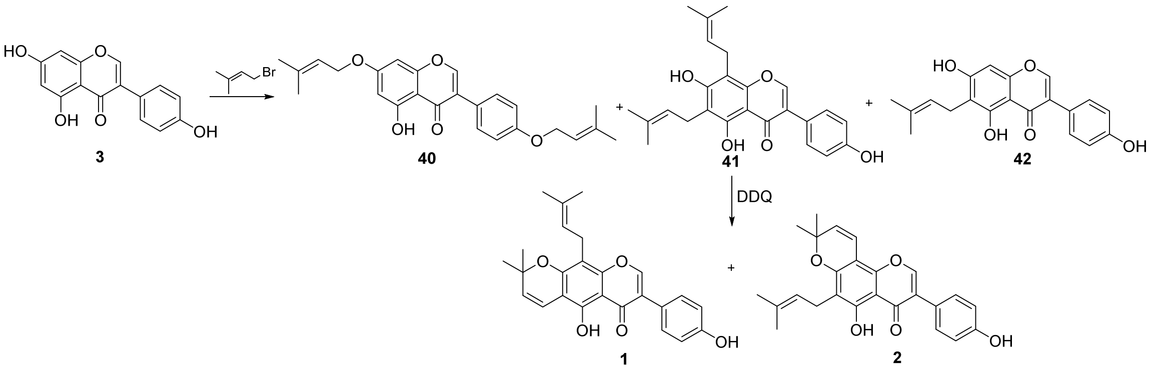

Scheme S1. Previous synthesis of osajin and scandenone.

4. Experimental Procedures

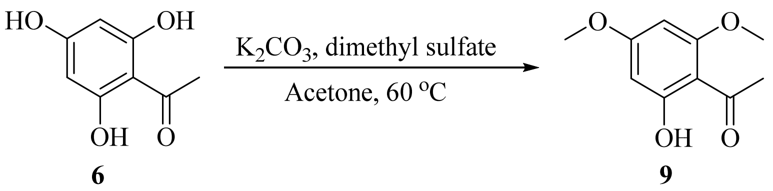

To a stirred soulution of 1-(2,4,6-trihydroxyphenyl)ethan-1-one (5.00 g, 29.73 mmol) and K_2CO_3 (9.04 g, 65.44 mmol) in anhydrous acetone (80 mL) was slowly added dimethyl sulfate (5.78 mL, 60.95 mmol) at 60 °C for 4 h. After cooling down to room temperature, the reaction mixture was filtered and washed with acetone. The filtrate was extracted with EtOAc (300 mL). The organic layer was washed three times with brine (120 mL × 3), dried over anhydrous Na_2SO_4, filtered and concentrated in vacuo. The crude product was purified by flash chromatography (hexane : EtOAc = 20:1) to afford **9** (5.25 g, 90% yield) as a white solid.

1-(2-hydroxy-4,6-dimethoxyphenyl)ethan-1-one 9:

^{1}H NMR (400 MHz, Acetone-d_6) δ 13.97 (s, 1H), 6.07 (d, J = 2.7 Hz, 1H), 6.03 (d, J = 2.7 Hz, 1H), 3.92 (s, 3H), 3.85 (s, 3H), 2.56 (s, 3H).

^{13}C NMR (100 MHz, Acetone-d_6) δ 203.88, 168.43, 167.41, 164.21, 106.52, 94.44, 91.40, 56.08, 33.05.

HRMS (ESI): m/z calcd for $C_{10}H_{12}O_4$ [M+H$^+$]: 197.0808; found: 197.0801.

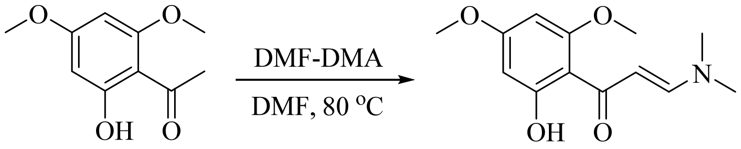

9 **10**

Compound **9** (2 g, 10.19 mmol) and *N*,*N*-dimethylformamide dimethylacetal (DMF-DMA) (2.71 ml, 20.39 mmol) were stirred in anhydrous DMF (25 mL) at 80 °C for 1 h. The resulting mixture was cooled to room temperature and H_2O was added to the reaction mixture. The aqueous layer was extracted three times with EtOAc (100 mL × 3). The combined organic layers were washed with brine (70 mL × 3), dried over anhydrous Na_2SO_4, filtered and concentrated in vacuo. The crude product was purified by flash chromatography (hexane : EtOAc = 15:1) to afford **10** (2.25 g, 88% yield) as a yellow solid.

(*E*)-3-(dimethylamino)-1-(2-hydroxy-4,6-dimethoxyphenyl)prop-2-en-1-one 10:

^{1}H NMR (400 MHz, $CDCl_3$) δ 15.66 (s, 1H), 7.90 (d, *J* = 12.3 Hz, 1H), 6.24 (d, *J* = 12.3 Hz, 1H), 6.05 (d, *J* = 2.4 Hz, 1H), 5.89 (d, *J* = 2.4 Hz, 1H), 3.82 (s, 3H), 3.78 (s, 3H), 3.12 (s, 3H), 2.95 – 2.83 (s, 3H).

^{13}C NMR (100 MHz, $CDCl_3$) δ 189.95, 167.88, 164.00, 161.65, 154.36, 105.36, 96.76, 93.97, 90.54, 55.57, 55.34.

HRMS (ESI): m/z calcd for $C_{13}H_{17}NO_4$ [M+H$^+$]: 252.1230; found: 252.1219.

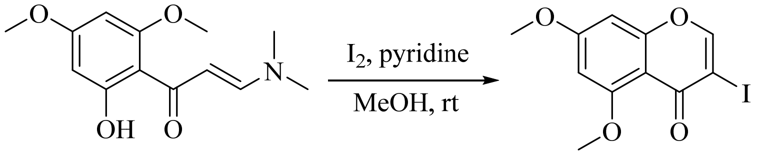

10 **11**

To a solution of compound **10** (2.50 g, 9.95 mmol) in MeOH (25 mL) was added iodine (3.28 g, 12.93 mmol) and pyridine (0.79 g, 9.95 mmol) in sequence at room temperature for 3 h. The solution was washed with saturated $Na_2S_2O_3$ (30 mL) and the aqueous layer was extracted three times with

DCM (100 mL × 3). The combined organic layers were washed with brine (30 mL × 3), dried over anhydrous Na_2SO_4, filtered and concentrated in vacuo. The crude product was purified by flash chromatography (hexane : EtOAc = 20:1) to afford **11** (1.31 g, 78% yield) as a yellow solid.

3-iodo-5,7-dimethoxy-4*H*-chromen-4-one 11:

^{1}H NMR (400 MHz, $CDCl_3$) δ 8.06 (s, 1H), 6.41 (d, J = 2.3 Hz, 1H), 6.37 (d, J = 2.3 Hz, 1H), 3.91 (s, 3H), 3.87 (s, 3H).

^{13}C NMR (100 MHz, $CDCl_3$) δ 171.26, 164.26, 160.93, 159.80, 155.36, 107.49, 96.59, 92.44, 89.73, 56.42, 55.83.

HRMS (ESI): m/z calcd for $C_{11}H_9O_4I$ [M+H$^+$]: 332.9618; found: 332.9611.

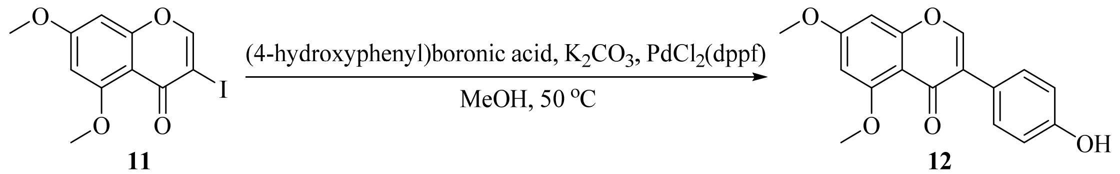

11 **12**

To a solution of compound **11** (1.20 g, 3.61 mmol) and (4-hydroxyphenyl)boronic acid (0.75 g, 5.42 mmol) in 20 mL of methanol at 50 °C was added $PdCl_2$(dppf) (0.26 g, 10 mol%) and K_2CO_3 (1.50 g, 10.84 mmol). The resulting suspension was degassed and stirred in an inert atmosphere for 2 h before concentrated in vacuo. The resulting crude product was purified by flash chromatography (hexane : EtOAc = 1:1) to afford **12** (0.93 g, 86% yield) as a white solid[3].

3-(4-hydroxyphenyl)-5,7-dimethoxy-4*H*-chromen-4-one 12:

^{1}H NMR (400 MHz, Acetone-d_6) δ8.45 (s, 1H), 7.99 (s, 1H), 7.42 (d, J = 8.6 Hz, 2H), 6.88 (d, J = 8.6 Hz, 2H), 6.59 (d, J = 2.4 Hz, 1H), 6.50 (d, J = 2.4 Hz, 1H), 3.95 (s, 3H), 3.90 (s, 3H).

^{13}C NMR (100 MHz, Acetone-d_6) δ 178.98, 169.20, 166.73, 165.02, 162.41, 155.29, 135.56, 130.96, 129.29, 128.91, 120.02, 101.17, 97.88, 60.77, 60.59.

HRMS (ESI): m/z calcd for $C_{17}H_{14}O_5$ [M+H$^+$]: 299.0914; found: 299.0908.

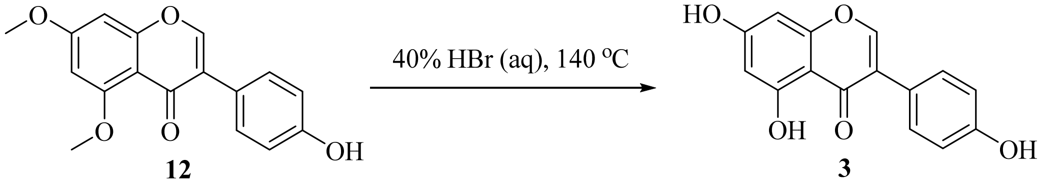

12 **3**

The 40% HBr (aq) (10 mL) was added to compound **12** (0.50 g, 1.68 mmol) and the mixture was stirred at 140 °C for 4 h. EtOAc was added to the reaction mixture. The aqueous layer was extracted three times with EtOAc (50 mL × 3). The combined organic layers were washed with brine (30 mL

× 3), dried over anhydrous Na_2SO_4, filtered and concentrated in vacuo. The crude product was purified by flash chromatography (hexane : EtOAc = 2:1) to afford **3** (0.34 g, 78% yield) as a yellow solid.

5,7-dihydroxy-3-(4-hydroxyphenyl)-4*H*-chromen-4-one 3:

^{1}H NMR (400 MHz, DMSO-d_6) δ 12.95 (s, 1H), 10.87 (s, 1H), 9.59 (s, 1H), 8.30 (s, 1H), 7.37 (d, J = 6.7 Hz, 2H), 6.82 (d, J = 6.7 Hz, 2H), 6.38 (d, J = 2.1 Hz, 1H), 6.22 (d, J = 2.1 Hz, 1H).

^{13}C NMR (100 MHz, DMSO-d_6) δ 180.70, 164.75, 162.48, 158.07, 157.90, 154.42, 130.63, 122.77, 121.70, 115.54, 104.95, 99.44, 94.14.

HRMS (ESI): m/z calcd for $C_{15}H_{10}O_5$ [M+H$^+$]: 271.0601; found: 271.0601.

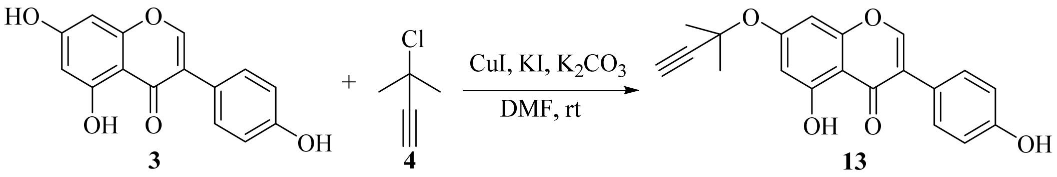

To a suspension of compound **3** (5 g, 18.50 mmol) in DMF (50 mL) was added K_2CO_3 (5.11 g, 37 mmol), KI (4.61 g, 27.75 mmol), CuI (0.18 g, 0.93 mmol) and 3-chloro-3-methylbut-1-yne (2.29 mL, 20.4 mmol) at room temperature for 5 h. The reaction mixture was quenched by saturated aqueous NH_4Cl (20 mL). The layers were separated, and the aqueous layer was extracted three times with EtOAc (50 mL × 3). The combined organic layers were washed with brine (30 mL × 3), dried over anhydrous Na_2SO_4, filtered and concentrated in vacuo. The crude product was purified by flash chromatography (hexane : EtOAc = 30:1) to afford **13** (4.48 g, 72% yield) as a yellow solid.

5-hydroxy-3-(4-hydroxyphenyl)-7-((2-methylbut-3-yn-2-yl)oxy)-4*H*-chromen-4-one 13:

^{1}H NMR (400 MHz, $CDCl_3$) δ 12.71 (s, 1H), 7.87 (s, 1H), 7.34 (d, J = 7.9 Hz, 2H), 6.94 – 6.77 (m, 3H), 6.71 (d, J = 2.3 Hz, 1H), 5.78 (s, 1H), 2.70 (s, 1H), 1.74 (s, 6H).

^{13}C NMR (100 MHz, $CDCl_3$) δ 181.21, 162.13, 161.98, 157.42, 156.14, 153.08, 130.35, 123.85, 122.74, 115.77, 106.81, 102.83, 97.36, 84.68, 75.35, 72.86, 29.57.

HRMS (ESI): m/z calcd for $C_{20}H_{16}O_5$ [M+H$^+$]: 337.1071; found: 337.1052.

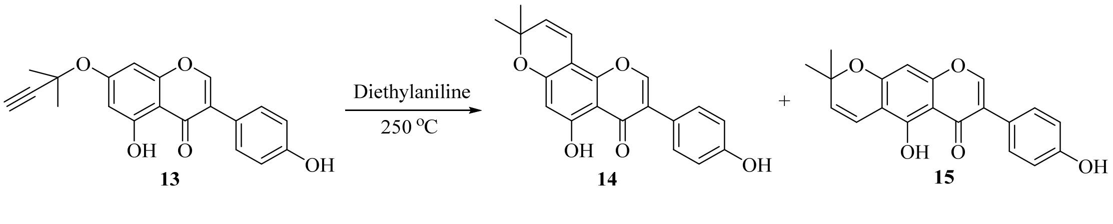

A solution of compound **13** (1.50 g, 4.46 mmol) in diethylaniline (20 mL) was stirred at 250 ºC for 1 h. The resulting mixture was cooled to room temperature and EtOAc was added to the reaction mixture. The organic layers was extracted three times with 1N HCl solution (100 mL × 3) and the organic layers were washed with brine (30 mL × 3), dried over anhydrous Na_2SO_4, filtered and concentrated in vacuo. The crude product was purified by flash chromatography (hexane : EtOAc = 20:1) to afford **14** (0.79 g, 52% yield) and **15** (0.62 g, 41% yield) as a yellow solid.

5-hydroxy-3-(4-hydroxyphenyl)-8,8-dimethyl-4*H*,8*H*-pyrano[2,3-f]chromen-4-one 14:

^{1}H NMR (400 MHz, $CDCl_3$) δ 12.89 (s, 1H), 7.89 (s, 1H), 7.36 (d, J = 8.5 Hz, 2H), 6.86 (d, J = 8.5 Hz, 2H), 6.69 (d, J = 10.0 Hz, 1H), 6.31 (s, 1H), 5.60 (d, J = 10.0 Hz, 1H), 5.53 (s, 1H), 1.48 (s, 6H).

^{13}C NMR (100 MHz, $CDCl_3$) δ 181.12, 162.19, 159.68, 156.09, 152.56, 152.27, 130.37, 127.55, 123.75, 122.78, 115.72, 114.58, 106.06, 101.20, 100.43, 78.19, 28.23.

5-hydroxy-7-(4-hydroxyphenyl)-2,2-dimethyl-2*H*,6*H*-pyrano[3,2-g]chromen-6-one 15:

^{1}H NMR (400 MHz, $CDCl_3$) δ 13.10 (s, 1H), 7.81 (s, 1H), 7.35 (d, J = 8.1 Hz, 2H), 6.84 (d, J = 8.1 Hz, 2H), 6.73 (d, J = 10.0 Hz, 1H), 6.34 (s, 1H), 5.62 (d, J = 10.0 Hz, 1H), 5.43 (s, 1H), 1.48 (s, 6H).

^{13}C NMR (100 MHz, $CDCl_3$) δ 181.06, 159.64, 157.39, 156.89, 156.02, 152.66, 130.36, 128.23, 123.64, 122.96, 115.70, 115.49, 106.14, 105.65, 94.93, 78.11, 28.32.

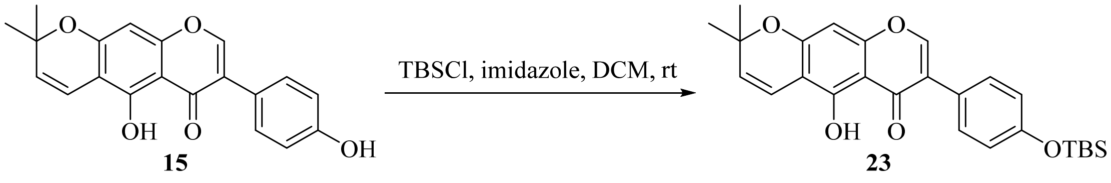

To a suspension of compound **15** (1 g, 2.97 mmol) in DCM (10 mL) was added tert-butyldimethylsilyl chloride (TBSCl) (1.55 mL, 8.92 mmol) and imidazole (0.61 g, 8.92 mmol) at room temperature for 25 h before concentrated in vacuo. The crude product was purified by flash chromatography (hexane : EtOAc = 200:1) to afford **23** (1.28 g, 95% yield) as a yellow solid.

7-(4-((tert-butyldimethylsilyl)oxy)phenyl)-5-hydroxy-2,2-dimethyl-2*H*,6*H*-pyrano[3,2-g]chromen-6-one 23:

^{1}H NMR (400 MHz, $CDCl_3$) δ 13.18 (s, 1H), 7.82 (s, 1H), 7.39 (d, *J* = 8.6 Hz, 2H), 6.90 (d, *J* = 8.6 Hz, 2H), 6.73 (d, *J* = 10.1 Hz, 1H), 6.33 (s, 1H), 5.62 (d, *J* = 10.1 Hz, 1H), 1.47 (s, 6H), 1.00 (s, 9H), 0.22 (s, 6H).

^{13}C NMR (100 MHz, $CDCl_3$) δ 180.91, 159.52, 157.31, 156.97, 156.04, 152.60, 130.05, 128.18, 123.57, 123.62, 120.24, 115.52, 106.14, 105.59, 94.87, 78.05, 28.32, 25.69, 18.24, -4.36.

HRMS (ESI): m/z calcd for $C_{26}H_{30}O_5Si[M+H^+]$: 451.1935; found: 451.1950.

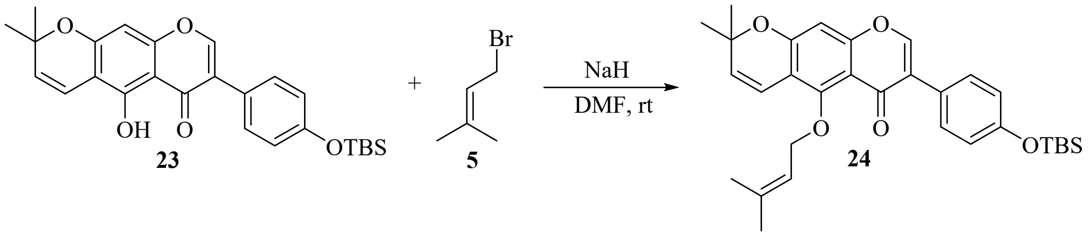

To a solution of 23 (1.80 g, 3.99 mmol) in anhydrous DMF (30 mL) was added NaH (0.19 g, 4.79 mmol) and 3,3-dimethylallyl bromide 5 (0.51 mL, 4.40 mmol) sequentially. The resulting mixture was stirred at room temperature for 1 h. It was then quenched by brine (60 mL). The aqueous layer was extracted three times with EtOAc (100 mL × 3). The combined organic layers were washed three times with brine (50 mL × 3), dried over anhydrous Na2SO4, filtered and concentrated in vacuo. The crude product was purified by flash chromatography (hexane : EtOAc = 50:1) to afford 24 (2.22 g, 74% yield) as a yellow solid.

7-(4-((tert-butyldimethylsilyl)oxy)phenyl)-2,2-dimethyl-5-((3-methylbut-2-en-1-yl)oxy)-2*H*,6*H*-pyrano[3,2-g]chromen-6-one 24:

^{1}H NMR (400 MHz, $CDCl_3$) δ 7.75 (d, *J* = 2.0 Hz, 1H), 7.40 (d, *J* = 8.6 Hz, 2H), 6.88 (d, *J* = 8.6Hz, 2H), 6.74 (d, *J* = 10.1 Hz, 1H), 6.59 (s, 1H), 5.68 (d, *J* = 10.1 Hz, 1H), 5.65 – 5.56 (m, 1H), 4.57 (d, *J* = 7.4 Hz, 2H), 1.75 (s, 3H), 1.64 (s, 3H), 1.46 (s, 6H), 0.99 (s, 9H), 0.22 (s, 6H).

^{13}C NMR (100 MHz, $CDCl_3$) δ 175.11, 158.70, 157.86, 155.66, 154.65, 150.47, 138.23, 130.28, 130.13, 125.61, 125.03, 120.43, 120.01, 117.09, 114.01, 113.50, 100.54, 77.56, 72.21, 28.26, 25.89, 25.71, 18.23, 18.11, -4.35.

HRMS (ESI): m/z calcd for $C_{31}H_{38}O_5Si[M+H^+]$: 519.2561; found: 519.2554.

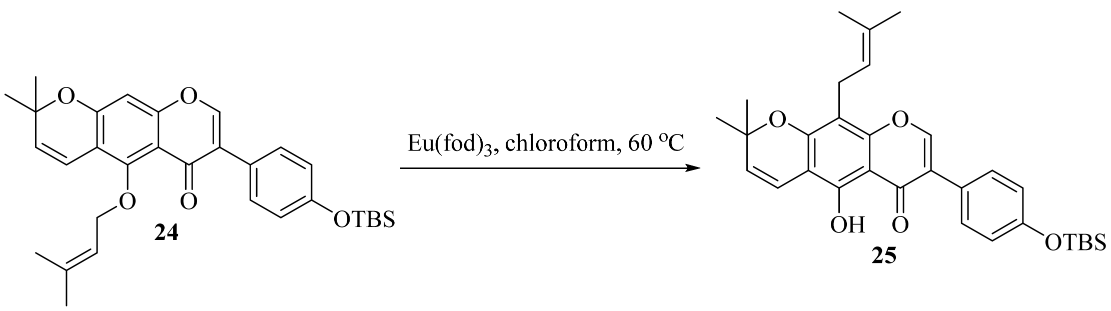

To a solution of 24 (1.2 g, 2.31 mmol) in anhydrous chloroform (30 mL) was added Eu(fod)3 (21 mg, 0.015 mmol) at 60 °C for 20 h, and then concentrated on a rotary evaporator. The crude product was subjected to flash chromatography (hexane : EtOAc = 600:1) to afford 25 (0.92 g, 76% yield) as a yellow solid.

7-(4-((tert-butyldimethylsilyl)oxy)phenyl)-5-hydroxy-2,2-dimethyl-10-(3-methylbut-2-en-1-yl)-2*H*,6*H*-pyrano[3,2-g]chromen-6-one 25:

^{1}H NMR (400 MHz, $CDCl_3$) δ 13.12 (s, 1H), 7.90 (s, 1H), 7.40 (d, J = 8.5 Hz, 2H), 6.90 (d, J = 8.5 Hz, 2H), 6.74 (d, J = 10.2 Hz, 1H), 5.62 (d, J = 10.2 Hz, 1H), 5.18 (t, J = 7.4 Hz, 1H), 3.40 (d, J = 7.4 Hz, 2H), 1.81 (s, 3H), 1.68 (s, 3H), 1.47 (s, 6H), 1.00 (s, 9H), 0.22 (s, 6H).

^{13}C NMR (100 MHz, $CDCl_3$) δ 181.26, 156.89, 155.97, 154.98, 154.73, 152.63, 131.70, 130.04, 128.01, 123.81, 123.24, 122.02, 120.22, 115.91, 107.44, 105.95, 105.45, 77.80, 28.23, 25.80, 25.70, 21.31, 18.24, 17.91, -4.36.

HRMS (ESI): m/z calcd for $C_{31}H_{38}O_5Si[M+H^+]$: 519.2561; found: 519.2574.

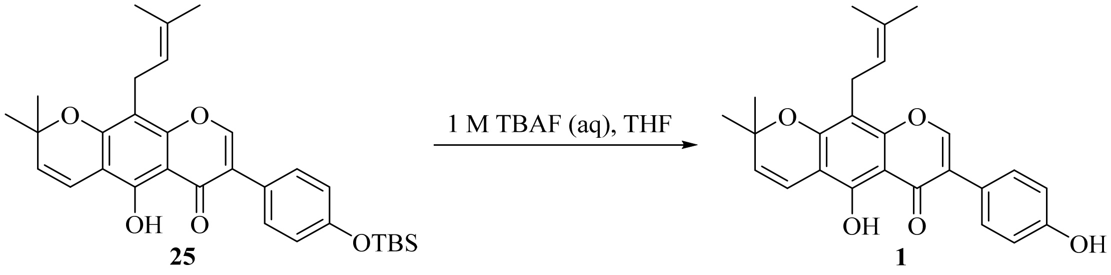

To a solution of **25** (0.8 g, 1.54 mmol) in anhydrous THF (15 mL) was added tetrabutylammonium fluoride (TBAF) (1 M solution in THF, 15 mL, 15 mmol). The resulting solution was stirred at room temperature for 0.5 h. The reaction mixture was poured into 50 mL ice water and stirred for 20 minutes. The aqueous layer was extracted three times with EtOAc (100 mL × 3). The combined organic layers were washed three times with brine (70 mL × 3), dried over anhydrous Na_2SO_4, filtered and concentrated in vacuo. The crude product was purified by flash chromatography (hexane : EtOAc = 20:1) to afford **1** (0.58 g, 93% yield) as a white solid.

HRMS (ESI): m/z calcd for $C_{25}H_{24}O_5[M+H^+]$: 405.1697; found: 405.1636.

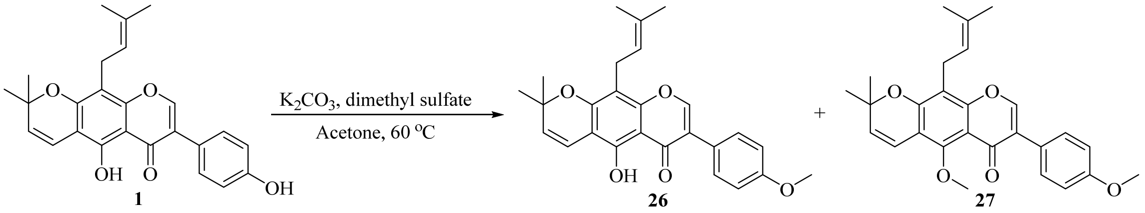

5-hydroxy-7-(4-methoxyphenyl)-2,2-dimethyl-10-(3-methylbut-2-en-1-yl)-2*H*,6*H*-pyrano[3,2-g]chromen-6-one 26: Compound **26** was synthesized by following a similar procedure as that of **9**.

^{1}H NMR (400 MHz, $CDCl_3$) δ 13.11 (s, 1H), 7.90 (s, 1H), 7.46 (d, *J* = 8.8 Hz, 2H), 6.98 (d, *J* = 8.8 Hz, 2H), 6.74 (d, *J* = 10.0 Hz, 1H), 5.62 (d, *J* = 10.0 Hz, 1H), 5.39-5.02 (m, 1H), 3.84 (s, 3H), 3.40 (d, *J* = 7.4 Hz, 2H), 1.82 (s, 3H), 1.69 (s, 3H), 1.47 (s, 6H).

^{13}C NMR (100 MHz, $CDCl_3$) δ 181.28, 159.75, 156.90, 154.99, 154.75, 152.51, 131.69, 130.14, 128.01, 123.23, 123.20, 122.03, 115.91, 114.11, 107.46, 105.95, 105.45, 77.81, 55.37, 28.23, 25.78, 21.30, 17.90.

HRMS (ESI): m/z calcd for $C_{25}H_{24}O_5[M+H^+]$: 419.1853; found: 419.1837.

5-methoxy-7-(4-methoxyphenyl)-2,2-dimethyl-10-(3-methylbut-2-en-1-yl)-2*H*,6*H*-pyrano[3,2-g]chromen-6-one 27: Compound **27** was synthesized by following a similar procedure as that of **9**.

^{1}H NMR (400 MHz, $CDCl_3$) δ 7.85 (s, 1H), 7.48 (d, *J* = 8.7 Hz, 2H), 6.96 (d, *J* = 8.7 Hz, 2H), 6.75 (d, *J* = 10.0 Hz, 1H), 5.72 (d, *J* = 10.0 Hz, 1H), 5.31 – 5.11 (m, 1H), 3.86 (s, 3H), 3.83 (s, 3H), 3.46 (d, *J* = 7.4 Hz, 2H), 1.83 (s, 3H), 1.69 (s, 3H), 1.47 (s, 6H).

^{13}C NMR (100 MHz, $CDCl_3$) δ 175.56, 159.47, 156.25, 155.21, 153.60, 150.42, 132.06, 130.58, 130.36, 125.24, 124.45, 121.58, 116.57, 113.92, 113.36, 113.06, 113.01, 77.44, 62.74, 55.37, 28.24, 25.82, 21.82, 17.96.

HRMS (ESI): m/z calcd for $C_{27}H_{28}O_5[M+H^+]$: 433.2010; found: 433.2015.

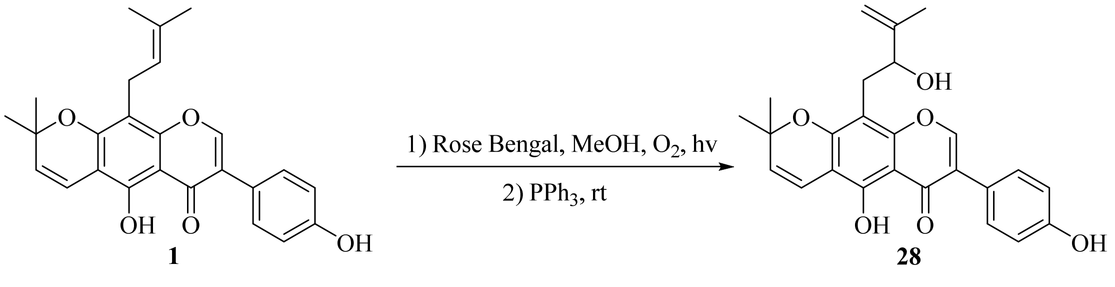

Dried air was continuously bubbled through a MeOH (20 mL) solution of **1** (0.75 g, 1.85 mmol) and Rose bengal (0.19 g, 0.19 mmol) as the photosensitizer. A 500 W halogen lamp was used as the

light source. The reaction mixture was irradiated and stirred at room temperature for 10 h. The crude residue was directly used without further purification. Triphenylphosphine (PPh_3) (0.73 g, 2.78 mmol) was added and the solution was stirred at room temperature for 1 h before concentrated in vacuo. The crude residue was purified by flash chromatography (hexane : EtOAc = 4:1) to afford **28** (0.51 g, 65%) as a white solid.

5-hydroxy-10-(2-hydroxy-3-methylbut-3-en-1-yl)-7-(4-hydroxyphenyl)-2,2-dimethyl-2*H*,6*H*-pyrano[3,2-g]chromen-6-one 28:

^{1}H NMR (400 MHz, $CDCl_3$) δ 13.18 (s, 1H), 7.89 (s, 1H), 7.41 (d, J = 8.4 Hz, 2H), 6.90 (d, J = 8.4 Hz, 2H), 6.75 (d, J = 10.0 Hz, 1H), 5.63 (d, J = 10.0 Hz, 1H), 5.03 (s, 1H), 4.96 (s, 1H), 4.84 (s, 1H), 4.33 (m, 1H), 3.04 (dd, J = 13.9, 4.6 Hz, 1H), 2.96 (dd, J = 13.9, 8.4 Hz, 1H), 1.88 (s, 3H), 1.50 (s, 6H).

^{13}C NMR (100 MHz, $CDCl_3$) δ 181.25, 157.26, 155.88, 155.64, 152.46, 149.12, 147.15, 130.36, 127.77, 123.31, 123.20, 115.79, 115.57, 110.79, 106.01, 105.29, 104.25, 78.46, 75.57, 29.05, 28.46, 28.34, 17.99.

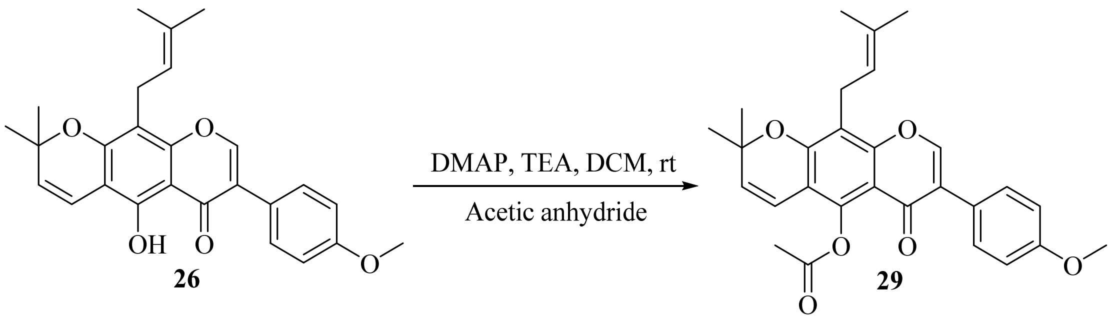

To a solution of **26** (0.63 g, 1.51 mmol) in anhydrous DCM (20 mL) was added TEA (0.13 mL, 0.91 mmol), DMAP (8.93 mg, 0.07 mmol) and acetic anhydride (43 μL, 0.45 mmol). The reaction mixture was stirred at room temperature for 4 h. H_2O was added to the reaction mixture. The aqueous layer was extracted three times with DCM (100 mL × 3). The combined organic layers were washed with brine (70 mL × 3), dried over anhydrous Na_2SO_4, filtered and concentrated in vacuo. The crude product was purified by flash chromatography (hexane : EtOAc = 50:1) to afford **29** (0.67 g, 96% yield) as a yellow solid.

7-(4-methoxyphenyl)-2,2-dimethyl-10-(3-methylbut-2-en-1-yl)-6-oxo-2*H*,6*H*-pyrano[3,2-g]chromen-5-yl acetate 29:

^{1}H NMR (400 MHz, $CDCl_3$) δ 7.83 (s, 1H), 7.40 (d, *J* = 8.8 Hz, 2H), 6.94 (d, *J* = 8.8 Hz, 2H), 6.49 (d, *J* = 10.1 Hz, 1H), 5.76 (d, *J* = 10.1 Hz, 1H), 5.24 – 5.12 (m, 1H), 3.82 (s, 3H), 3.47 (d, *J* = 7.3 Hz, 2H), 2.44 (s, 3H), 1.82 (s, 3H), 1.69 (s, 3H), 1.47 (s, 6H).

^{13}C NMR (100 MHz, $CDCl_3$) δ 175.26, 169.67, 159.58, 155.83, 154.81, 150.88, 142.86, 132.24, 131.81, 130.38, 125.31, 124.16, 121.20, 115.62, 115.08, 113.99, 112.62, 111.54, 77.24, 55.34, 28.27, 25.76, 21.89, 21.15, 17.94.

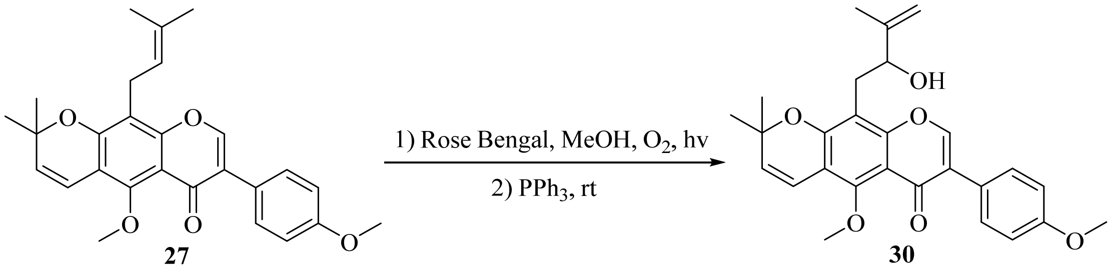

10-(2-hydroxy-3-methylbut-3-en-1-yl)-5-methoxy-7-(4-methoxyphenyl)-2,2-dimethyl-2H,6H-pyrano[3,2-g]chromen-6-one 30: Compound **30** was synthesized by following a similar procedure as that of **28**.

^{1}H NMR (400 MHz, $CDCl_3$) δ 7.85 (s, 1H), 7.47 (d, *J* = 8.7 Hz, 2H), 6.96 (d, *J* = 8.7 Hz, 2H), 6.77 (d, *J* = 10.1 Hz, 1H), 5.73 (d, *J* = 10.1 Hz, 1H), 4.97 (s, 1H), 4.85 (s, 1H), 4.35 (dd, *J* = 8.4, 4.5 Hz, 1H), 3.88 (s, 3H), 3.84 (s, 3H), 3.11 (dd, *J* = 13.7, 4.5 Hz, 1H), 3.10 (dd, *J* = 13.7, 8.4 Hz, 1H), 1.89 (s, 3H), 1.50 (s, 3H), 1.49 (s, 3H).

HRMS (ESI): m/z calcd for $C_{27}H_{28}O_6[M+H^+]$: 449.1959; found: 449.1965.

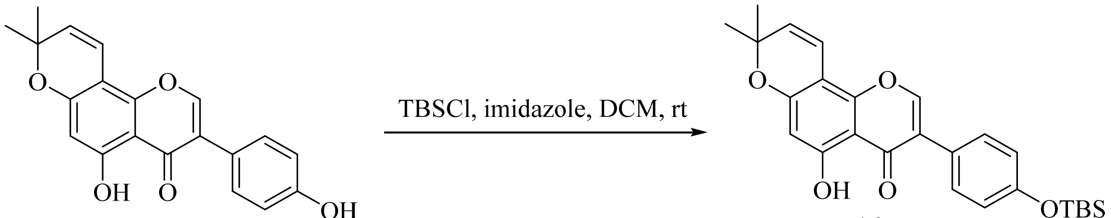

3-(4-((tert-butyldimethylsilyl)oxy)phenyl)-5-hydroxy-8,8-dimethyl-4*H*,8*H*-pyrano[2,3-f]chromen-4-one 16: Compound **16** was synthesized by following a similar procedure as that of **23**.

^{1}H NMR (400 MHz, $CDCl_3$) δ 12.95 (s, 1H), 7.89 (s, 1H), 7.49 – 7.34 (d, *J* = 8.6 Hz, 2H), 6.91 (d, *J* = 8.6 Hz, 2H), 6.68 (d, *J* = 10.0 Hz, 1H), 6.29 (s, 1H), 5.59 (d, *J* = 10.0 Hz, 1H), 1.47 (s, 6H), 1.00 (s, 9H), 0.22 (s, 6H).

^{13}C NMR (100 MHz, $CDCl_3$) δ 180.95, 162.31, 159.58, 156.10, 152.45, 152.28, 130.07, 127.47, 123.71, 123.50, 120.25, 114.62, 106.08, 101.13, 100.36, 78.11, 28.23, 25.69, 18.24, -4.36.

HRMS (ESI): m/z calcd for $C_{26}H_{30}O_5Si[M+H^+]$: 451.1935; found: 451.1950.

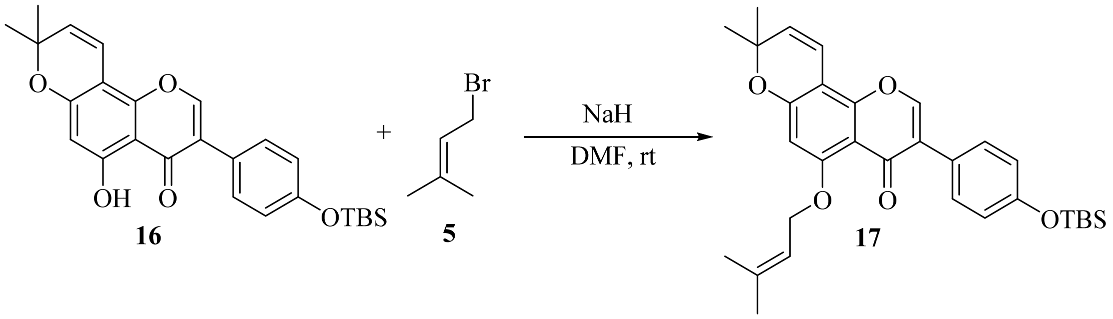

3-(4-((tert-butyldimethylsilyl)oxy)phenyl)-8,8-dimethyl-5-((3-methylbut-2-en-1-yl)oxy)-4*H*,8*H*-pyrano[2,3-f]chromen-4-one 17: Compound **17** was synthesized by following a similar procedure as that of **24**.

^{1}H NMR (400 MHz, $CDCl_3$) δ 7.75 (s, 1H), 7.39 (d, J = 8.4 Hz, 2H), 6.85 (d, J = 8.4 Hz, 2H), 6.72 (d, J = 10.0 Hz, 1H), 6.31 (s, 1H), 5.73 – 5.43 (m, 2H), 4.61 (d, J = 6.4 Hz, 2H), 1.76 (s, 3H), 1.72 (s, 3H), 1.48 (s, 6H), 0.98 (s, 9H), 0.20 (s, 6H).

^{13}C NMR (100 MHz, $CDCl_3$) δ 175.44, 160.53, 157.51, 155.57, 154.14, 149.75, 137.46, 130.43, 127.25, 125.93, 125.06, 119.88, 119.38, 115.19, 109.79, 102.27, 97.59, 77.97, 66.46, 28.23, 25.81, 25.72, 18.40, 18.23, -4.36.

HRMS (ESI): m/z calcd for $C_{26}H_{30}O_5Si[M+H^+]$: 519.2561; found: 519.2554.

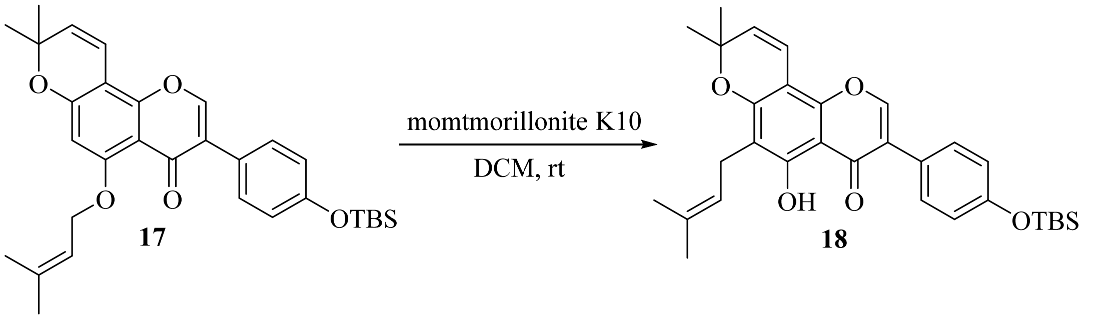

To a solution of compound **17** (2.5 g, 4.82 mmol) in dry DCM (30 mL) was added montmorillonite K10 (2.5 g, 1 wt) under argon. After stirring for 1 h at at room temperature, the reaction mixture was filtered and concentrated. The crude product was purified by flash chromatography (hexane : EtOAc = 600:1) to afford **18** (2.23 g, 89% yield) as a yellow solid.

3-(4-((tert-butyldimethylsilyl)oxy)phenyl)-5-hydroxy-8,8-dimethyl-6-(3-methylbut-2-en-1-yl)-4*H*,8*H*-pyrano[2,3-f]chromen-4-one 18:

^{1}H NMR (400 MHz, $CDCl_3$) δ 13.17 (s, 1H), 7.87 (s, 1H), 7.39 (d, J = 8.7 Hz, 2H), 6.91 (d, J = 8.7 Hz, 2H), 6.70 (d, J = 10.0 Hz, 1H), 5.59 (d, J = 10.0 Hz, 1H), 5.33 – 5.09 (m, 1H), 3.35 (d, J = 7.3 Hz, 2H), 1.81 (s, 3H), 1.68 (s, 3H), 1.48 (s, 6H), 1.00 (s, 9H), 0.23 (s, 6H).

^{13}C NMR (100 MHz, $CDCl_3$) δ 180.97, 159.40, 157.21, 155.98, 152.29, 150.52, 131.63, 130.08, 127.14, 123.81, 123.49, 121.98, 120.22, 115.02, 112.83, 105.63, 100.73, 77.86, 28.15, 25.84, 25.70, 21.32, 18.24, 17.96, -4.35.

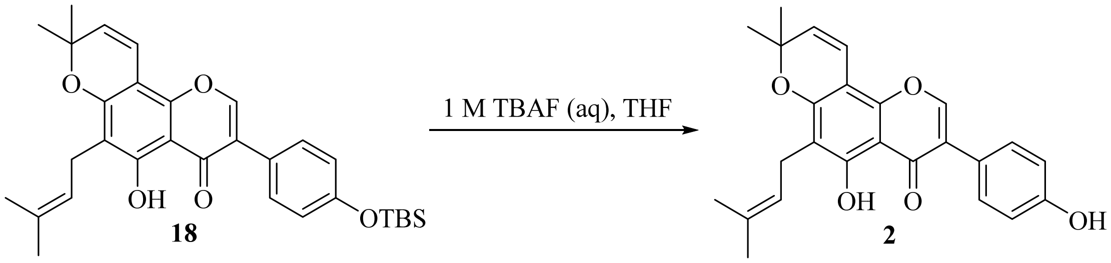

5-hydroxy-3-(4-hydroxyphenyl)-8,8-dimethyl-6-(3-methylbut-2-en-1-yl)-4*H*,8*H*-pyrano[2,3-f]chromen-4-one 2: Compound **2** was synthesized by following a similar procedure as that of **1**. HRMS (ESI): m/z calcd for $C_{25}H_{24}O_5[M+H^+]$: 405.1697; found: 405.1703.

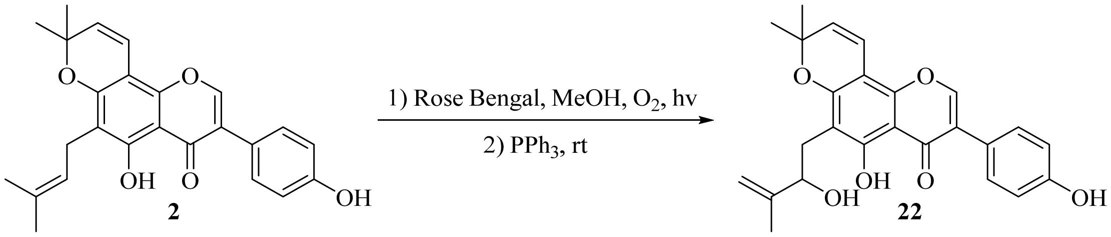

5-hydroxy-6-(2-hydroxy-3-methylbut-3-en-1-yl)-3-(4-hydroxyphenyl)-8,8-dimethyl-4*H*,8*H*-pyrano[2,3-f]chromen-4-one 22: Compound **22** was synthesized by following a similar procedure as that of **28**.

^{1}H NMR (400 MHz, $CDCl_3$) δ 13.42 (s, 1H), 7.86 (s, 1H), 7.35 (d, J = 6.9 Hz, 2H), 6.96 – 6.79 (m, 2H), 6.71 (d, J = 10.0 Hz, 1H), 5.60 (d, J = 10.0 Hz, 1H), 5.03 (s, 1H), 4.85 (t, J = 1.7 Hz, 1H), 4.31 (dd, J = 9.1, 3.5 Hz, 1H), 3.04 (dd, J = 13.9, 3.5 Hz, 1H), 2.91 (dd, J = 13.9, 9.1 Hz, 1H), 1.88 (s, 3H), 1.51 (s, 3H), 1.50 (s, 3H).

^{13}C NMR (100 MHz, $CDCl_3$) δ 181.01, 159.97, 157.32, 156.13, 152.46, 150.88, 147.44, 130.24, 127.10, 123.52, 122.84, 115.57, 114.86, 110.28, 109.88, 105.58, 100.87, 78.47, 76.01, 29.04, 28.43, 28.22, 18.24.

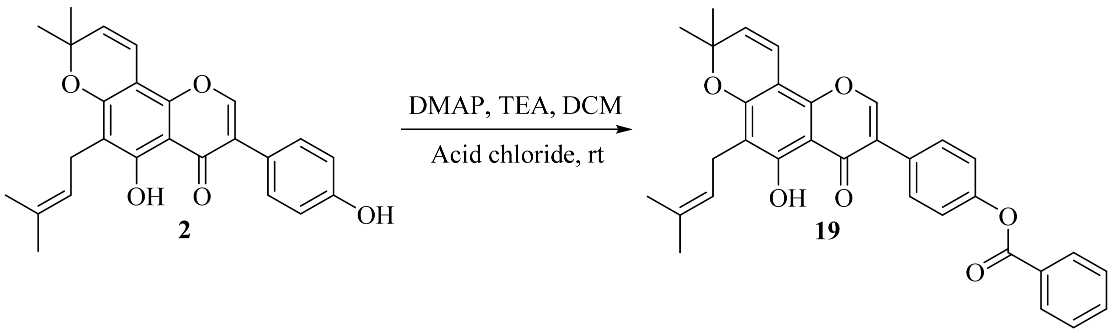

4-(5-hydroxy-8,8-dimethyl-6-(3-methylbut-2-en-1-yl)-4-oxo-4*H*,8*H*-pyrano[2,3-f]chromen-3-yl)phenyl benzoate 19: Compound **19** was synthesized by following a similar procedure as that of **29**.

^{1}H NMR (400 MHz, $CDCl_3$) δ 13.09 (s, 1H), 8.23 (dd, *J* = 8.5, 1.4 Hz, 2H), 7.93 (s, 1H), 7.70 – 7.42 (m, 5H), 7.31 (d, *J* = 8.5 Hz, 2H), 6.71 (d, *J* = 10.0 Hz, 1H), 5.60 (d, *J* = 10.0 Hz, 1H), 5.33 – 5.17 (m, 1H), 3.36 (d, *J* = 7.4 Hz, 2H), 1.82 (s, 3H), 1.69 (s, 3H), 1.49 (s, 6H).

^{13}C NMR (100 MHz, $CDCl_3$) δ 180.64, 165.15, 159.40, 157.37, 152.78, 151.06, 150.49, 133.72, 131.67, 130.26, 130.15, 129.45, 128.75, 128.63, 127.30, 123.06, 121.96, 121.93, 114.95, 113.05, 105.61, 100.83, 77.94, 28.17, 25.83, 21.33, 17.95.

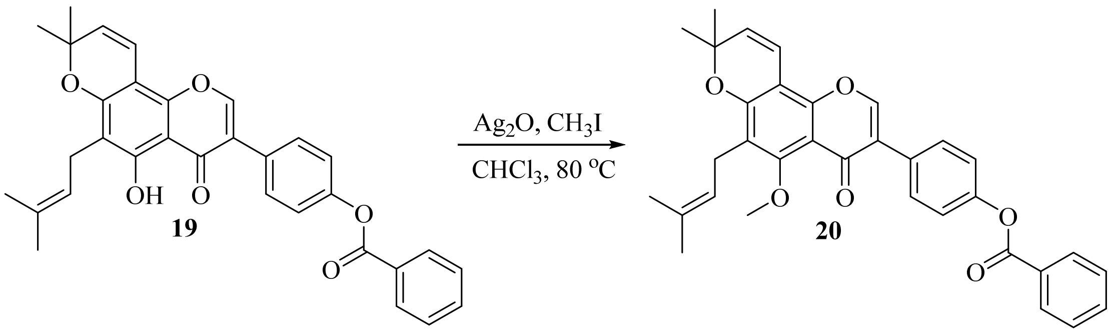

A mixture of compound **19** (0.5 g, 0.98 mmol), Ag_2O (1.14 g, 4.93 mmol), and idomethane (1.23 mL, 19.75 mmol) in dry chloroform was stirred at 80 °C before concentrated in vacue. The crude product was purified by flash chromatography (hexane : EtOAc = 7:1) to afford **20** (0.48 g, 79% yield) as a yellow solid.

4-(5-methoxy-8,8-dimethyl-6-(3-methylbut-2-en-1-yl)-4-oxo-4*H*,8*H*-pyrano[2,3-f]chromen-3-yl)phenyl benzoate 20:

^{1}H NMR (400 MHz, $CDCl_3$) δ 8.42 – 8.09 (m, 2H), 7.88 (s, 1H), 7.69 – 7.56 (m, 3H), 7.52 (t, *J* = 7.7 Hz, 2H), 7.33 – 7.12 (m, 2H), 6.78 (d, *J* = 10.0 Hz, 1H), 5.67 (d, *J* = 10.0 Hz, 1H), 5.29 – 5.06 (m, 1H), 3.87 (s, 3H), 3.39 (d, *J* = 7.2 Hz, 2H), 1.81 (s, 3H), 1.68 (s, 3H), 1.49 (s, 6H).

^{13}C NMR (100 MHz, $CDCl_3$) δ 174.83, 165.19, 158.15, 155.95, 152.10, 150.81, 150.70, 133.79, 131.50, 130.39, 130.25, 129.93, 129.57, 128.94, 128.60, 125.20, 122.58, 121.93, 121.74, 115.21, 112.78, 105.88, 77.84, 62.44, 28.15, 25.80, 22.27, 18.01.

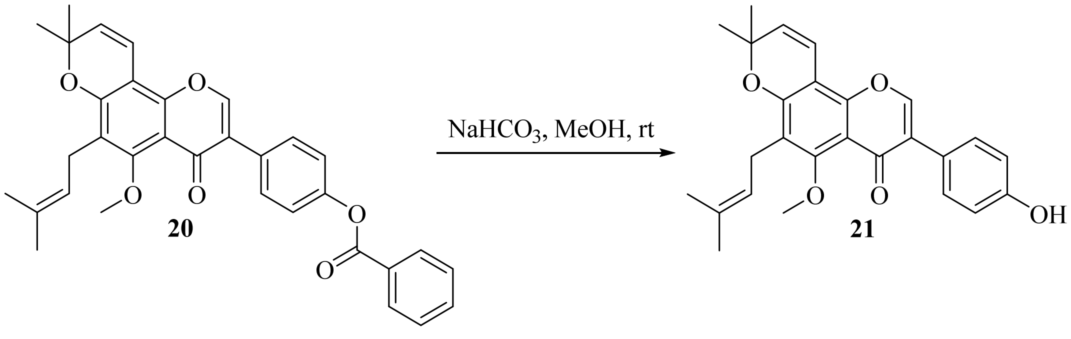

To a solution of compound **20** (1.2 g, 2.30 mmol) in methanol (15 ml) was added $NaHCO_3$ (0.14 g, 1.65 mmol) at room temperature for 2 h. Then, the mixture was acidized by 1 N HCl soulution and extracted by DCM (80 ml × 3). the organic layers were washed with brine (30 mL × 3), dried over anhydrous Na_2SO_4, filtered and concentrated in vacuo. The crude product was purified by flash chromatography (hexane : EtOAc = 10:1) to afford **21** (0.85 g, 87% yield) as a yellow solid.

3-(4-hydroxyphenyl)-5-methoxy-8,8-dimethyl-6-(3-methylbut-2-en-1-yl)-4*H*,8*H*-pyrano[2,3-f]chromen-4-one 21:

^1H NMR (400 MHz, $CDCl_3$) δ 7.82 (s, 1H), 7.34 (d, J = 8.4 Hz, 2H), 6.84 (d, J = 8.4 Hz, 2H), 6.77 (d, J = 10.0 Hz, 1H), 5.65 (d, J = 10.0 Hz, 1H), 5.17 (t, J = 7.2 Hz, 1H), 3.86 (s, 3H), 3.39 (d, J = 7.2 Hz, 2H), 1.81 (s, 3H), 1.67 (s, 3H), 1.48 (s, 6H).

HRMS (ESI): m/z calcd for $C_{26}H_{26}O_5[M+H^+]$: 419.1853; found: 419.1837.

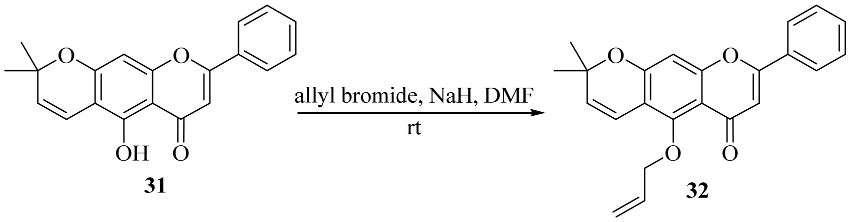

To a solution of **31**(1.70 g, 5.31 mmol) in anhydrous DMF (50 mL) was added NaH (0.64 g, 15.92 mmol) and allyl bromide (0.97 mL, 10.61 mmol). The reaction mixture was stirred at room temperature for 3 h. The reaction mixture was quenched by brine (50 mL). The aqueous layer was extracted three times with EtOAc (50 mL × 3). The combined organic layers were washed with brine (30 mL × 3), dried over anhydrous Na_2SO_4, filtered and concentrated in vacuo. The crude product was purified by flash chromatography (hexane : EtOAc = 8:1) to afford **32** (1.46 g, 76% yield) as a

yellow solid.

5-(allyloxy)-2,2-dimethyl-8-phenyl-2*H*,6*H*-pyrano[3,2-g]chromen-6-one 32:

^{1}H NMR (400 MHz, $CDCl_3$) δ 7.95 – 7.78 (m, 2H), 7.55 – 7.45 (m, 3H), 6.78 – 6.69 (m, 2H), 6.67 – 6.59 (m, 1H), 6.25 – 6.08 (m, 1H), 5.70 (d, *J* = 10.1 Hz, 1H), 5.39 (d, *J* = 17.2 Hz, 1H), 5.26 (d, *J* = 10.1 Hz, 1H), 4.73 – 4.47 (m, 2H), 1.47 (s, 3H), 1.47 (s, 3H).

^{13}C NMR (100 MHz, $CDCl_3$) δ 177.09, 161.01, 158.66, 158.16, 153.79, 133.95, 131.54, 131.28, 130.62, 128.98, 125.99, 118.30, 116.65, 113.66, 112.67, 108.45, 100.95, 77.71, 77.43, 28.27.

HRMS (ESI): m/z calcd for $C_{23}H_{20}O_4$ [M+H$^+$]: 361.1434; found: 361.1431.

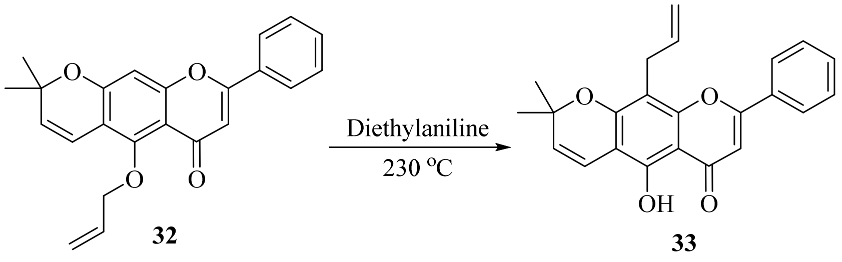

A solution of **32** (300 mg, 0.83 mmol) in diethylaniline (20 mL) was stirred at 230 ºC for 0.5 h. The resulting mixture was cooled to room temperature and EtOAc was added to the reaction mixture. The organic layers was extracted three times with 1N HCl solution (100 mL × 3) and the organic layers were washed with brine (30 mL × 3), dried over anhydrous Na_2SO_4, filtered and concentrated in vacuo. The crude product was purified by flash chromatography (hexane : EtOAc = 20:1) to afford **33** (0.26 g, 85% yield) as a yellow solid.

10-allyl-5-hydroxy-2,2-dimethyl-8-phenyl-2*H*,6*H*-pyrano[3,2-g]chromen-6-one 33:

^{1}H NMR (400 MHz, $CDCl_3$) δ 7.96 – 7.81 (m, 2H), 7.64 – 7.44 (m, 3H), 6.74 (d, *J* = 10.0 Hz, 1H), 6.65 (s, 1H), 6.08 – 5.90 (m, 1H), 5.63 (d, *J* = 10.0 Hz, 1H), 5.09 (d, *J* = 17.1 Hz, 1H), 5.02 (d, *J* = 10.0 Hz, 1H), 3.57 (d, *J* = 6.0 Hz, 2H), 1.47 (s, 6H).

^{13}C NMR (100 MHz, $CDCl_3$) δ 182.91, 163.54, 157.21, 154.78, 154.63, 135.89, 131.73, 131.68, 129.15, 128.06, 126.21, 115.79, 114.98, 105.69, 105.54,105.48, 105.36, 77.96, 28.30, 26.73.

HRMS (ESI): m/z calcd for $C_{23}H_{20}O_4$ [M+H$^+$]: 361.1434; found: 361.1425.

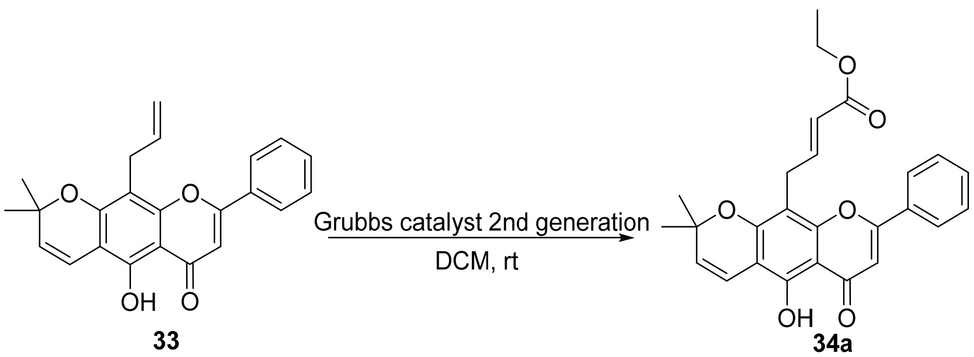

33 **34a**

To a solution of **33** (50 mg, 0.14 mmol) in anhydrous DCM (20 mL) was added Grubbs catalyst 2nd generation (23 mg, 0.03 mmol) and methyl acrylate (0.18mL, 0.21mmol). The reaction mixture was stirred at room temperature for 5 h. The reaction mixture was concentrated in vacuo. The crude product was purified by flash chromatography (hexane : EtOAc = 8:1) to afford **34a** (49.92 mg, 86% yield) as a yellow solid.

Ethyl(*E*)-4-(5-hydroxy-2,2-dimethyl-6-oxo-8-phenyl-2*H*,6*H*-pyrano[3,2 g]chromen-10-yl)but-2-enoate 34a:

^{1}H NMR (400 MHz, $CDCl_3$) δ 13.02 (s, 1H),δ 7.96 – 7.73 (m, 2H), 7.53 (d, *J* = 7.2 Hz, 3H), 7.11 (dt, *J* = 15.6, 6.2 Hz, 1H), 6.73 (d, *J* = 10.0 Hz, 1H), 6.65 (s, 1H), 5.82 (dd, *J* = 15.6, 1.7 Hz, 1H), 5.63 (d, *J* = 10.0 Hz, 1H), 4.14 (q, *J* = 7.1 Hz, 2H), 3.70 (dd, *J* = 6.2, 1.7 Hz, 2H), 1.46 (s, 6H), 1.24 (t, *J* = 7.1 Hz, 3H).

^{13}C NMR (100 MHz, $CDCl_3$) δ 182.76, 166.65, 163.63, 157.28, 155.31, 154.60, 146.09, 131.84, 131.52, 129.23, 128.08, 126.19, 121.76, 115.63, 105.78, 105.53, 105.39, 103.35, 78.34, 60.28, 28.35, 25.29, 14.24.

HRMS (ESI): m/z calcd for $C_{26}H_{24}O_6$ [M+H$^+$]: 433.1646; found: 433.1652.

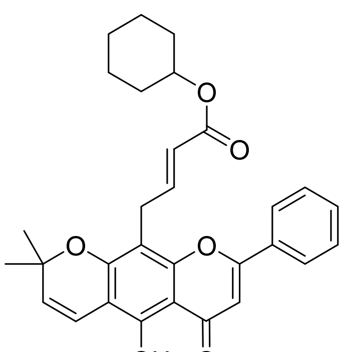

34b

cyclohexyl(*E*)-4-(5-hydroxy-2,2-dimethyl-6-oxo-8-phenyl-2*H*,6*H*-pyrano[3,2-g]chromen-10-yl)but-2-enoate 34b:

^{1}H NMR (400 MHz, $CDCl_3$) δ 13.03 (s, 1H), 7.83 (dt, J = 4.5, 2.1 Hz, 2H), 7.61 – 7.41 (m, 3H), 7.09 (dtd, J = 15.6, 6.3, 2.6 Hz, 1H), 6.74 (d, J = 10.0 Hz, 1H), 6.65 (s, 1H), 5.82 (d, J = 15.6 Hz, 1H), 5.64 (d, J = 10.0 Hz, 1H), 4.77 (tt, J = 8.6, 3.5 Hz, 1H), 3.69 (dt, J = 6.3, 2.6 Hz, 2H), 1.91 – 1.76 (m, 2H), 1.75 – 1.60 (m, 2H), 1.56 – 1.11 (m, 12H).

^{13}C NMR (100 MHz, $CDCl_3$) δ 182.79, 166.12, 163.65, 157.30, 155.29, 154.61, 145.73, 131.84, 131.54, 129.23, 128.09, 126.22, 122.33, 115.63, 105.79, 105.53, 105.40, 103.46, 78.34, 72.51, 31.65, 28.35, 25.39, 25.29, 23.74.

HRMS (ESI): m/z calcd for $C_{30}H_{30}O_6$ [M+H$^+$]: 487.2115; found: 487.2115.

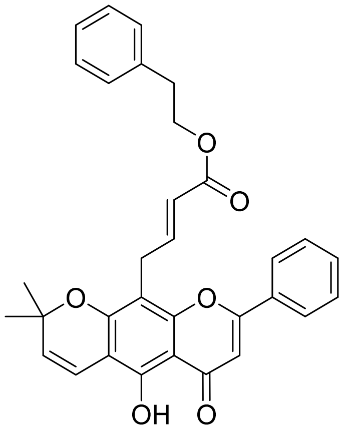

34c

^{1}H NMR (400 MHz, $CDCl_3$) δ 13.05 (s, 1H), 7.93 – 7.76 (m, 2H), 7.54 (q, J = 2.3 Hz, 3H), 7.29 (d, J = 2.9 Hz, 2H), 7.18 (dtd, J = 15.6, 6.2, 2.3 Hz, 1H), 7.00 – 6.91 (m, 1H), 6.89 (dd, J = 8.4, 2.3 Hz, 2H), 6.76 (dd, J = 10.1, 2.3 Hz, 1H), 6.73 – 6.57 (m, 1H), 5.91 (dd, J = 15.6, 1.7 Hz, 1H), 5.65 (dd, J = 10.1, 2.3 Hz, 1H), 4.63 – 4.34 (m, 2H), 4.18 (dt, J = 4.4, 2.6 Hz, 2H), 3.74 (dt, J = 6.2, 1.7 Hz, 2H), 1.48 (s, 6H).

^{13}C NMR (100 MHz, $CDCl_3$) δ 182.76, 166.49, 163.63, 158.46, 157.28, 155.35, 154.60, 147.08, 131.85, 131.49, 129.51, 129.24, 128.08, 126.18, 121.24, 121.17, 115.62, 114.58, 105.80, 105.55, 105.40, 103.17, 78.38, 65.88, 62.75, 28.35, 25.37.

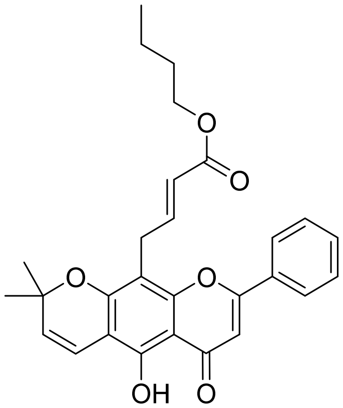

34d

butyl(E)-4-(5-hydroxy-2,2-dimethyl-6-oxo-8-phenyl-2H,6H-pyrano[3,2-g]chromen-10-yl)but-2-enoate 34d:

^{1}H NMR (400 MHz, $CDCl_3$) δ 13.02 (s, 1H), 7.83 (dt, *J* = 8.0, 1.6 Hz, 2H), 7.65 – 7.46 (m, 3H), 7.11 (dt, *J* = 15.6, 6.2 Hz, 1H), 6.74 (d, *J* = 10.0 Hz, 1H), 6.66 (s, 1H), 5.83 (d, *J* = 15.6 Hz, 1H), 5.64 (d, *J* = 10.0 Hz, 1H), 4.09 (t, *J* = 6.7 Hz, 2H), 3.70 (d, *J* = 6.2 Hz, 2H), 1.65 – 1.54 (m, 2H), 1.47 (s, 6H), 1.42 – 1.27 (m, 2H), 0.90 (t, *J* = 7.4 Hz, 3H).

^{13}C NMR (100 MHz, $CDCl_3$) δ 182.78, 166.77, 163.64, 157.29, 155.30, 154.60, 146.06, 131.86, 131.51, 129.24, 128.09, 126.20, 121.77, 115.63, 105.78, 105.53, 105.40, 103.36, 78.35, 64.23, 30.68, 28.35, 25.31, 19.14, 13.72.

HRMS (ESI): m/z calcd for $C_{28}H_{28}O_6$ [M+H$^+$]: 461.1959; found: 461.1979.

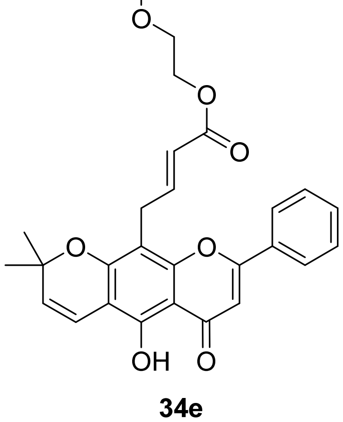

34e

2-methoxyethyl(E)-4-(5-hydroxy-2,2-dimethyl-6-oxo-8-phenyl-2H,6H-pyrano[3,2-g]chromen-10-yl)but-2-enoate 34e:

^{1}H NMR (400 MHz, $CDCl_3$) δ 13.02 (s, 1H), 7.97 – 7.68 (m, 2H), 7.53 (d, *J* = 7.0 Hz, 3H), 7.16 (dt, *J* = 15.6, 6.1 Hz, 1H), 6.73 (d, *J* = 10.0 Hz, 1H), 6.65 (s, 1H), 5.85 (d, *J* = 15.6 Hz, 1H), 5.63 (d, *J*

= 10.0 Hz, 1H), 4.40 – 4.14 (m, 2H), 3.70 (d, *J* = 6.1 Hz, 2H), 3.57 (dd, *J* = 5.7, 3.6 Hz, 2H), 3.34 (s, 3H), 1.46 (s, 6H).

^{13}C NMR (100 MHz, $CDCl_3$) δ 182.76, 166.61, 163.63, 157.28, 155.32, 154.59, 146.86, 131.85, 131.49, 129.24, 128.09, 126.20, 121.26, 115.62, 105.78, 105.53, 105.38, 103.22, 78.35, 77.26, 70.49, 63.42, 59.02, 28.36, 25.31.

HRMS (ESI): m/z calcd for $C_{28}H_{28}O_6$ [M+H$^+$]: 463.1751; found:463.1759.

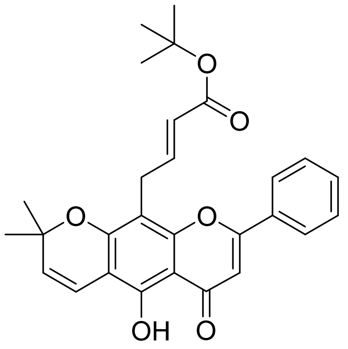

34f

tert-butyl(E)-4-(5-hydroxy-2,2-dimethyl-6-oxo-8-phenyl-2H,6H-pyrano[3,2-g]chromen-10-yl)but-2-enoate 34f:

^{1}H NMR (400 MHz, $CDCl_3$) δ 13.03 (s, 1H), 7.89 – 7.77 (m, 2H), 7.54 (d, *J* = 6.9 Hz, 3H), 7.00 (dt, *J* = 15.4, 6.3 Hz, 1H), 6.74 (d, *J* = 10.0 Hz, 1H), 6.66 (s, 1H), 5.76 (d, *J* = 15.6 Hz, 1H), 5.64 (d, *J* = 10.0 Hz, 1H), 3.67 (d, *J* = 6.3 Hz, 2H), 1.47 (s, 6H), 1.44 (s, 9H).

^{13}C NMR (100 MHz, $CDCl_3$) δ 182.81, 166.05, 163.67, 157.28, 155.25, 154.62, 144.82, 131.84, 131.56, 129.23, 128.10, 126.24, 123.52, 115.65, 105.79, 105.52, 105.40, 103.64, 80.26, 78.32, 28.33, 28.13, 25.18.

HRMS (ESI): m/z calcd for $C_{28}H_{28}O_6$ [M+H$^+$]: 461.1959; found: 461.1943.

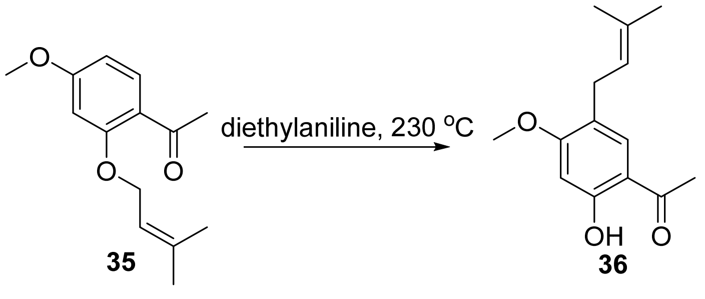

1-(2-hydroxy-4-methoxy-5-(3-methylbut-2-en-1-yl)phenyl)ethan-1-one: Compound **36** was synthesized by following a similar procedure as that of **33.**

^{1}H NMR (400 MHz, $CDCl_3$) δ 12.64 (s, 1H), 7.32 (s, 1H), 6.31 (s, 1H), 5.39 – 4.99 (m, 1H), 3.78 (s, 3H), 3.14 (d, J = 7.3 Hz, 2H), 2.46 (s, 3H), 1.68 (s, 3H), 1.63 (s, 3H).

^{13}C NMR (100 MHz, $CDCl_3$ δ 202.59, 164.07, 133.10, 130.74, 122.02, 121.67, 113.15, 99.04, 55.70, 27.80, 26.23, 25.81, 17.80.

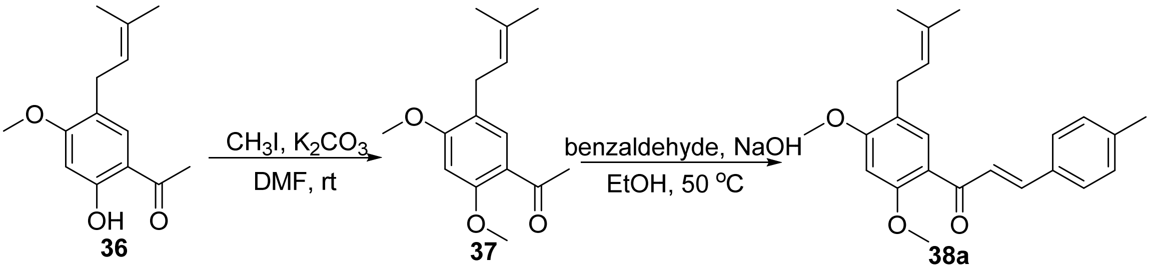

To a solution of **36** (0.50 g, 2.13 mmol) in anhydrous DMF (50 mL) was added K_2CO_3 (0.44 g, 3.20 mmol) and CH_3I (0.17 mL, 2.78 mmol). The reaction mixture was stirred at room temperature for 2 h. The reaction mixture was quenched by brine (50 mL). The aqueous layer was extracted three times with EtOAc (250 mL × 3). The combined organic layers were washed with brine (100 mL × 3), dried over anhydrous Na_2SO_4, filtered and concentrated in vacuo to afford **37** as a yellow solid. To a solution of compound **37** and p-methyl benzaldehyde (3.84 mL, 3.20 mmol) in EtOH (250 mL) was added NaOH (1.28 g, 3.20 mmol) at 0 ºC. After stirring for 0.5 h, the resulting solution was stirred at 50 °C for 24 h before the addition of water and EtOAc. The aqueous phase was extracted three times with EtOAc (200 mL × 3), and the organic layers were successively washed three times with brine (100 mL × 3), dried over anhydrous Na_2SO_4, filtered and concentrated in vacuo. The crude product was purified by flash chromatography (hexane : EtOAc = 15:1) to afford **38a** (0.66 g, 89% yield) as a yellow solid.

(E)-1-(2,4-dimethoxy-5-(3-methylbut-2-en-1-yl)phenyl)-3-(p-tolyl)prop-2-en-1-one 38a

^{1}H NMR (400 MHz, $CDCl_3$) δ 7.65 (dd, J = 15.9, 3.0 Hz, 1H), 7.56 (d, J = 3.0 Hz, 1H), 7.53 – 7.40 (m, 3H), 7.19 (dd, J = 8.1, 2.9 Hz, 2H), 6.45 (d, J = 3.0 Hz, 1H), 5.28 (q, J = 7.7, 5.4 Hz, 1H), 4.07 – 3.74 (m, 6H), 3.26 (dd, J = 7.7, 2.8 Hz, 2H), 2.38 (s, 3H), 1.71 (s, 3H), 1.70 (s, 3H).

^{13}C NMR (100 MHz, $CDCl_3$) δ 190.84, 161.59, 159.04, 141.87, 140.27, 132.87, 132.63, 131.93, 129.58, 128.31, 126.49, 122.79, 122.25, 121.30, 94.98, 56.15, 55.60, 27.72, 25.84, 21.51, 17.79.

HRMS (ESI): m/z calcd for $C_{23}H_{26}O_3$ [$M+H^+$]: 351.1955; found: 351.1941.

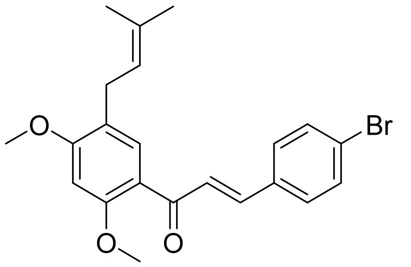

38b

(E)-3-(4-bromophenyl)-1-(2,4-dimethoxy-5-(3-methylbut-2-en-1-yl)phenyl)prop-2-en-1-one 38b:

^{1}H NMR (400 MHz, $CDCl_3$) δ 7.81 – 7.32 (m, 7H), 6.45 (s, 1H), 5.35 – 5.12 (m, 1H), 3.93 (s, 3H), 3.91 (s, 3H), 3.26 (d, J = 7.5 Hz, 2H), 1.72 (s, 3H), 1.70 (s, 3H).

^{13}C NMR (100 MHz, $CDCl_3$) δ 190.17, 161.92, 159.24, 140.05, 134.63, 132.67, 132.04, 132.01, 129.63, 128.03, 123.93, 122.98, 122.19, 120.93, 94.90, 56.11, 55.60, 27.70, 25.81, 17.77.

HRMS (ESI): m/z calcd for $C_{22}H_{23}O_3Br$ [M+H$^+$]: 415.0903; found: 415.0902.

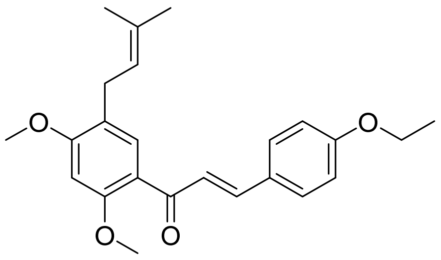

38c

(E)-1-(2,4-dimethoxy-5-(3-methylbut-2-en-1-yl)phenyl)-3-(4-ethoxyphenyl)prop-2-en-1-one 38c:

^{1}H NMR (400 MHz, $CDCl_3$) δ 7.52 (d, J = 15.7 Hz, 1H), 7.47 – 7.35 (m, 3H), 7.28 (d, J = 15.7 Hz, 1H), 6.77 (d, J = 8.5 Hz, 2H), 6.33 (s, 1H), 5.16 (t, J = 7.5 Hz, 1H), 3.94 (q, J = 7.0 Hz, 2H), 3.79 (s, 3H), 3.78 (s, 3H), 3.14 (d, J = 7.5 Hz, 2H), 1.59 (d, J = 7.7 Hz, 6H), 1.30 (t, J = 7.0 Hz, 3H).

^{13}C NMR (100 MHz, $CDCl_3$) δ 190.80, 161.46, 160.61, 158.93, 141.81, 132.55, 131.86, 129.95, 128.14, 125.13, 122.74, 122.31, 121.46, 114.79, 95.05, 63.60, 56.15, 55.58, 27.72, 25.81, 17.77, 14.76.

HRMS (ESI): m/z calcd for $C_{24}H_{28}O_4$ [M+H$^+$]: 381.2060; found: 381.2058.

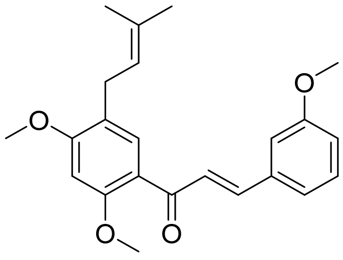

38d

(E)-1-(2,4-dimethoxy-5-(3-methylbut-2-en-1-yl)phenyl)-3-(3methoxyphenyl)prop-2-en-1-one 38d:

^{1}H NMR (400 MHz, $CDCl_3$) δ 7.69 – 7.45 (m, 3H), 7.31 (t, J = 7.9 Hz, 1H), 7.19 (d, J = 7.7 Hz, 1H), 7.12 (s, 1H), 6.92 (dd, J = 8.0, 2.6 Hz, 1H), 6.45 (s, 1H), 5.28 (t, J = 8.4 Hz, 1H), 3.93 (s, 3H), 3.91 (s, 3H), 3.84 (s, 3H), 3.26 (d, J = 8.4 Hz, 2H), 1.72 (s, 3H), 1.70 (s, 3H).

^{13}C NMR (100 MHz, $CDCl_3$) δ 190.64, 161.73, 160.55, 159.88, 159.14, 141.56, 137.08, 132.65, 131.96, 129.79, 127.78, 122.88, 122.22, 120.93, 115.50, 113.44, 94.97, 56.13, 55.59, 55.30, 27.70, 25.81, 17.77.

HRMS (ESI): m/z calcd for $C_{23}H_{26}O_4$ [M+H$^+$]: 367.1904; found: 367.1893.

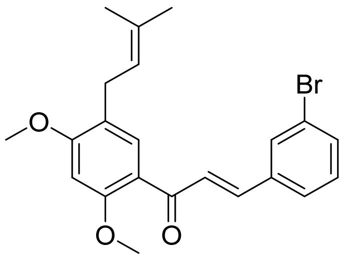

38e

(E)-3-(3-bromophenyl)-1-(2,4-dimethoxy-5-(3-methylbut-2-en-1-yl)phenyl)prop-2-en-1-one 38e:

^{1}H NMR (400 MHz, $CDCl_3$) δ 7.59 – 7.40 (m, 5H), 7.39 – 7.32 (m, 1H), 7.18 (d, J = 3.7 Hz, 1H), 6.37 (d, J = 3.5 Hz, 1H), 5.21 (t, J = 8.0 Hz, 1H), 3.84 (dd, J = 7.1, 3.6 Hz, 6H), 3.18 (d, J = 8.0 Hz, 2H), 1.65 (s, 3H), 1.63 (s, 3H).

^{13}C NMR (100 MHz, $CDCl_3$) δ 190.21, 161.93, 159.24, 140.08, 134.62, 132.71, 132.06, 132.03, 129.65, 128.00, 123.95, 122.96, 122.17, 120.91, 94.86, 56.12, 55.62, 27.71, 25.84, 17.80.

HRMS (ESI): m/z calcd for $C_{22}H_{23}O_3Br$ [M+H$^+$]: 415.0903; found: 415.0906.

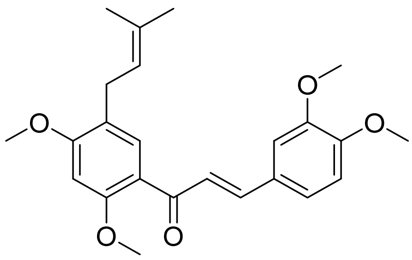

38f

(E)-1-(2,4-dimethoxy-5-(3-methylbut-2-en-1-yl)phenyl)-3-(3,4 dimethoxyphenyl)prop-2-en-1-one 38f:

^{1}H NMR (400 MHz, $CDCl_3$) δ 7.67 – 7.49 (m, 2H), 7.38 (d, *J* = 15.5 Hz, 1H), 7.17 (d, *J* = 8.4 Hz, 1H), 7.10 (s, 1H), 6.91 – 6.81 (m, 1H), 6.45 (s, 1H), 5.26 (d, *J* = 7.3 Hz, 1H), 3.91 (s, 12H), 3.25 (d, *J* = 7.3 Hz, 2H), 1.71 (s, 3H), 1.69 (s, 3H).

HRMS (ESI): m/z calcd for $C_{24}H_{28}O_5$ [M+H$^+$]: 397.2010; found: 397.1997.

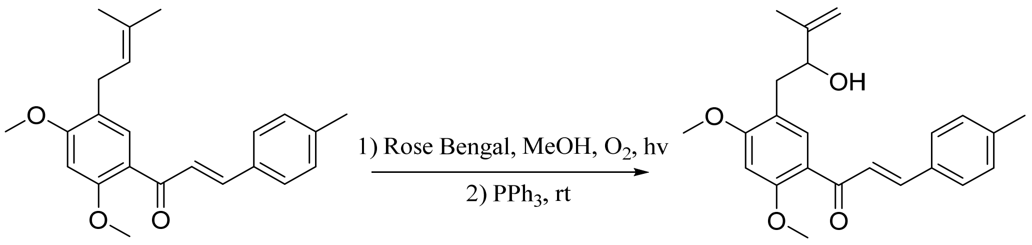

38a **39a**

(*E*)-1-(5-(2-hydroxy-3-methylbut-3-en-1-yl)-2,4-dimethoxyphenyl)-3-phenylprop-2-en-1-one:

Compound **39a** was synthesized by following a similar procedure as that of **28.**

^{1}H NMR (400 MHz, $CDCl_3$) δ 7.71 – 7.58 (m, 2H), 7.53 – 7.43 (m, 3H), 7.20 (d, *J* = 7.8 Hz, 2H), 6.48 (s, 1H), 4.94 (s, 1H), 4.83 (s, 1H), 4.29 (dd, *J* = 8.6, 4.2 Hz, 1H), 3.94 (s, 3H), 3.92 (s, 3H), 2.91 (dd, *J* = 13.9, 4.2 Hz, 1H), 2.77 (dd, *J* = 13.9, 8.6 Hz, 1H), 2.38 (s, 3H), 1.81 (s, 3H).

^{13}C NMR (100 MHz, $CDCl_3$) δ 190.57, 161.86, 159.52, 147.18, 142.20, 140.37, 133.72, 132.78, 129.60, 128.34, 126.33, 121.48, 119.44, 110.78, 95.11, 75.60, 56.08, 55.70, 36.17, 21.51, 18.09.

HRMS (ESI): m/z calcd for $C_{23}H_{26}O_4$ [M+H$^+$]: 367.1904; found: 367.1895.

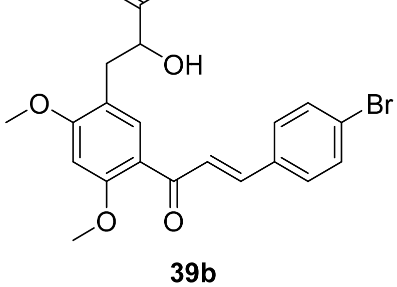

39b

(E)-3-(4-bromophenyl)-1-(5-(2-hydroxy-3-methylbut-3-en-1-yl)-2,4 dimethoxyphenyl)prop-2-en-1-one 39b:

^{1}H NMR (400 MHz, $CDCl_3$) δ 7.64 (d, J = 2.3 Hz, 2H), 7.55 – 7.42 (m, 5H), 6.48 (d, J = 2.2 Hz, 1H), 4.94 (s, 1H), 4.83 (s, 1H), 4.29 (dd, J = 8.9, 3.8 Hz, 1H), 3.95 (s, 3H), 3.93(s, 3H), 2.90 (dd, J = 3.8, 2.3 Hz, 1H), 2.79 (dd, J = 8.9, 2.3 Hz, 1H), 1.81 (s, 3H).

^{13}C NMR (100 MHz, $CDCl_3$) δ 189.97, 162.19, 159.72, 147.17, 140.40, 134.52, 133.85, 132.08, 129.69, 127.81, 124.06, 121.06, 119.61, 110.83, 94.96, 75.57, 56.07, 55.74, 36.12, 18.10.

HRMS (ESI): m/z calcd for $C_{22}H_{23}O_4Br$ [M+H$^+$]: 431.0852; found: 431.0857.

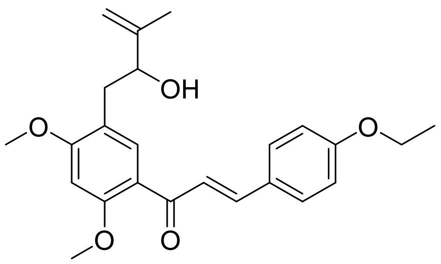

39c

(E)-3-(4-ethoxyphenyl)-1-(5-(2-hydroxy-3-methylbut-3-en-1-yl)-2,4 dimethoxyphenyl)prop-2-en-1-one 39c:

^{1}H NMR (400 MHz, $CDCl_3$) δ 7.69 – 7.50 (m, 4H), 7.44 – 7.36 (m, 1H), 6.94 – 6.86 (m, 2H), 6.48 (d, J = 2.2 Hz, 1H), 4.95 (s, 1H), 4.83 (d, J = 2.6 Hz, 1H), 4.29 (dd, J = 8.5, 6.3 Hz, 1H), 4.07 (q, J = 5.8 Hz, 2H), 3.94 (s, 3H), 3.93 (s, 3H), 2.91 (dd, J = 14.0, 6.3 Hz, 1H), 2.78 (dd, J = 14.0, 8.5 Hz, 1H), 1.81 (s, 3H), 1.44 (t, J = 5.8 Hz, 3H).

^{13}C NMR (100 MHz, $CDCl_3$) δ 190.60, 161.72, 160.67, 159.42, 147.18, 142.21, 133.69, 130.04, 128.02, 124.92, 121.61, 119.34, 114.79, 110.80, 95.09, 75.65, 63.62, 56.10, 55.71, 36.19, 18.12, 14.79.

HRMS (ESI): m/z calcd for $C_{24}H_{28}O_5$ [M+H$^+$]: 397.2010; found: 397.2009.

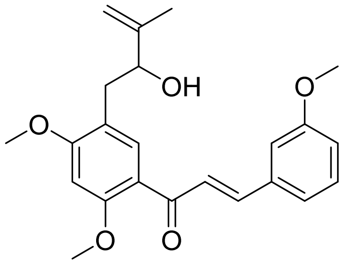

39d

(E)-1-(5-(2-hydroxy-3-methylbut-3-en-1-yl)-2,4-dimethoxyphenyl)-3-(3-

methoxyphenyl)prop-2-en-1-one 39d

^{1}H NMR (400 MHz, $CDCl_3$) δ 7.64 (d, *J* = 15.0 Hz, 2H), 7.50 (d, *J* = 15.8 Hz, 1H), 7.31 (t, *J* = 7.8 Hz, 1H), 7.20 (d, *J* = 7.7 Hz, 1H), 7.12 (d, *J* = 2.5 Hz, 1H), 6.93 (dd, *J* = 8.1, 2.7 Hz, 1H), 6.48 (s, 1H), 4.94 (s, 1H), 4.83 (s, 1H), 4.29 (dd, *J* = 8.5, 4.2 Hz, 1H), 3.94 (s, 3H), 3.93 (s, 3H), 3.84 (s, 3H), 2.91 (dd, *J* = 13.9, 4.2 Hz, 1H), 2.77 (dd, *J* = 13.9, 8.5 Hz, 1H), 1.81 (s, 3H).

^{13}C NMR (100 MHz, $CDCl_3$) δ 190.38, 162.01, 159.87, 159.63, 147.18, 141.87, 136.97, 133.78, 129.82, 127.60, 121.29, 120.95, 119.50, 115.58, 113.48, 110.81, 95.04, 75.59, 56.07, 55.71, 55.32, 36.14, 18.09.

HRMS (ESI): m/z calcd for $C_{23}H_{26}O_5$ [M+H$^+$]: 383.1853; found: 383.1841.

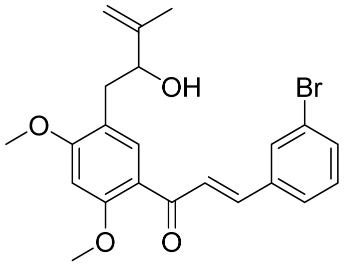

39e

(E)-3-(3-bromophenyl)-1-(5-(2-hydroxy-3-methylbut-3-en-1-yl)-2,4-dimethoxyphenyl)prop-2-en-1-one 39e

^{1}H NMR (400 MHz, $CDCl_3$) δ 7.70 – 7.29 (m, 7H), 6.41 (s, 1H), δ 4.87 (s, 1H), 4.76 (s, 1H), 4.22 (dd, *J* = 8.8, 4.2 Hz, 1H), 3.95 (s, 3H), 3.93 (s, 3H), 2.84 (dd, *J* = 13.9, 4.2 Hz, 1H), 2.70 (dd, *J* = 13.9, 8.8 Hz, 1H), 1.74 (s, 3H).

^{13}C NMR (100 MHz, $CDCl_3$) δ 189.97, 162.19, 159.72, 147.17, 140.41, 134.52, 133.85, 132.08, 129.68, 127.82, 124.07, 121.08, 119.61, 110.83, 94.97, 75.57, 56.08, 55.73, 36.12, 18.10.

HRMS (ESI): m/z calcd for $C_{22}H_{23}O_4Br$ [M+H$^+$]: 431.0852; found: 431.0865.

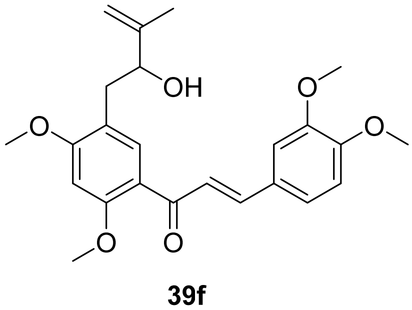

39f

(E)-3-(3,4-dimethoxyphenyl)-1-(5-(2-hydroxy-3-methylbut-3-en-1-yl)-2,4-dimethoxyphenyl)prop-2-en-1-one 39f

^1H NMR (400 MHz, $CDCl_3$) δ 7.67 – 7.56 (m, 2H), 7.35 (dd, *J* = 15.7, 10.5 Hz, 1H), 7.19 (dd, *J* = 8.3, 2.0 Hz, 1H), 7.12 (d, *J* = 2.0 Hz, 1H), 6.88 (d, *J* = 8.3 Hz, 1H), 6.49 (d, *J* = 5.9 Hz, 1H), 4.94 (s, 1H), 4.82 (s, 1H), 4.29 (dd, *J* = 8.5, 4.2 Hz, 1H), 3.92 (t, *J* = 2.6 Hz, 12H), 2.91 (dd, *J* = 13.9, 4.3 Hz, 1H), 2.77 (dd, *J* = 13.9, 8.5Hz, 1H), 1.81 (s, 3H).

^{13}C NMR (100 MHz, $CDCl_3$) δ 190.61, 161.72, 159.36, 150.98, 149.16, 147.19, 142.42, 133.63, 131.41, 128.50, 125.39, 122.60, 119.43, 111.14, 110.77, 110.31, 95.18, 75.61, 56.10, 56.00, 55.92, 55.70, 36.17, 18.09.

HRMS (ESI): m/z calcd for $C_{24}H_{28}O_6$ $[M+H^+]$: 413.1959; found: 413.1975.

5. Biological study

5.1 Cell Culture and anti-inflammatory experiments procedure in vitro.

The derived cells were cultured using DMEM/F12 medium supported with 10% fetal bovine serum and penicillin-streptomycin solution and seeded in T75 cell culture flasks and placed in 5% CO_2 atmosphere at 37°C. RAW264.7 macrophage cells (5 × 10^4 cells/well) were plated in 24-well plates. After incubation for 24 h, the cells were treated with compounds (20 μM) for 1 h, then these cells were stimulated with LPS (5 μg/ml) for 12 h. The supernatant from the cell pellet were collected separately after the treatment. The medium collected was centrifuged at 1200 rpm for 5 min. The cytokine levels (TNF- α and IL-6) were measured by means of commercially available Enzyme-Linked Immuno-Sorbent Assay (ELISA) according to the manufacturer's instructions[4].

5,2 CCK-8 assay.

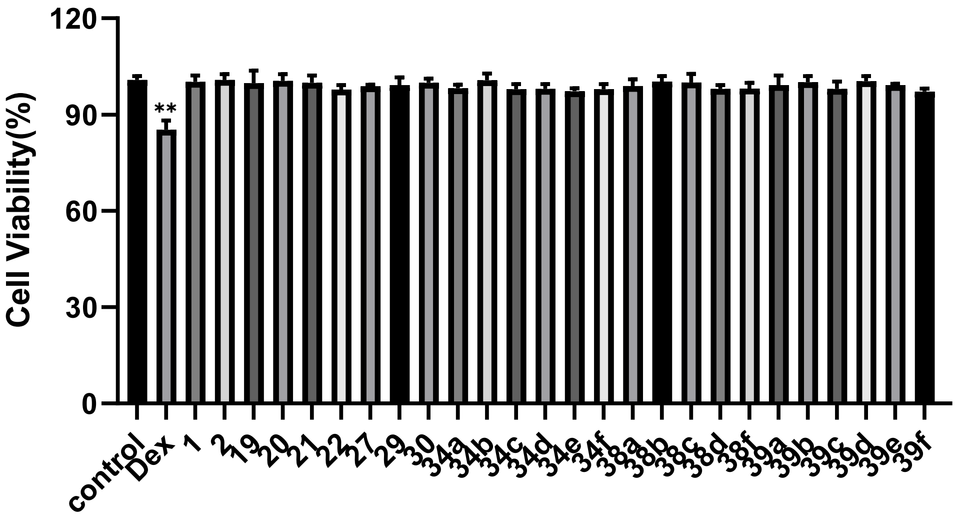

Figure S1. Cell viability of RAW264.7 macrophages induced by synthetic compounds after 24 h

by CCK-8 assay.

CCK-8 assay. Procedure: RAW264.7 macrophages were first counted and approximately 4000 cells per well were seeded in a 96-well cell culture plate. Then, after incubation at 37°Cina humidified atmosphere with 5% CO_2 for 24 h, the culture medium was replaced by a series of concentrations of drug sdiluted with the corresponding culture fluid. Five replicates were made for each measurement, and the time of co-incubation was determined by the efficiency of each drug. In this study, these synthetic compounds were co-incubated with the cells for 24 h at 37°C under the same conditions as described above, Finally, 10μL of the CCK-8 reagent was added into each well, and OD at 450 nm was measured using a multifunction microplate reader after incubation for 2 h at 37°C. The percentage each concentration accounted for of the control was presented as cell viability

6. NMR Spectras

^{1}H NMR of compound 9

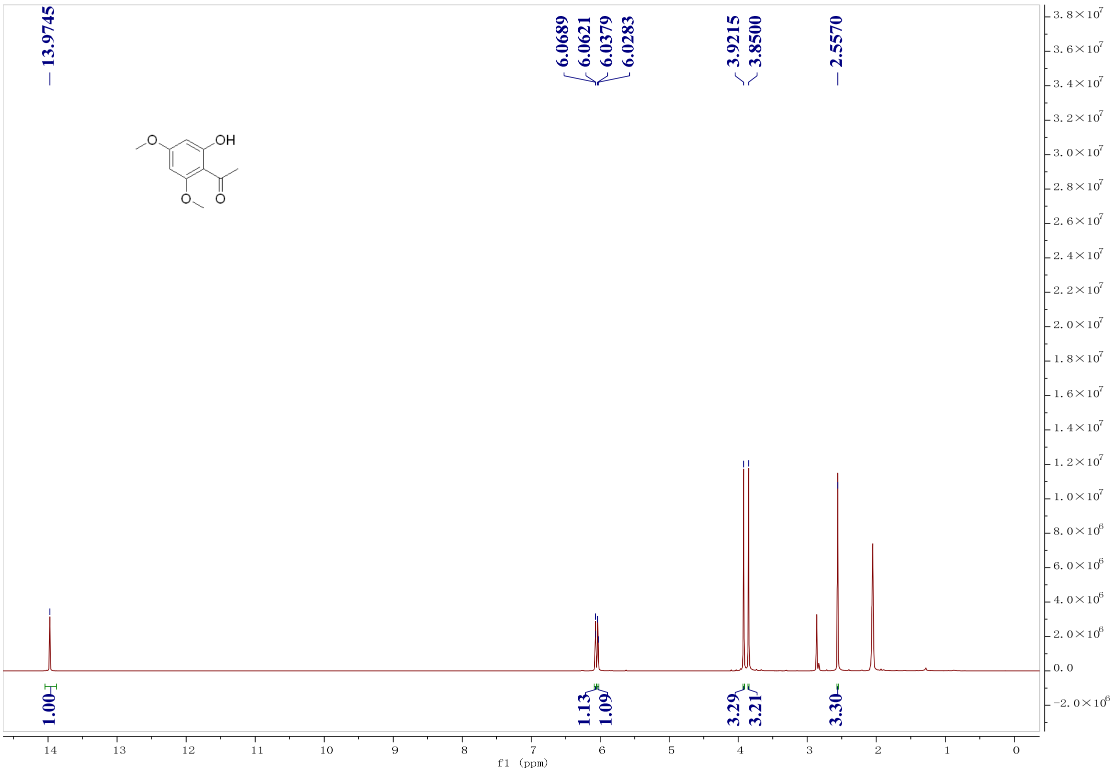

^{13}C NMR of compound 9

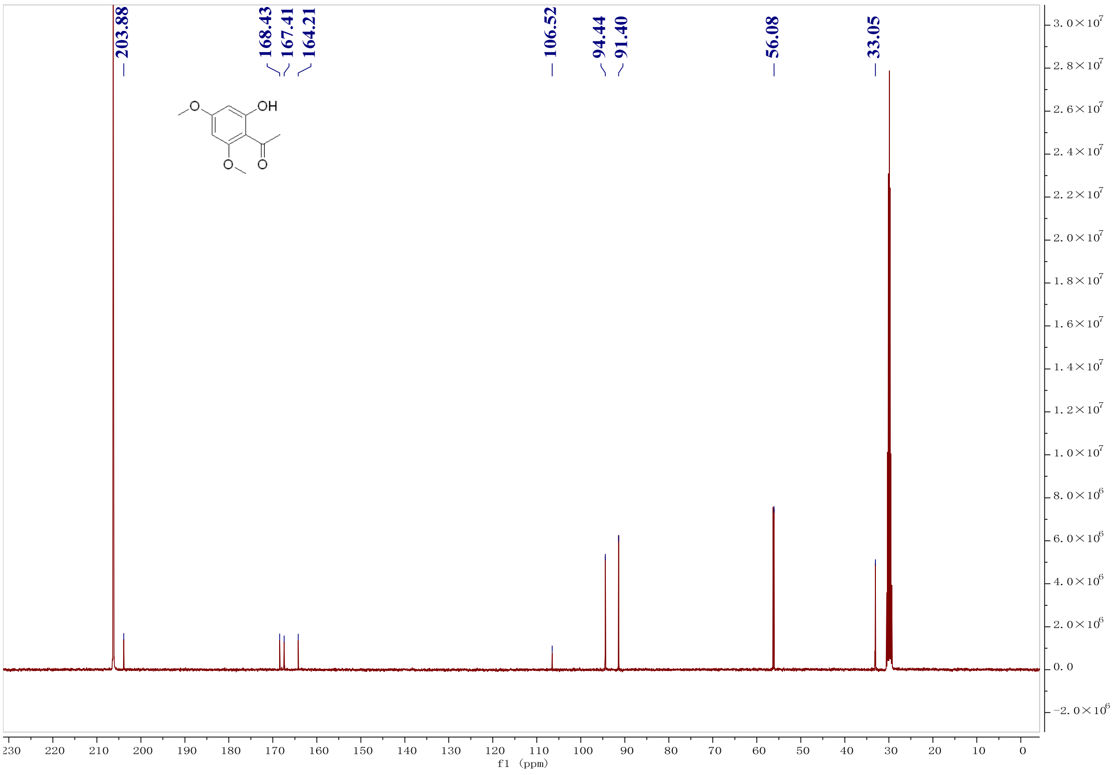

^{1}H NMR of compound 10

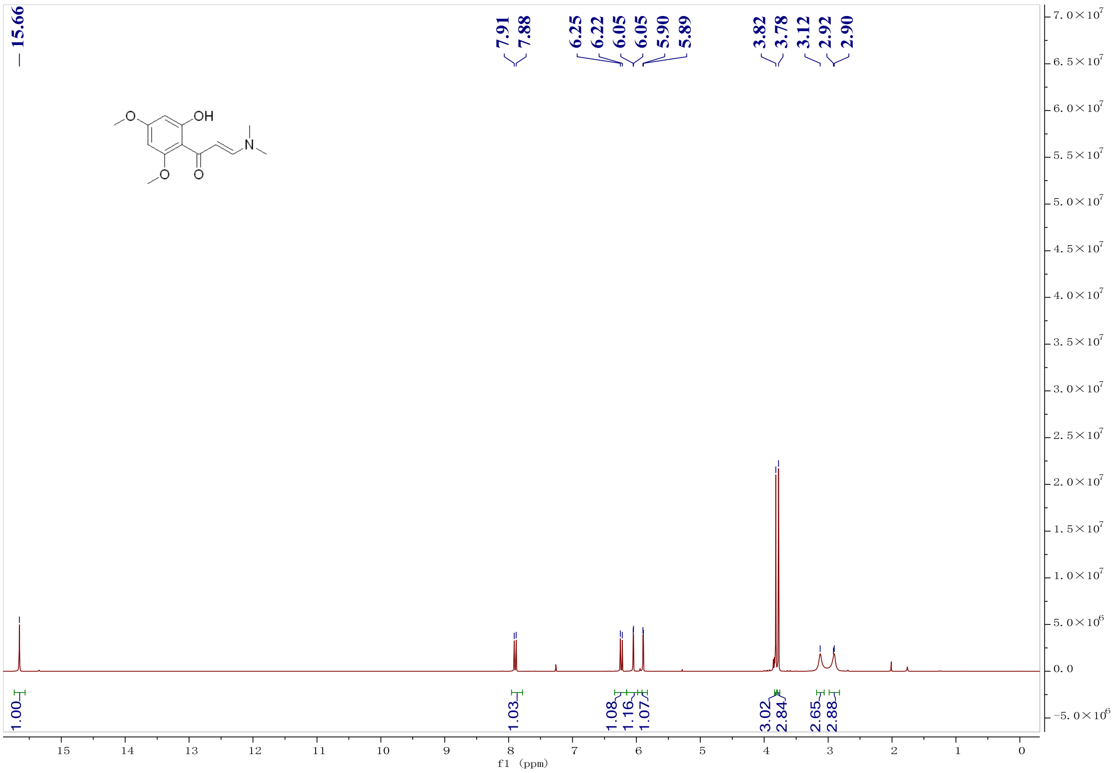

^{13}C NMR of compound 10

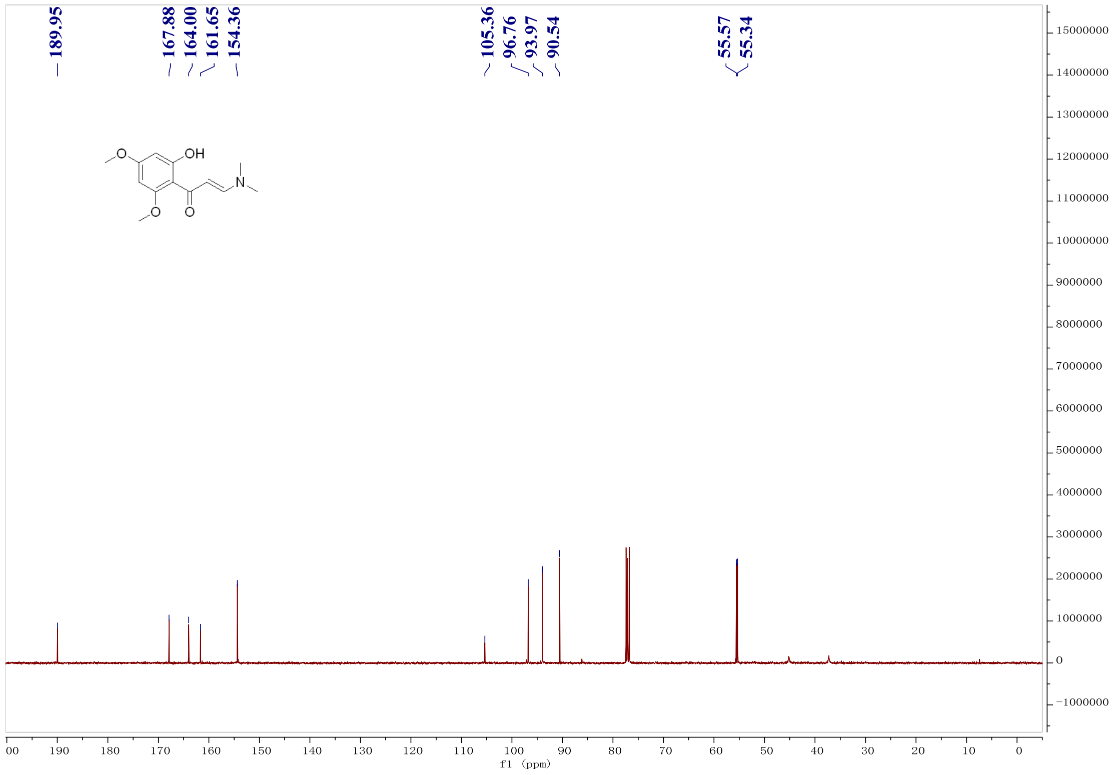

^{1}H NMR of compound 11

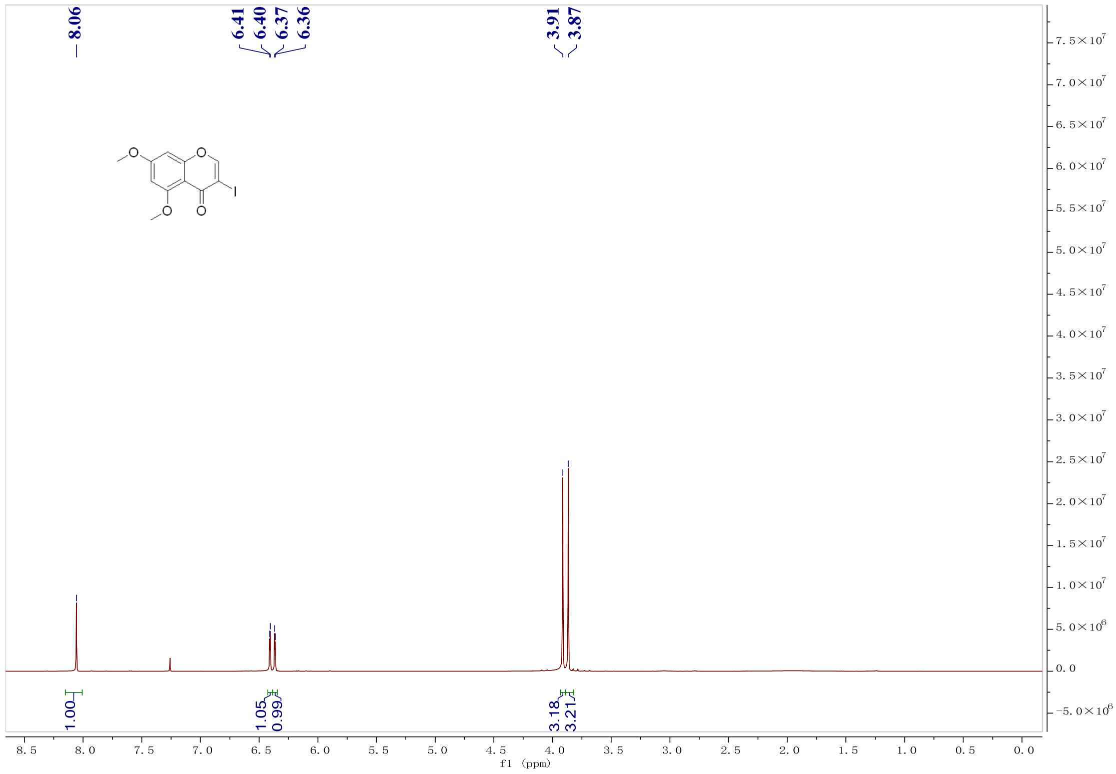

^{13}C NMR of compound 11

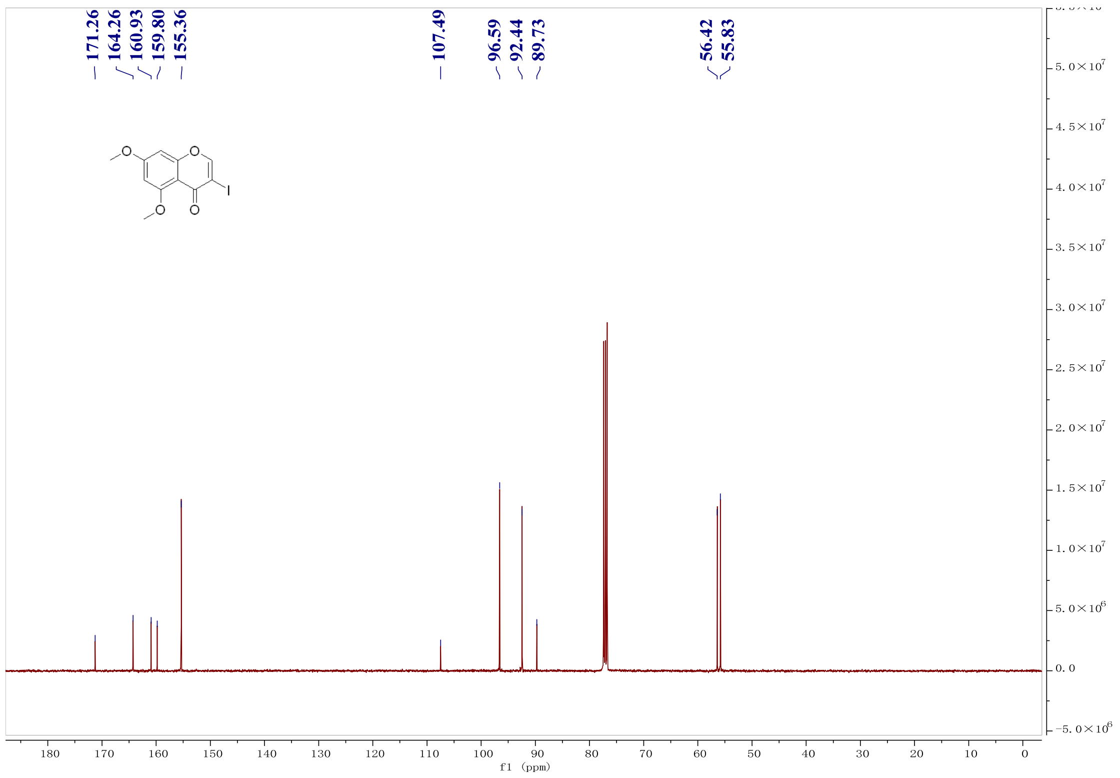

^{1}H NMR of compound 12

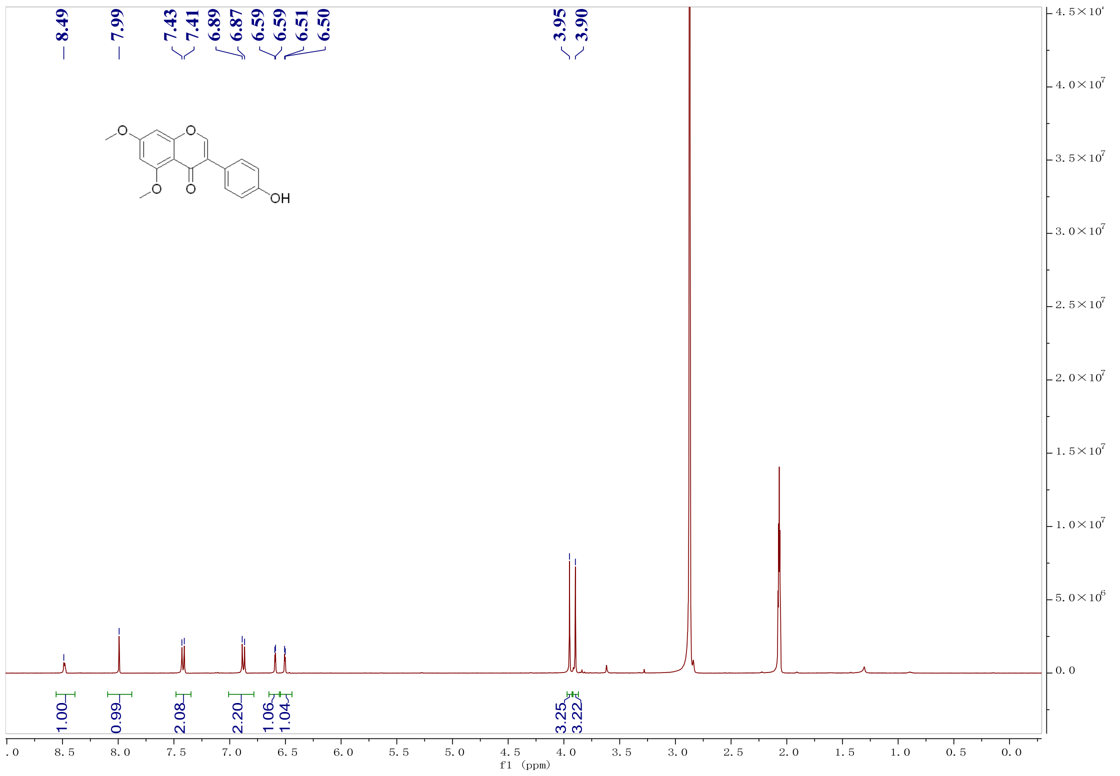

^{13}C NMR of compound 12

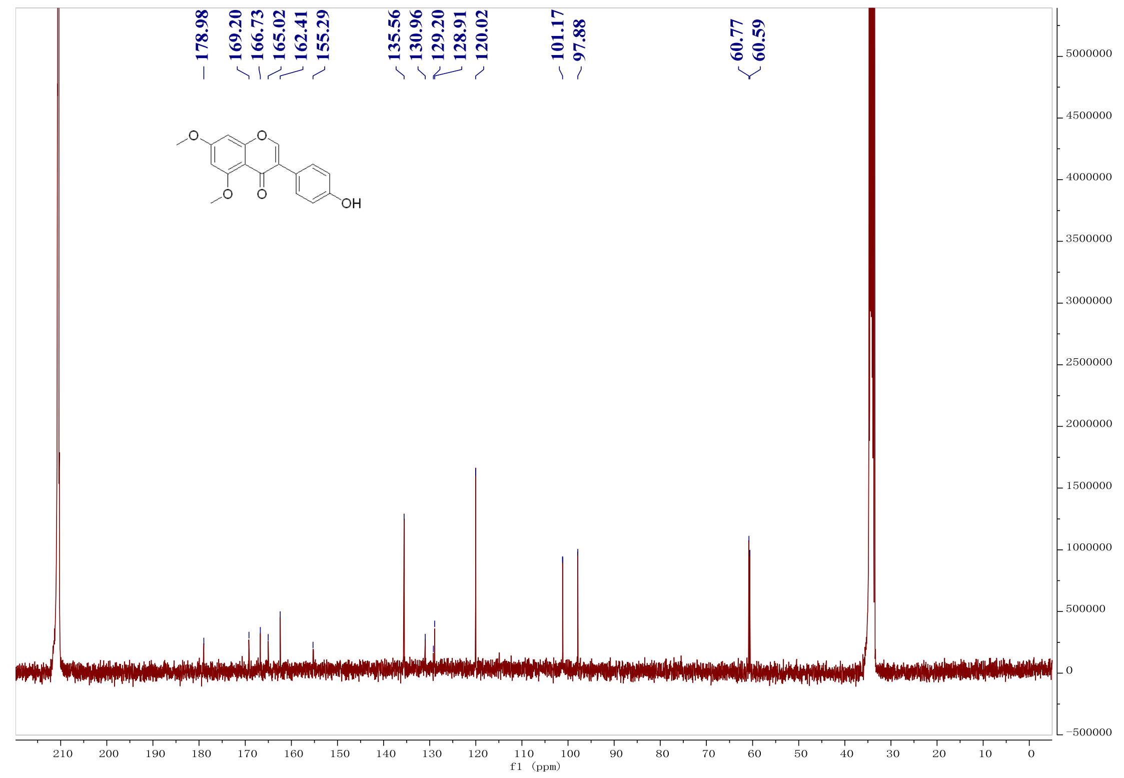

^{1}H NMR of compound 3

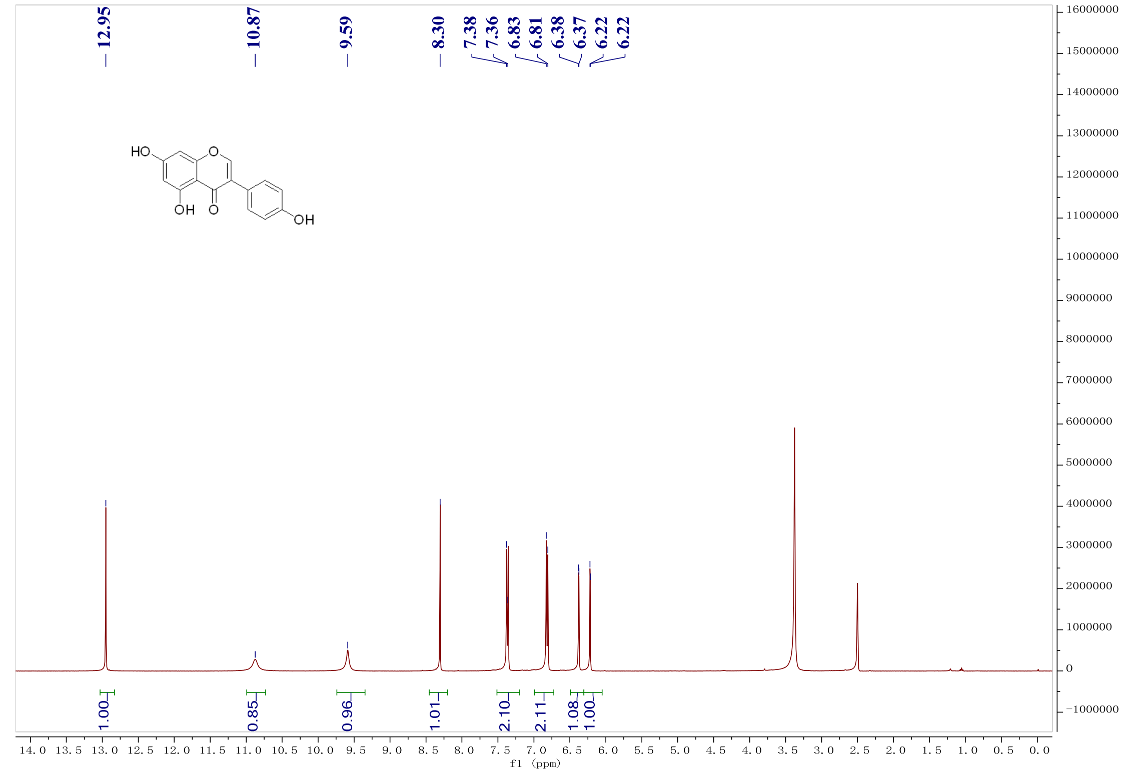

^{13}C NMR of compound 3

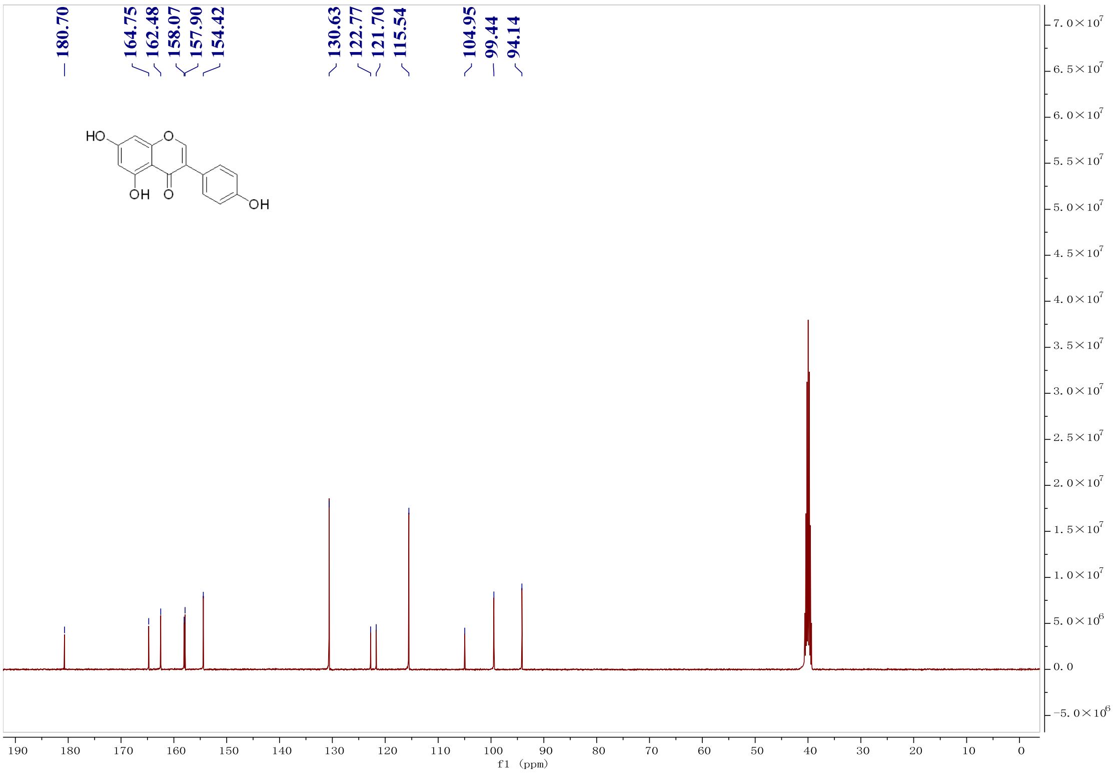

^{1}H NMR of compound 13

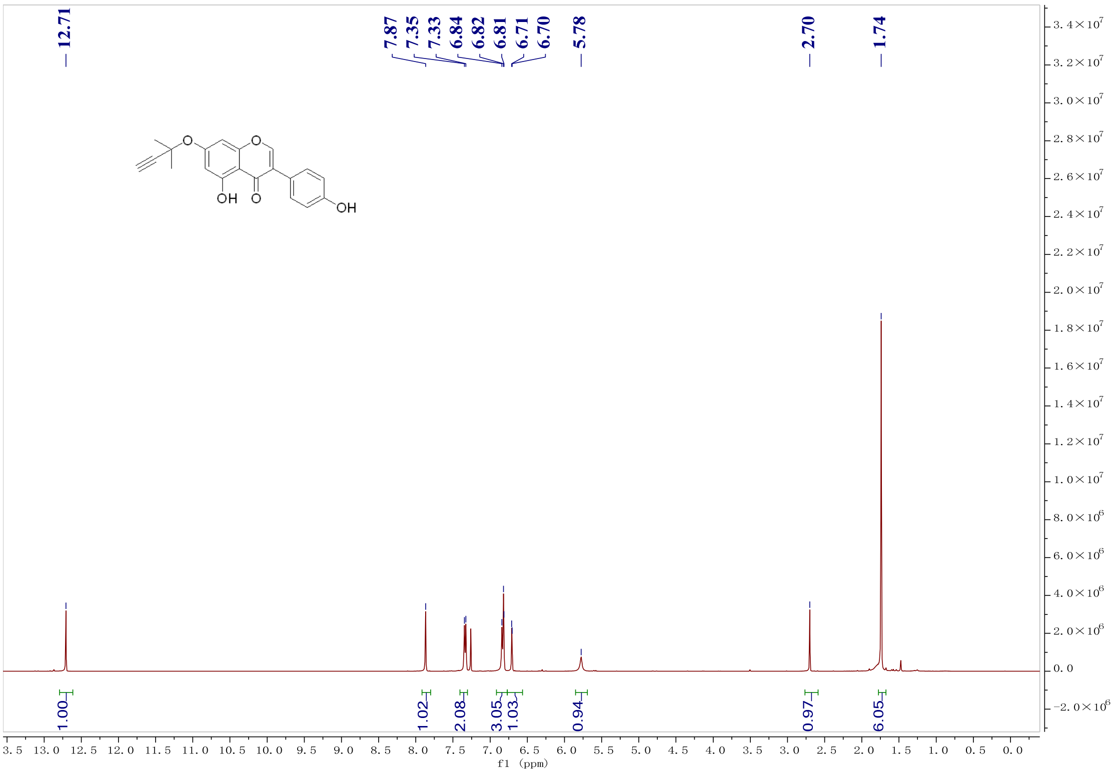

^{13}C NMR of compound 13

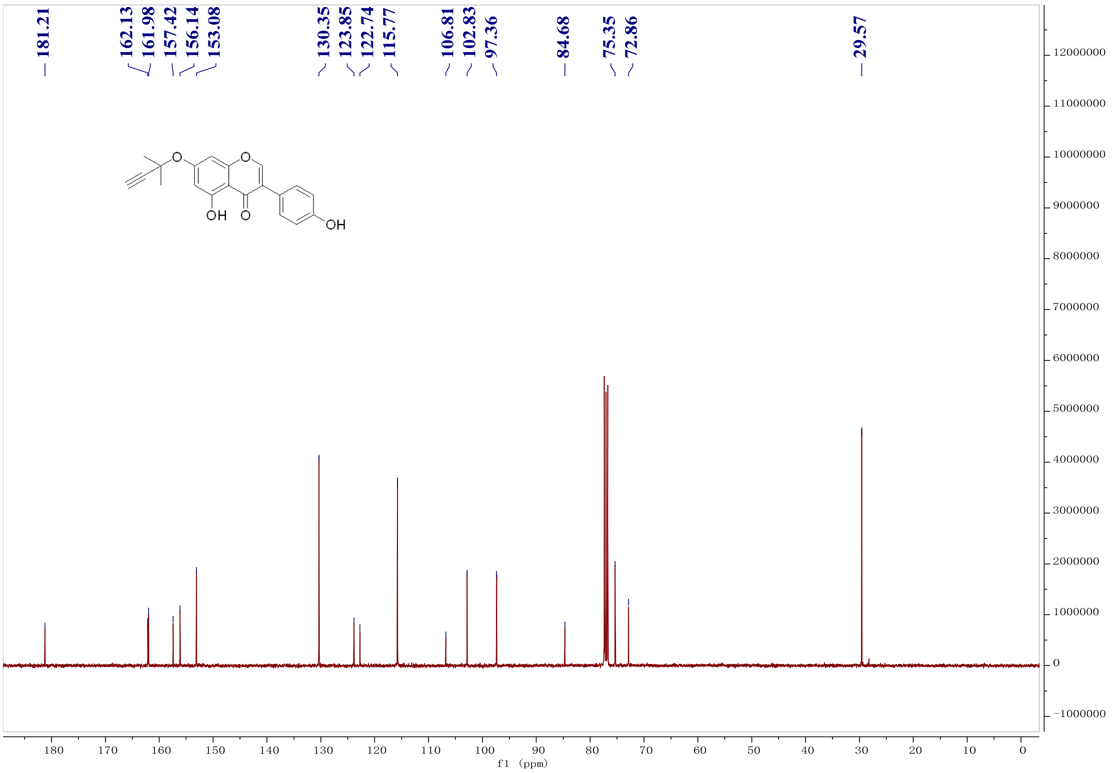

^{1}H NMR of compound 14

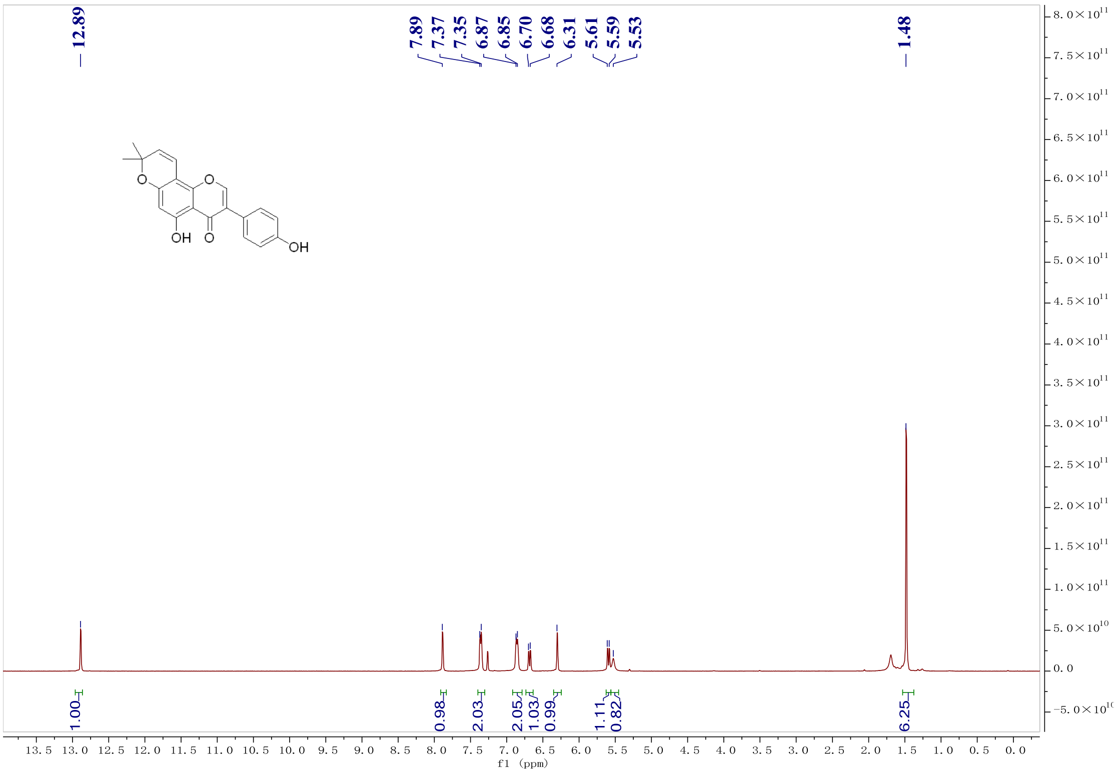

^{13}C NMR of compound 14

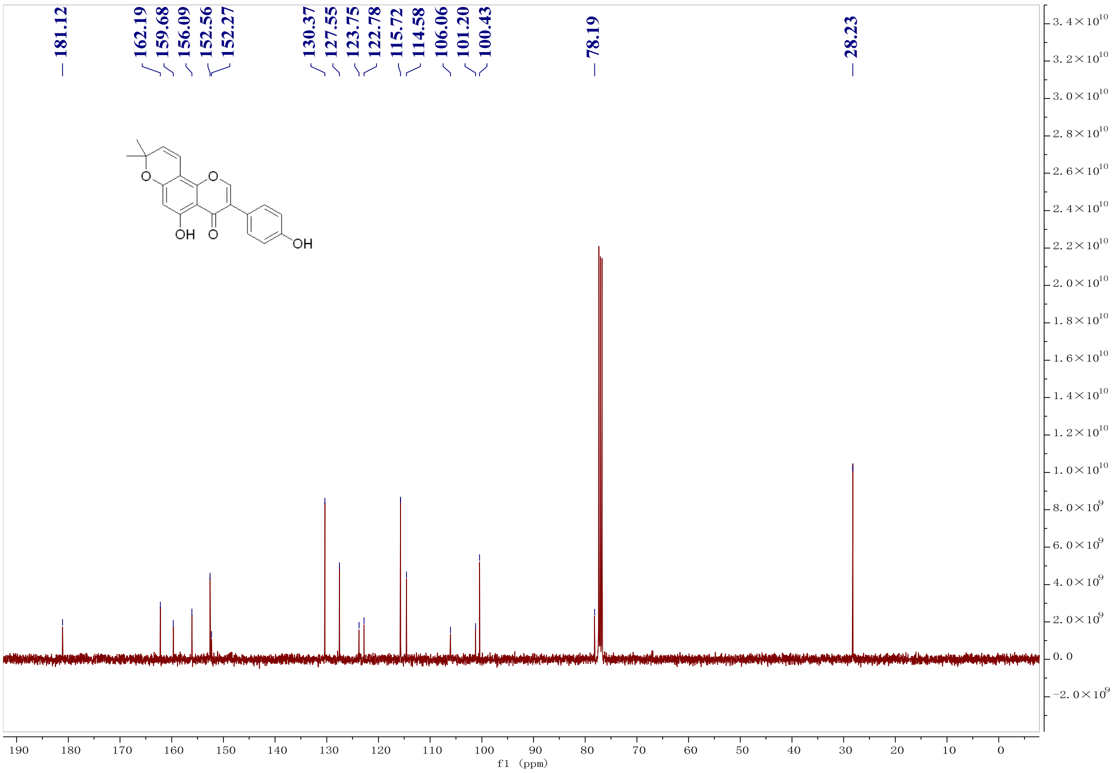

^{1}H NMR of compound 15

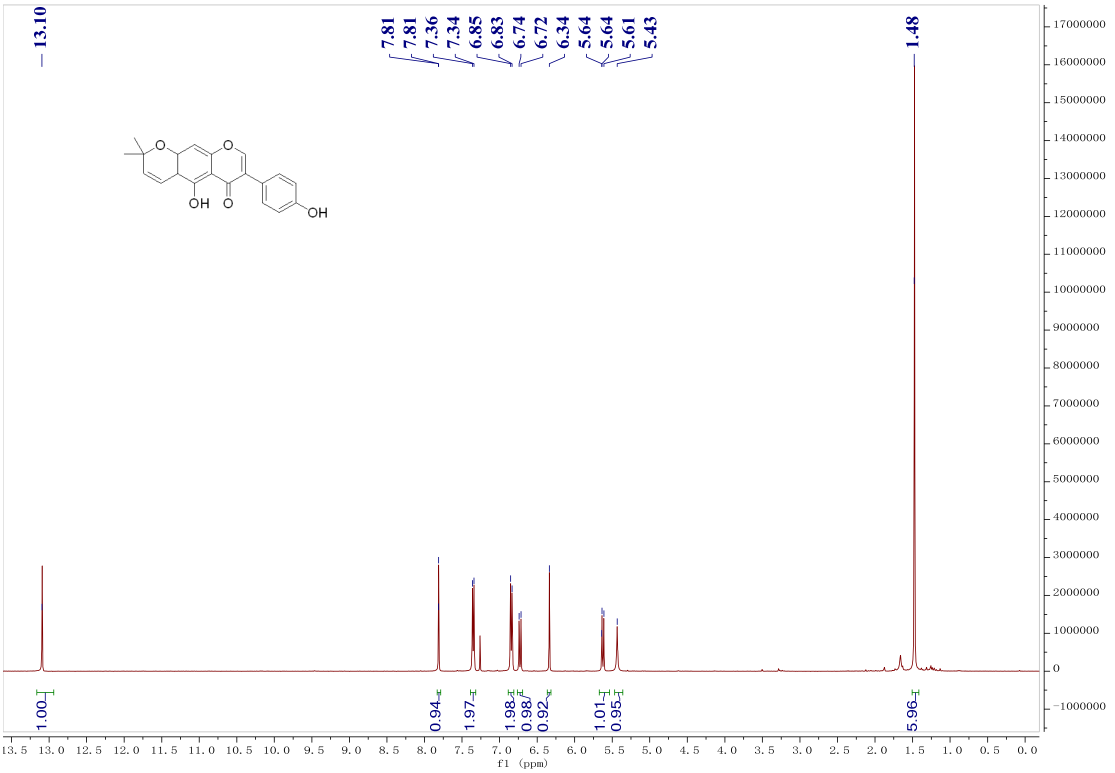

^{13}C NMR of compound 15

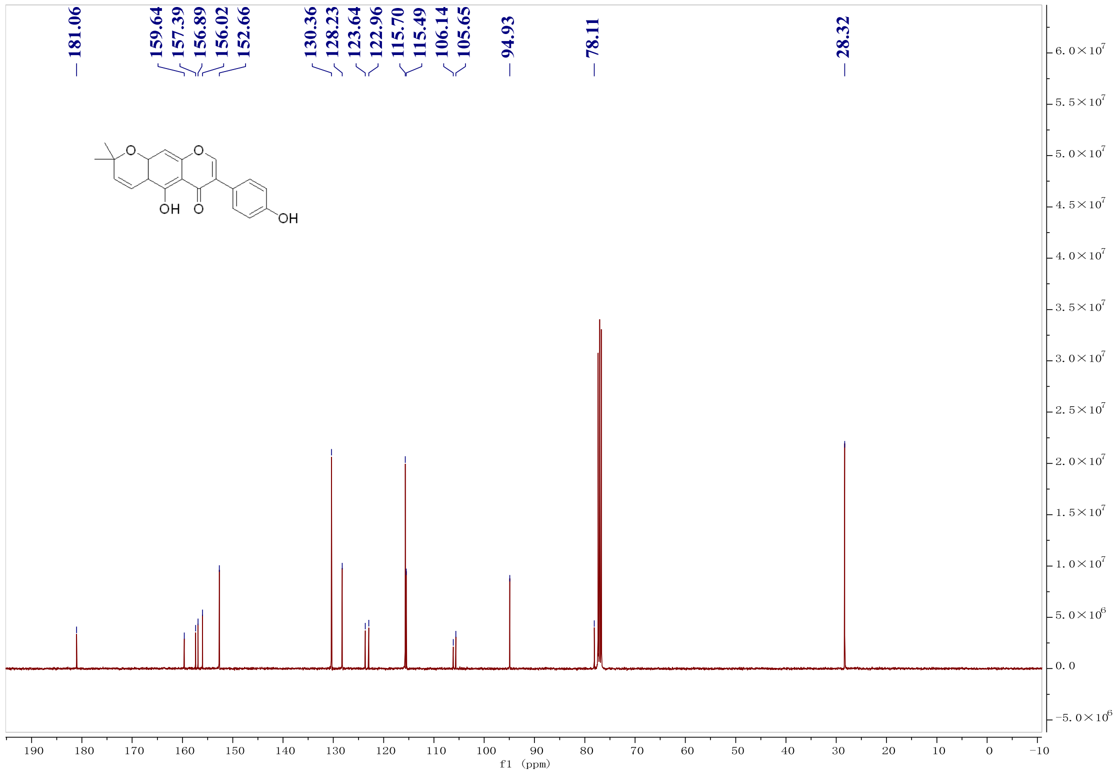

^{1}H NMR of compound 23

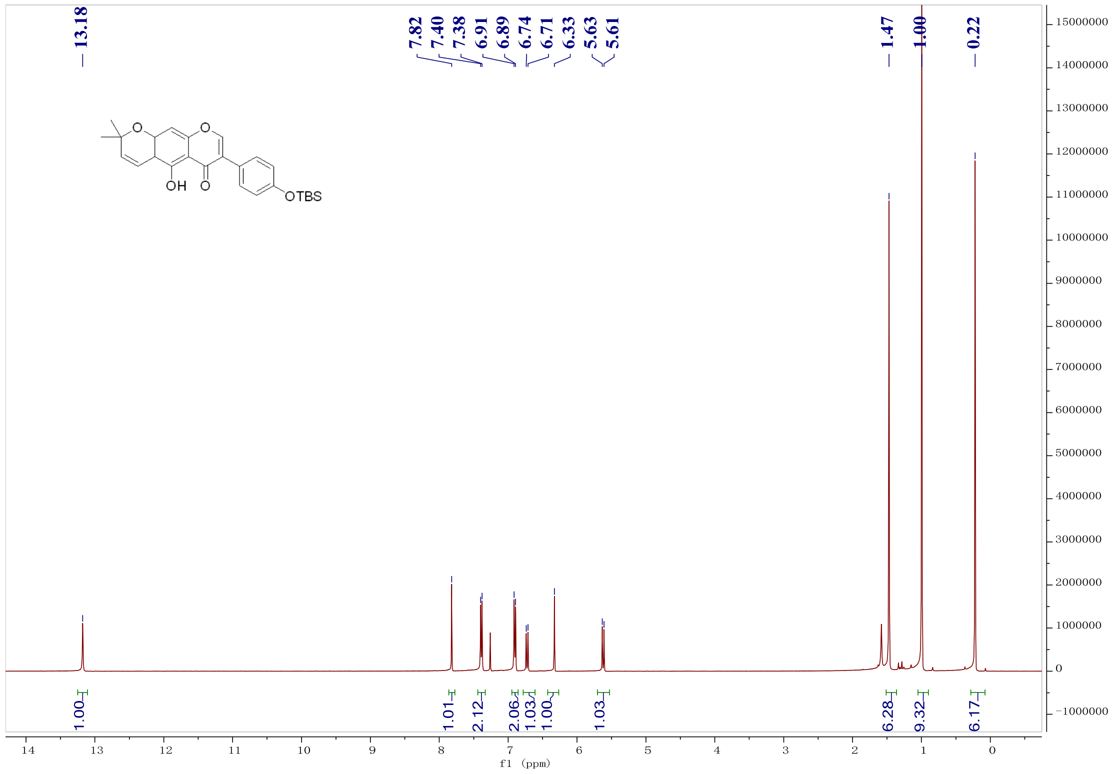

^{13}C NMR of compound 23

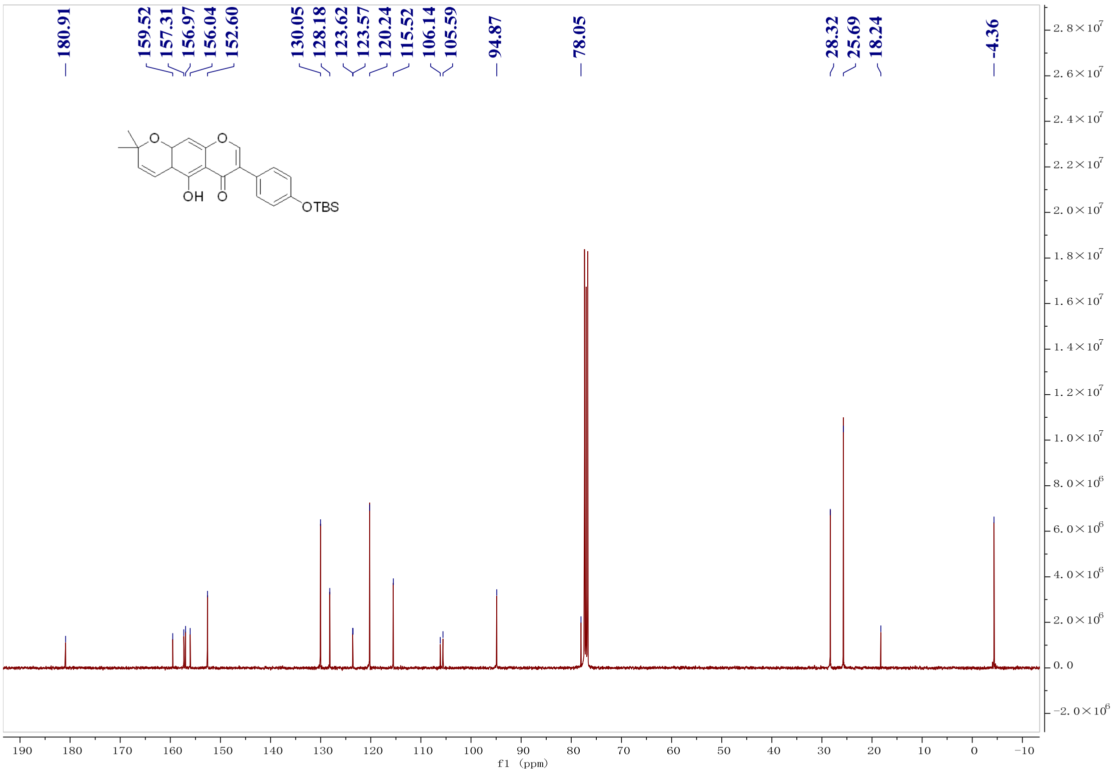

^{1}H NMR of compound 17

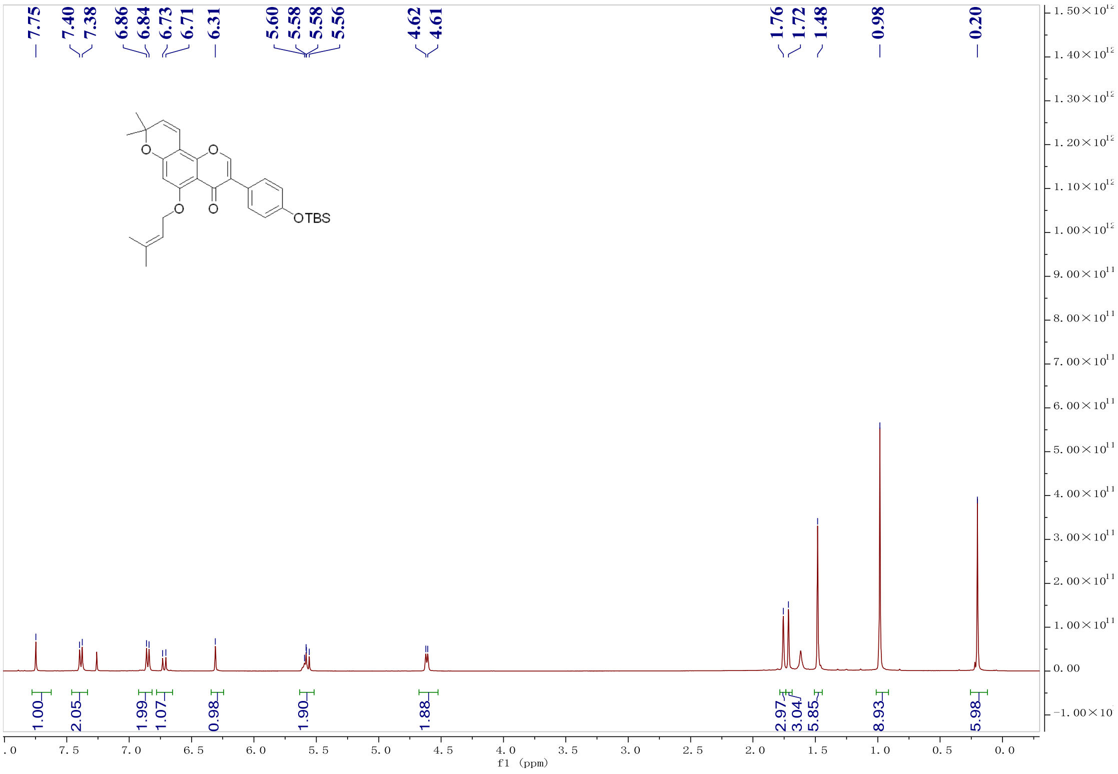

^{13}C NMR of compound 17

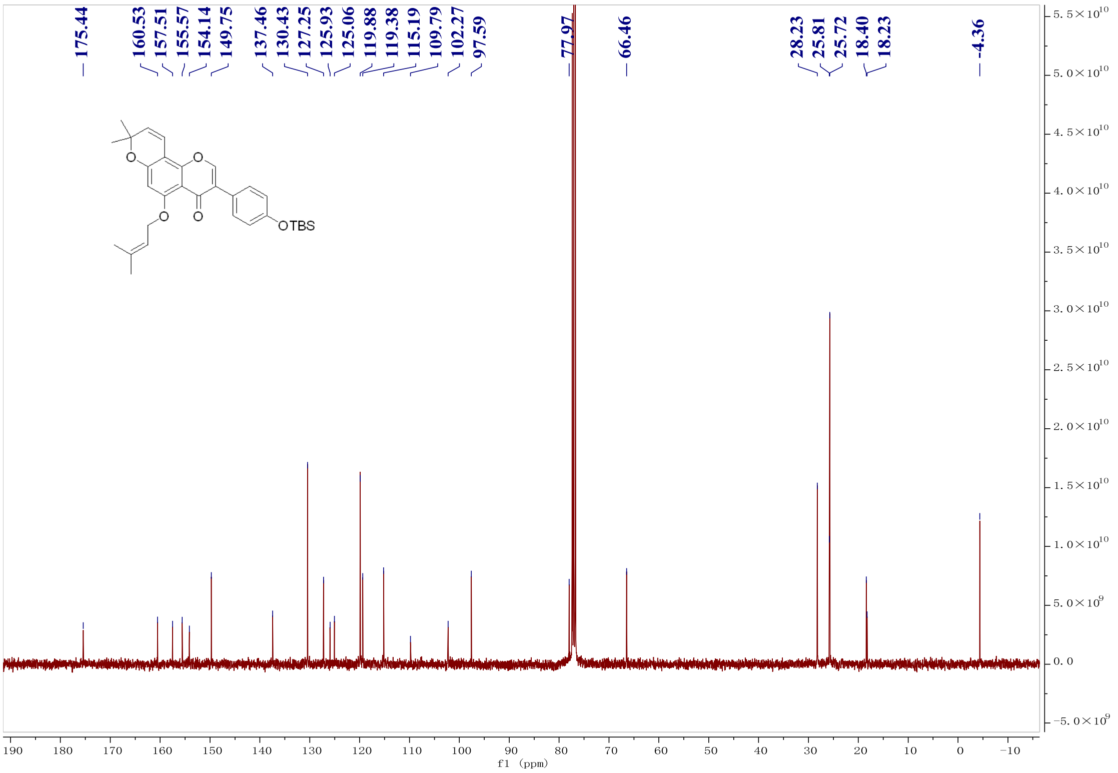

^{1}H NMR of compound 24

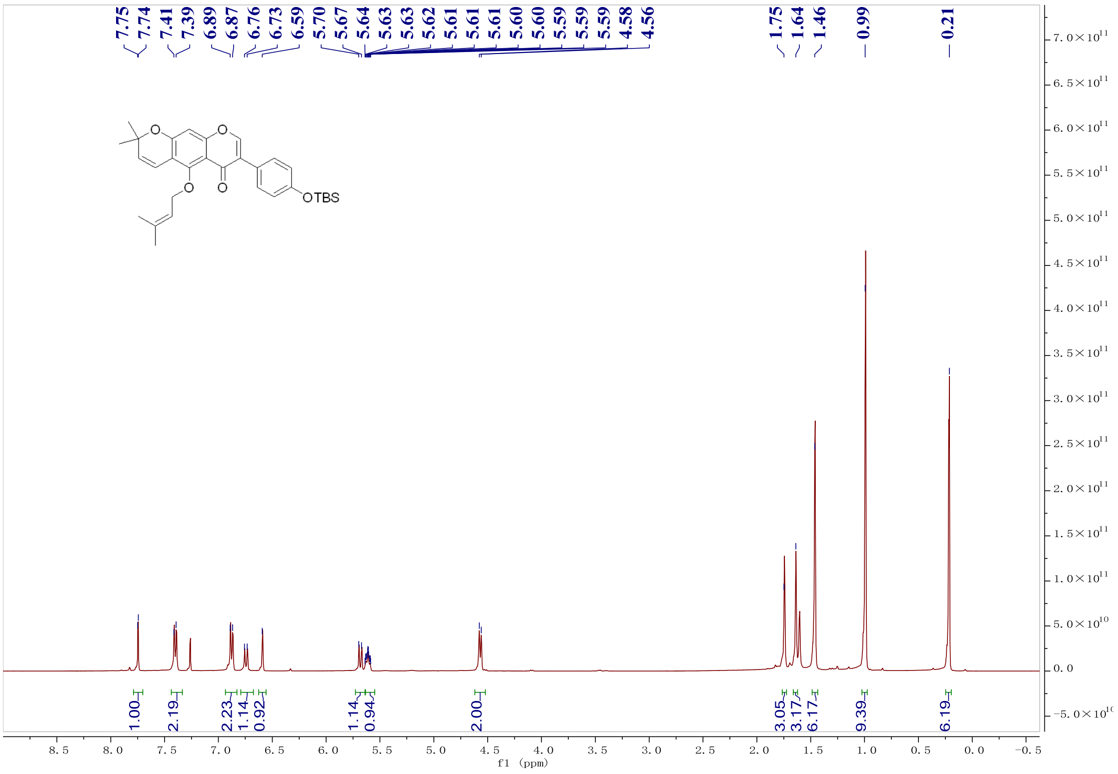

^{13}C NMR of compound 24

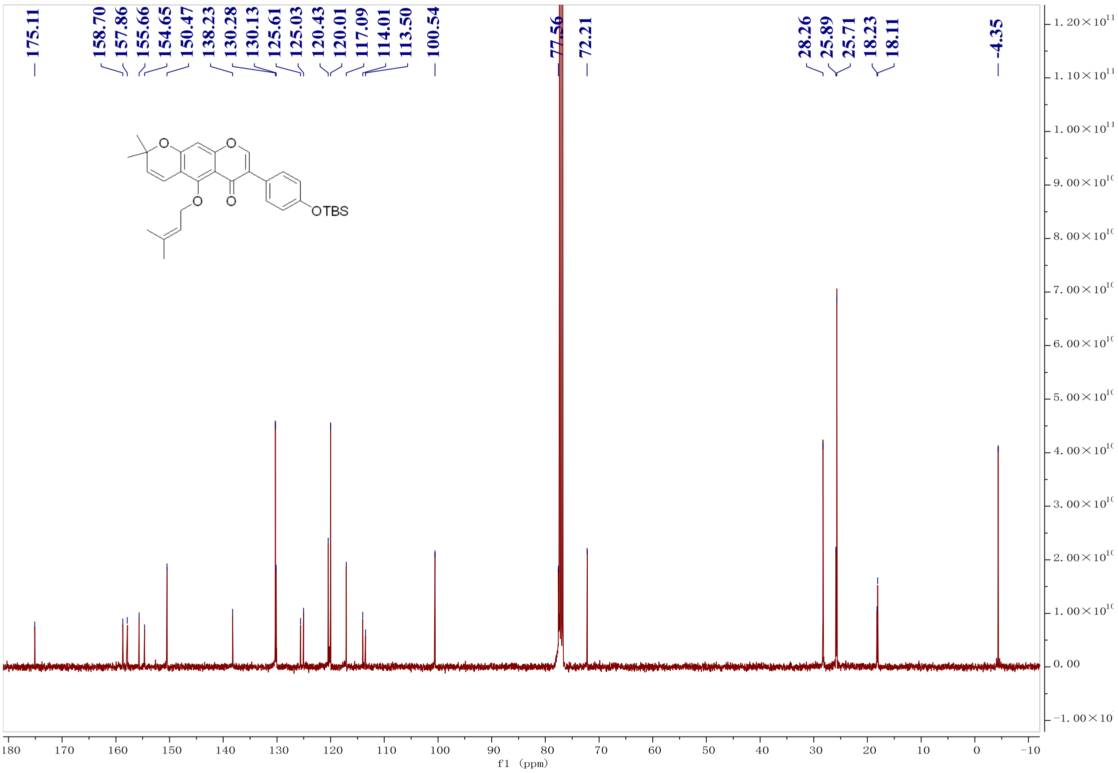

^{1}H NMR of compound 25

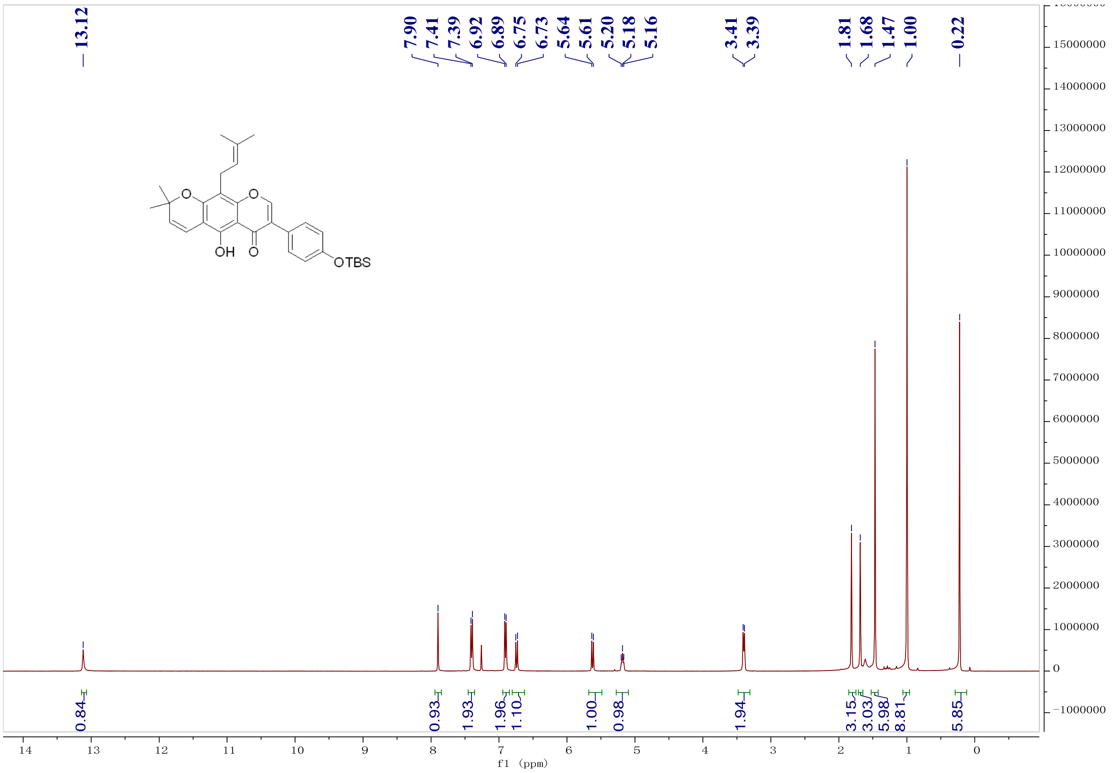

^{13}C NMR of compound 25

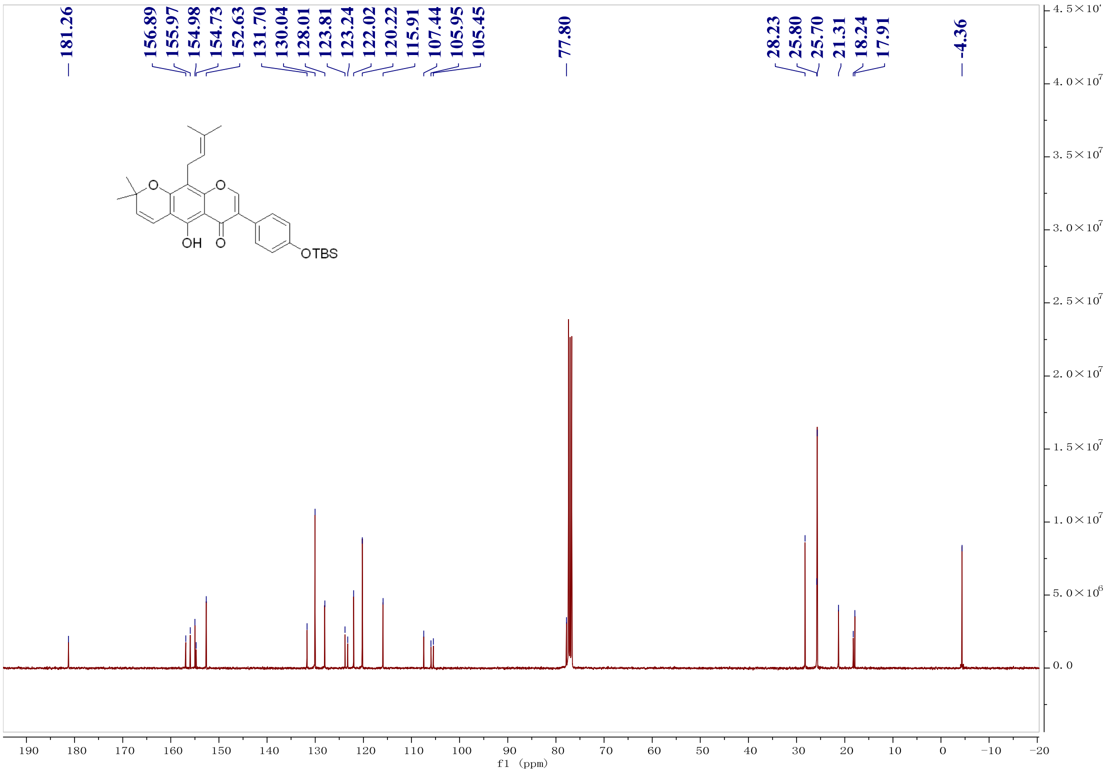

^{1}H NMR of compound 26

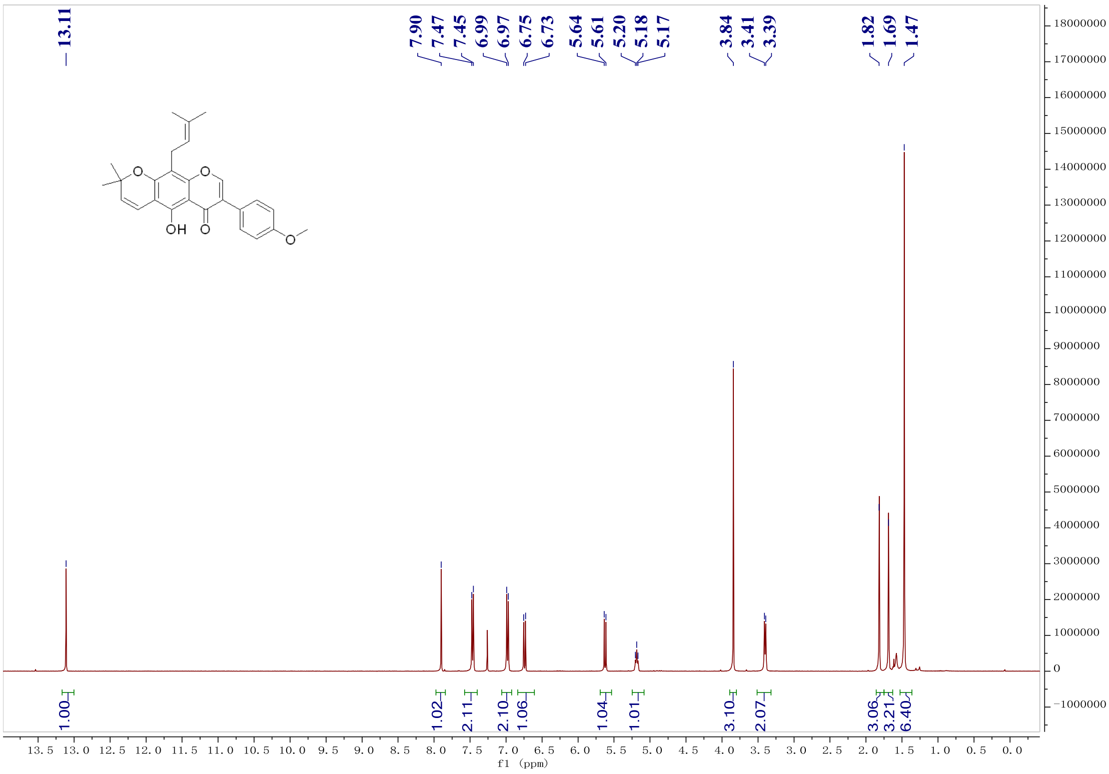

^{13}C NMR of compound 26

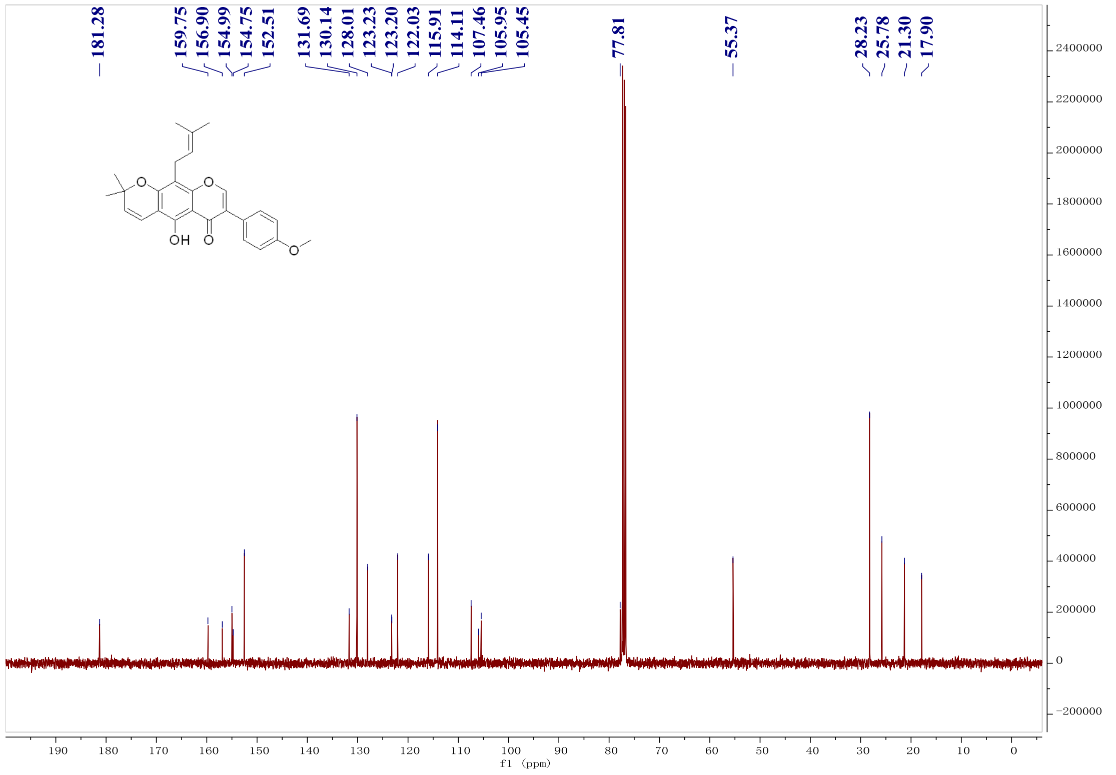

^{1}H NMR of compound 27

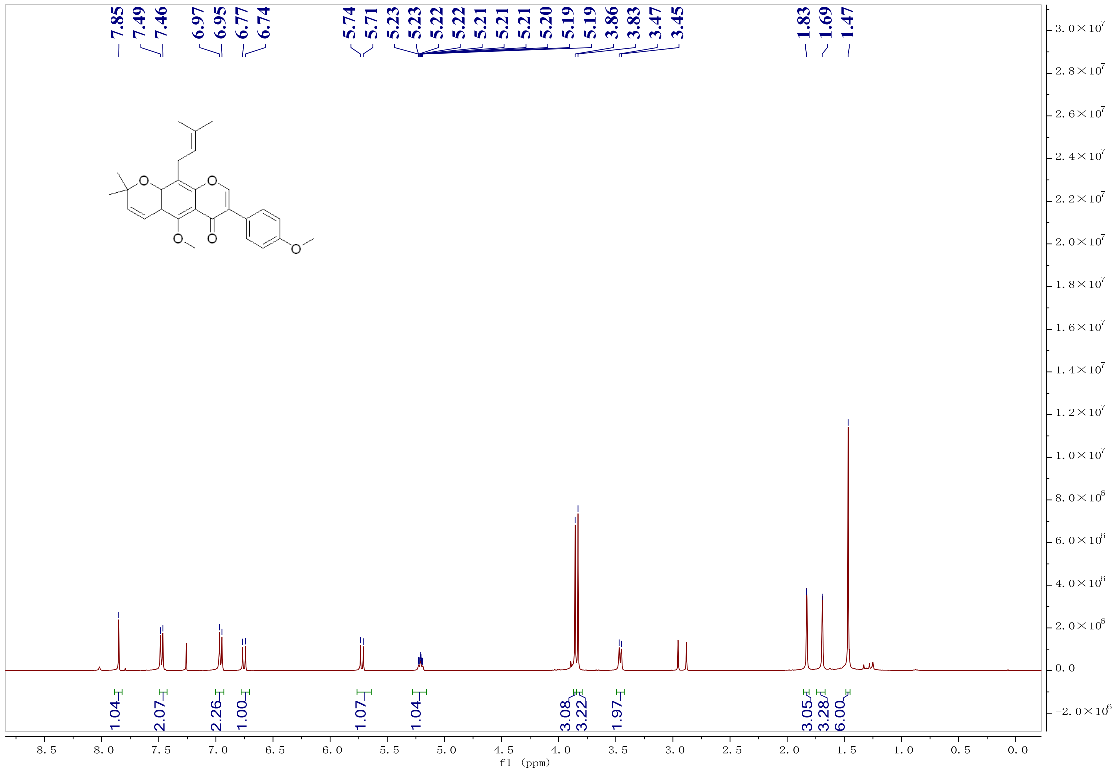

^{13}C NMR of compound 27

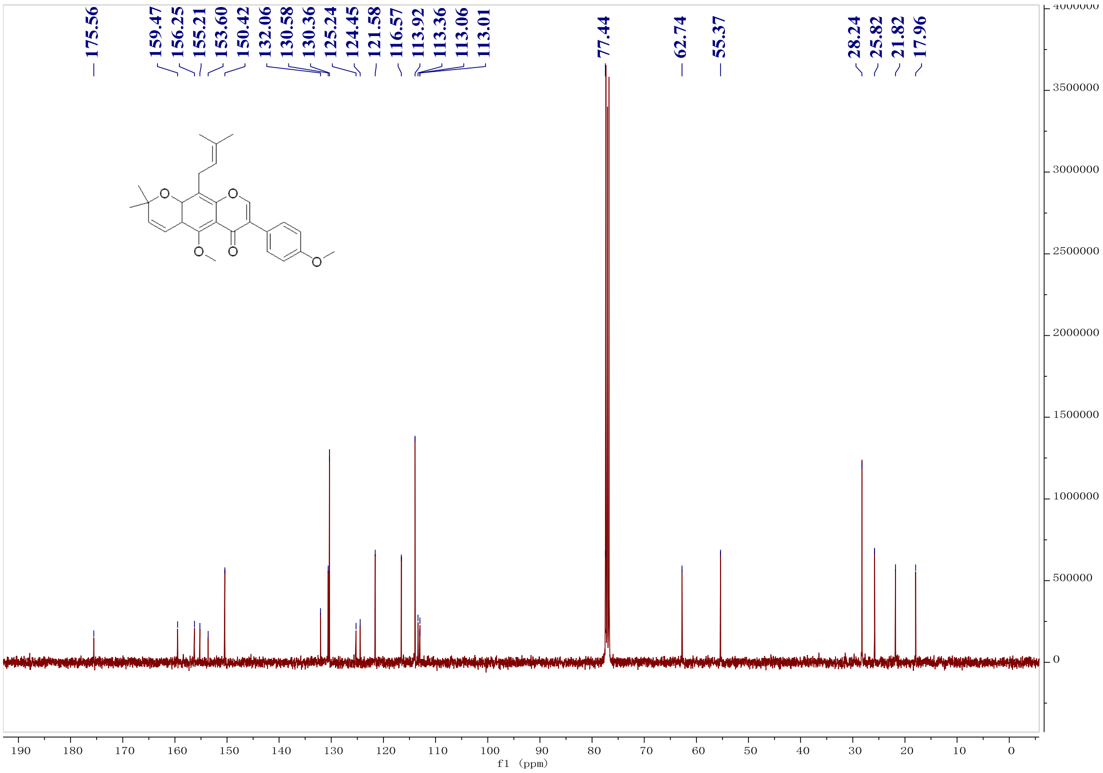

^{1}H NMR of compound 28

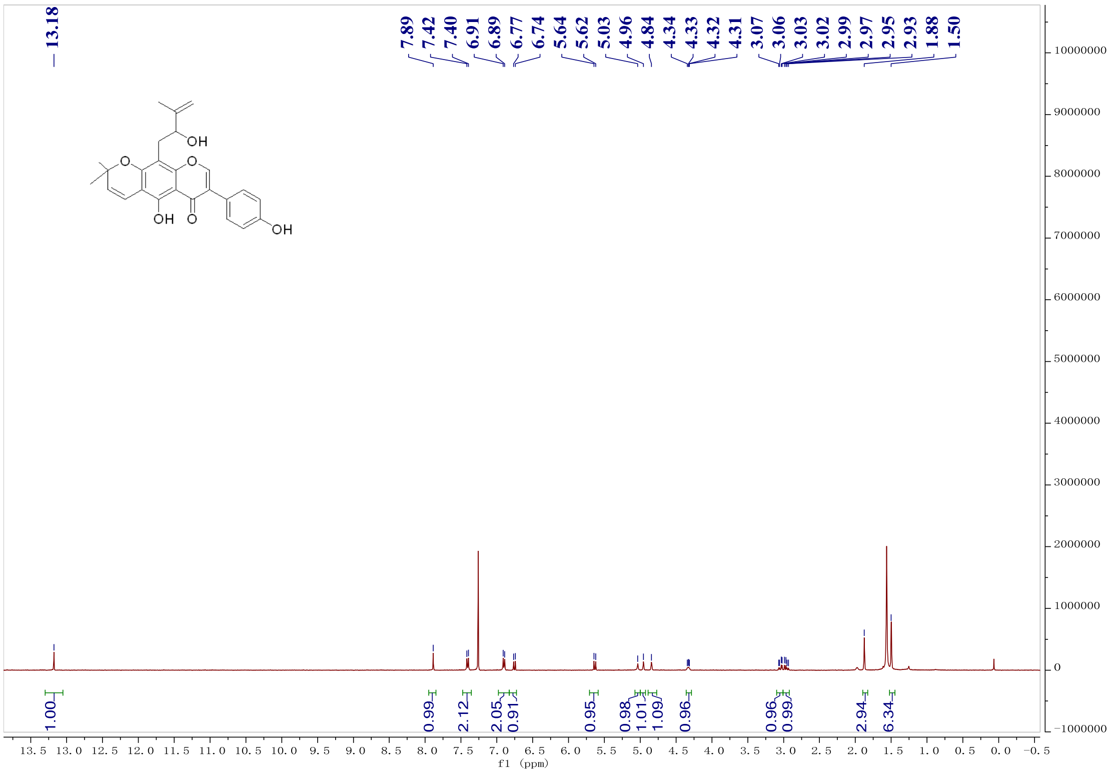

^{13}C NMR of compound 28

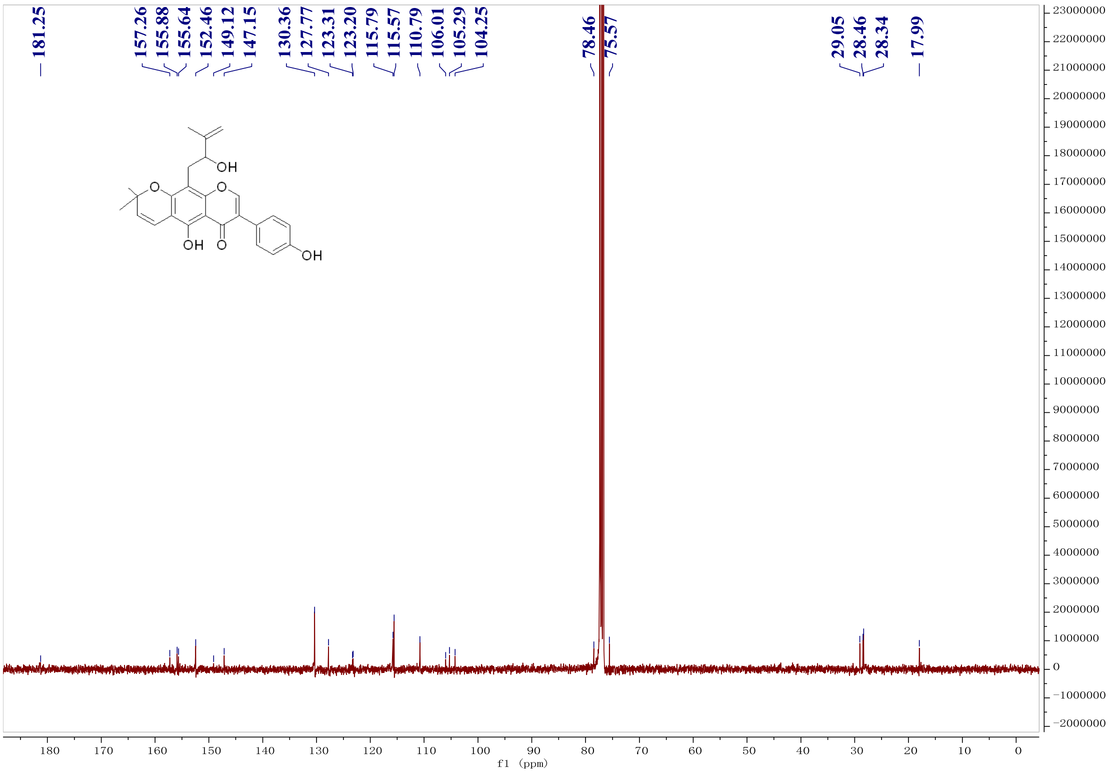

^{1}H NMR of compound 29

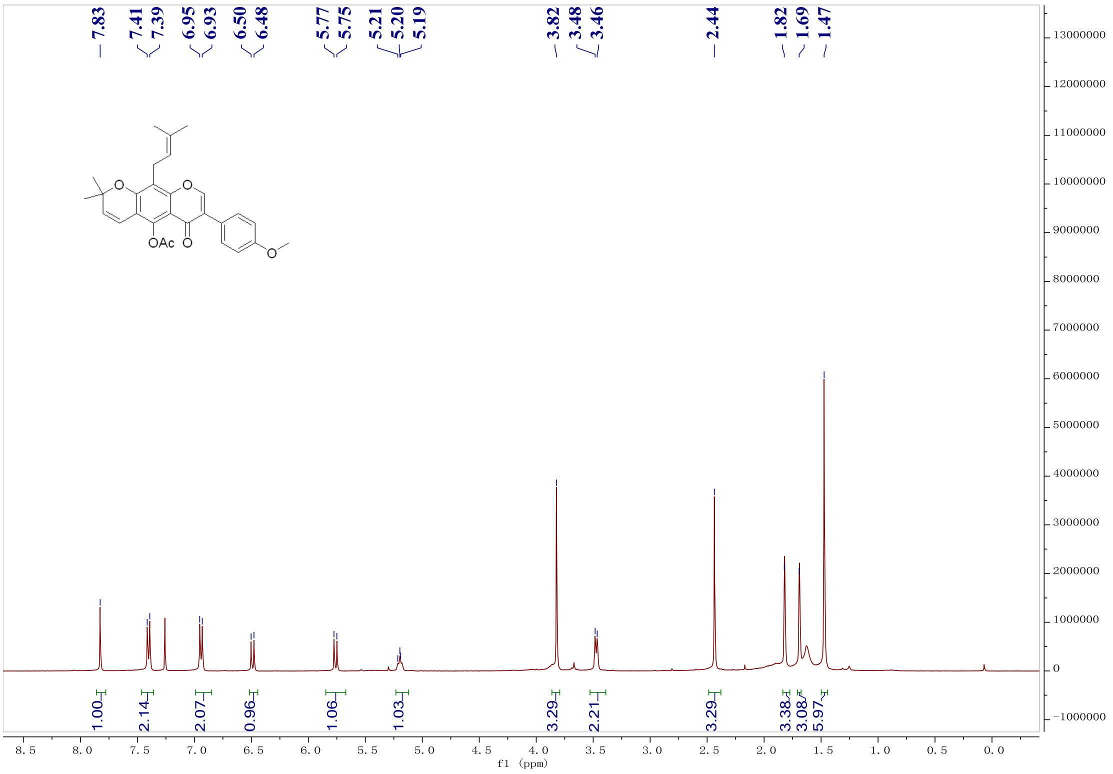

^{13}C NMR of compound 29

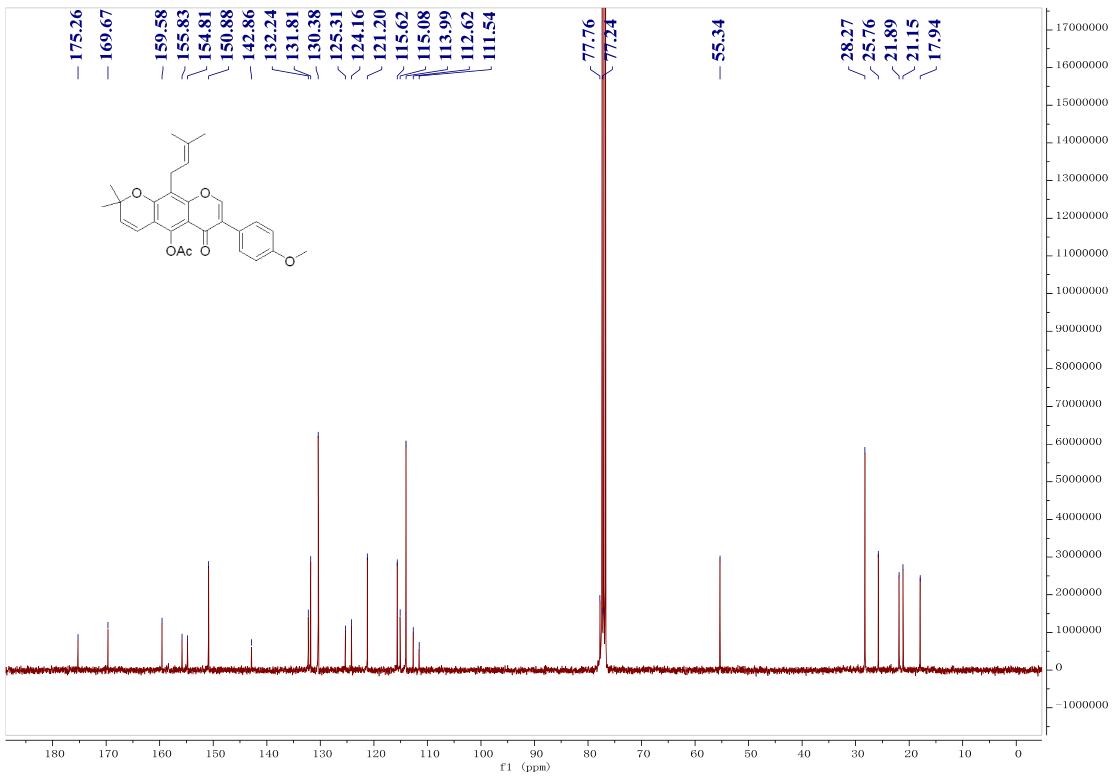

^{1}H NMR of compound 30

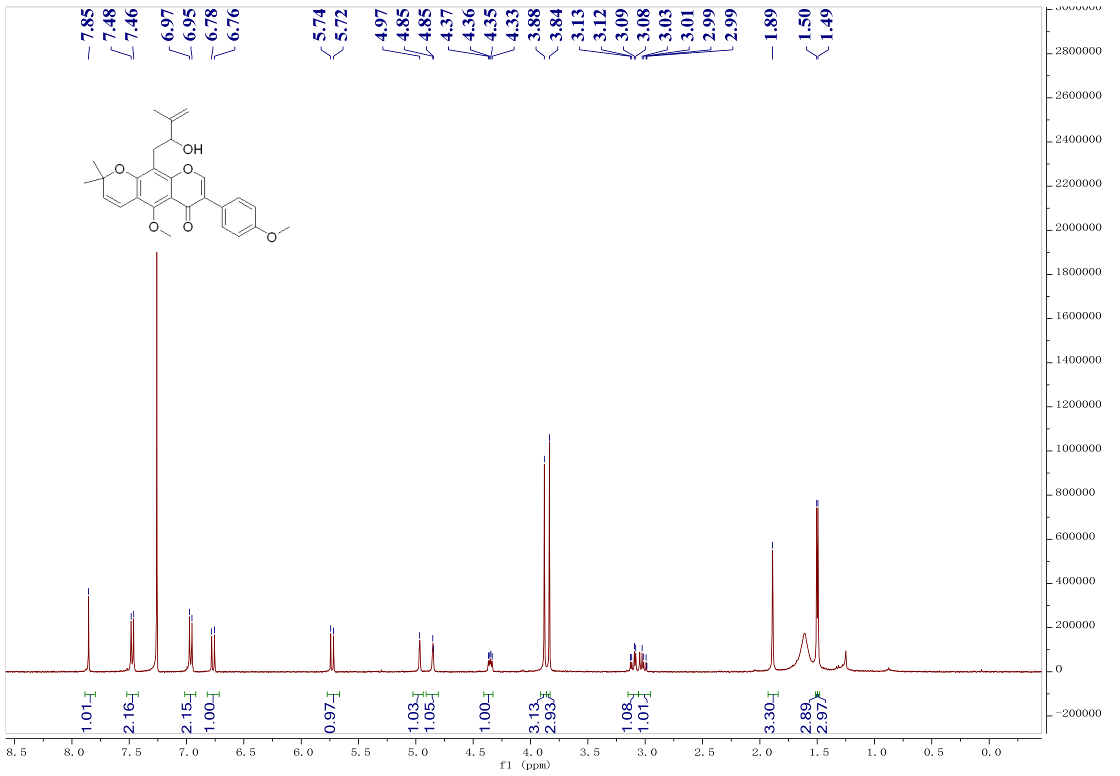

^{1}H NMR of compound 16

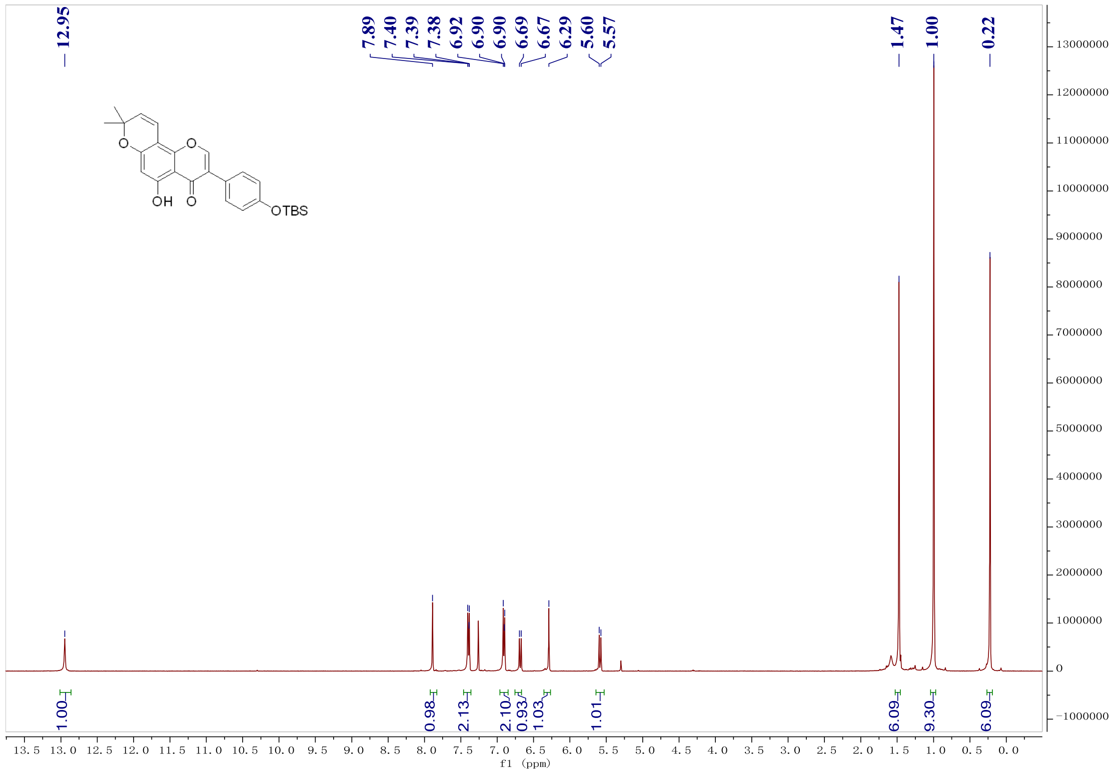

^{13}C NMR of compound 16

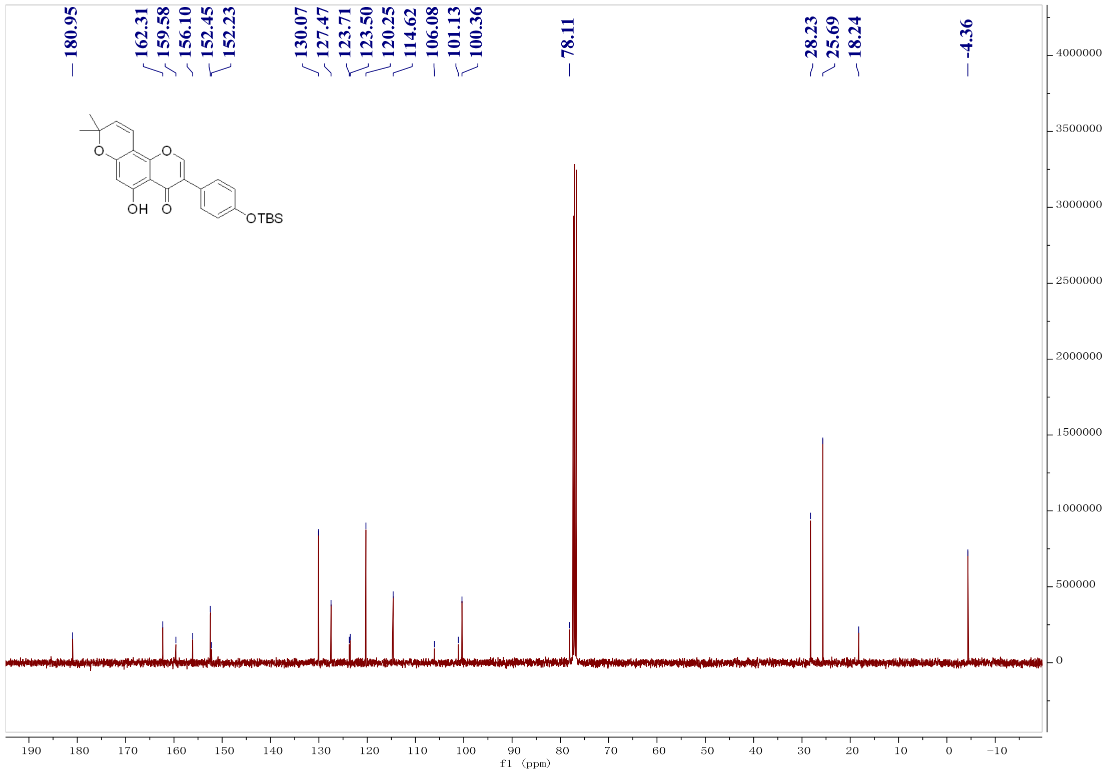

^{1}H NMR of compound 18

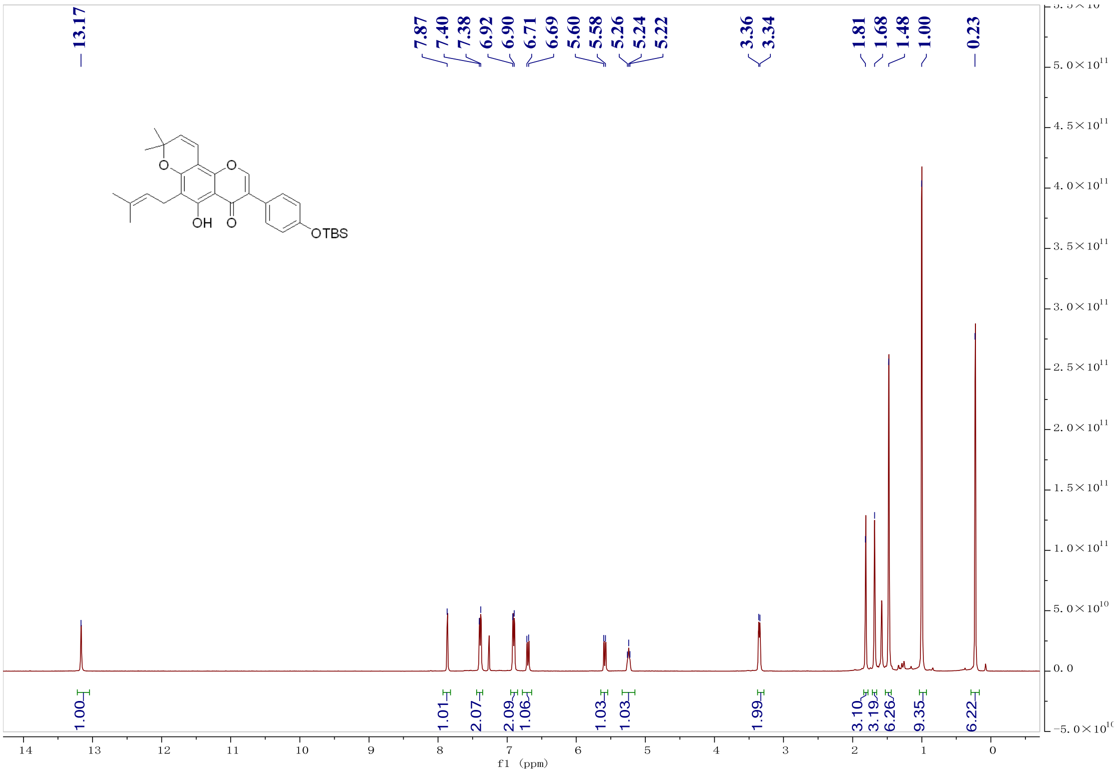

^{13}C NMR of compound 18

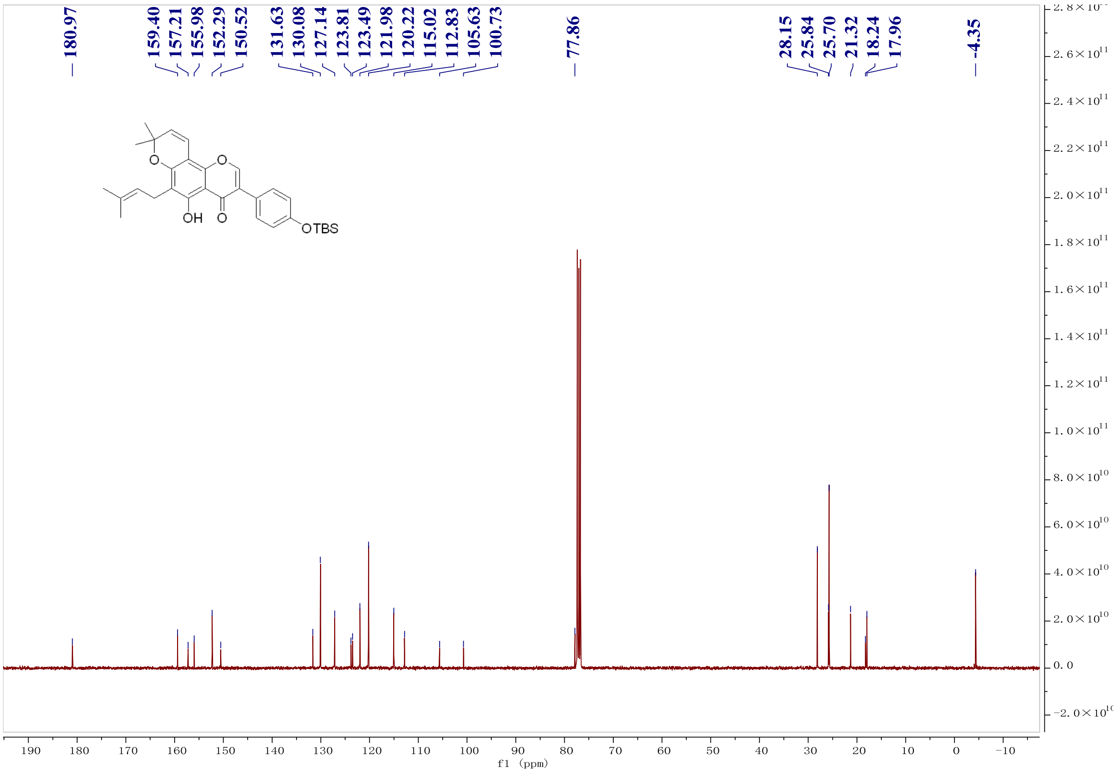

^{1}H NMR of compound 22

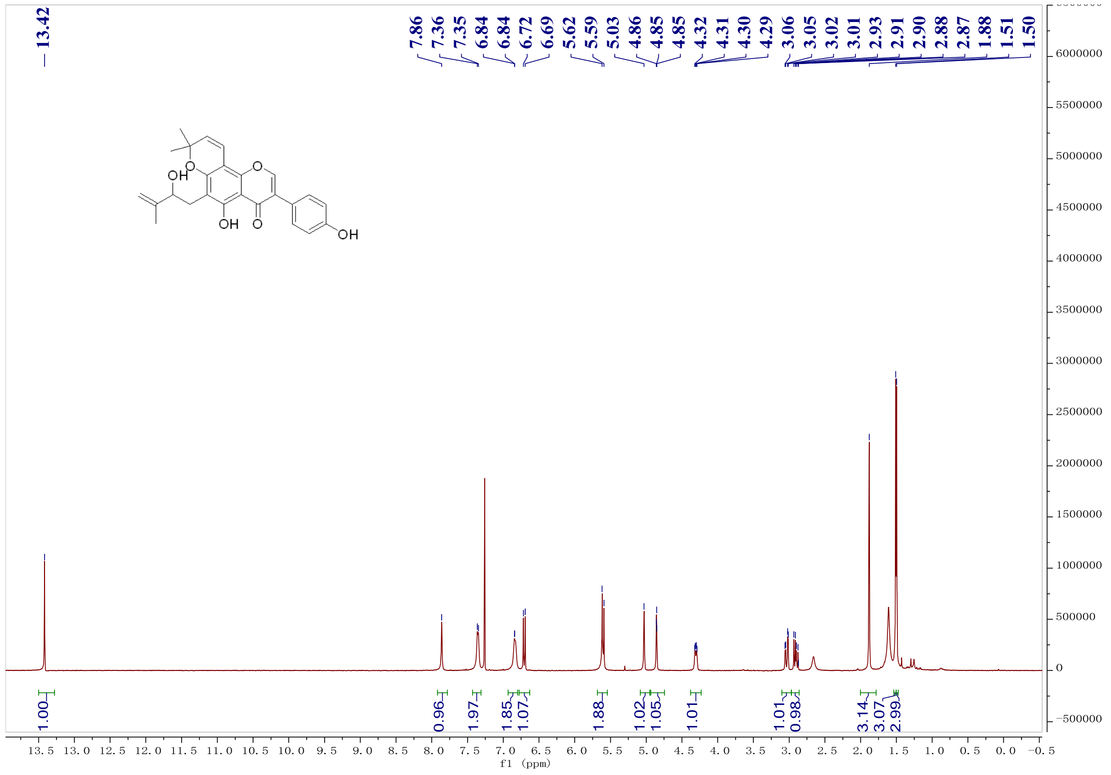

^{13}C NMR of compound 22

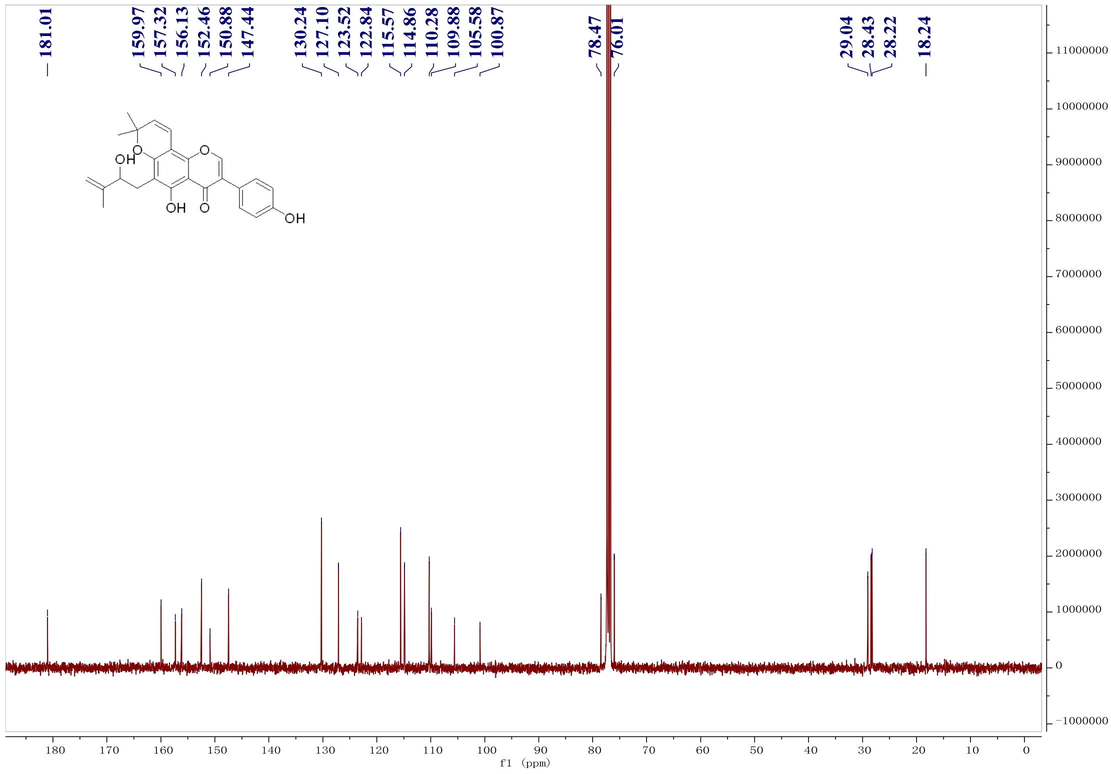

^{1}H NMR of compound 19

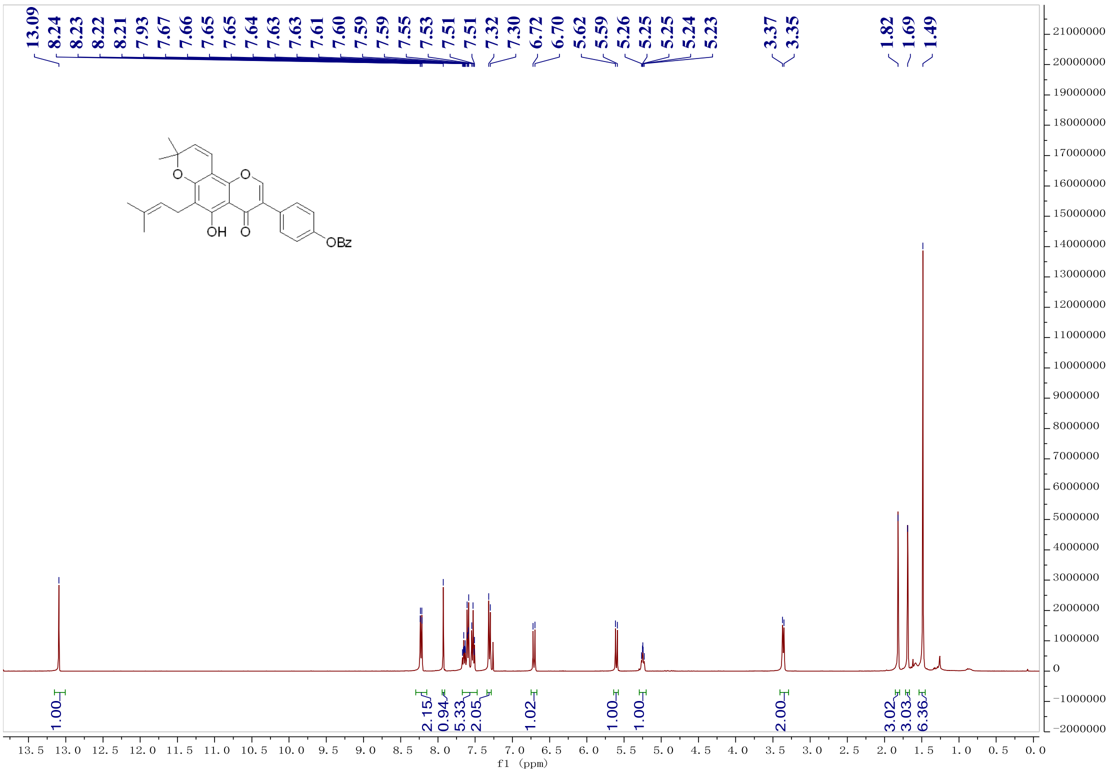

^{13}C NMR of compound 19

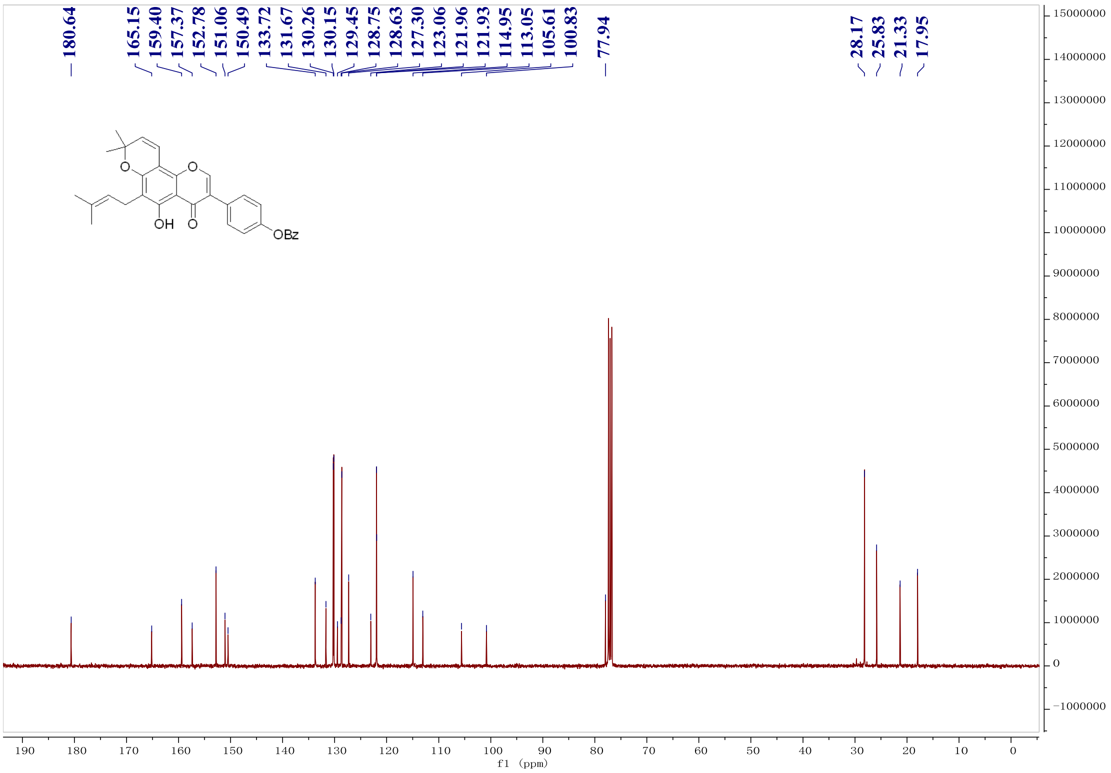

^{1}H NMR of compound 20

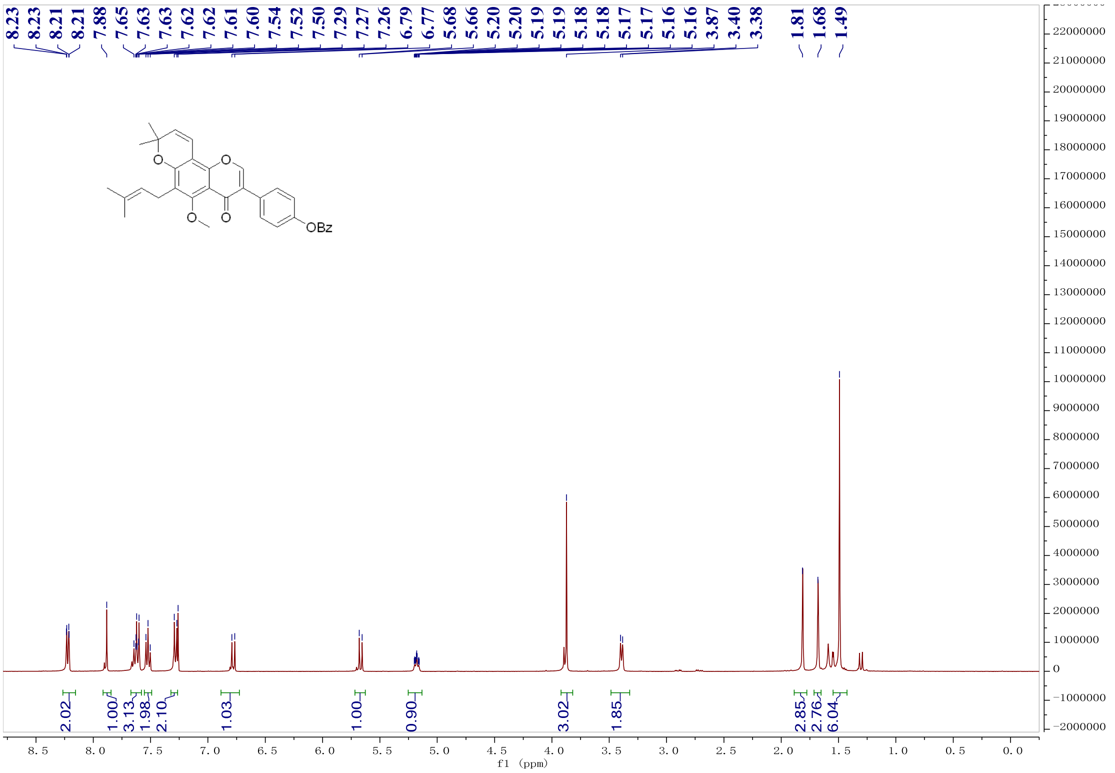

^{13}C NMR of compound 20

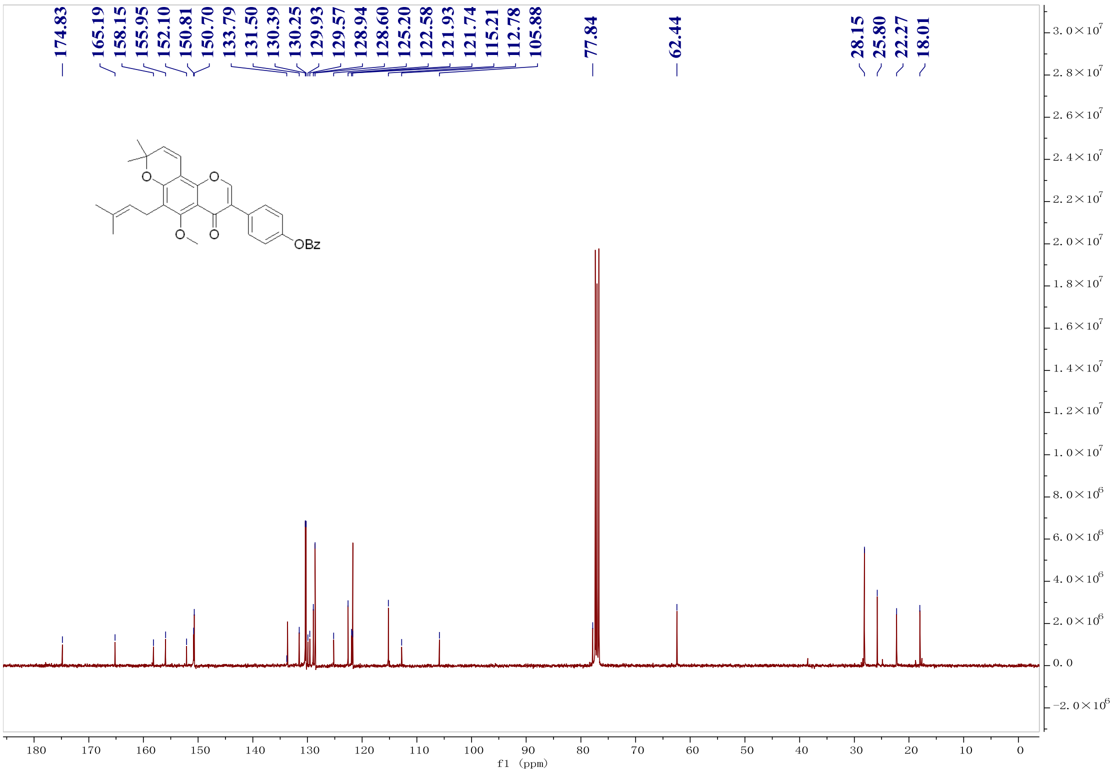

^{1}H NMR of compound 21

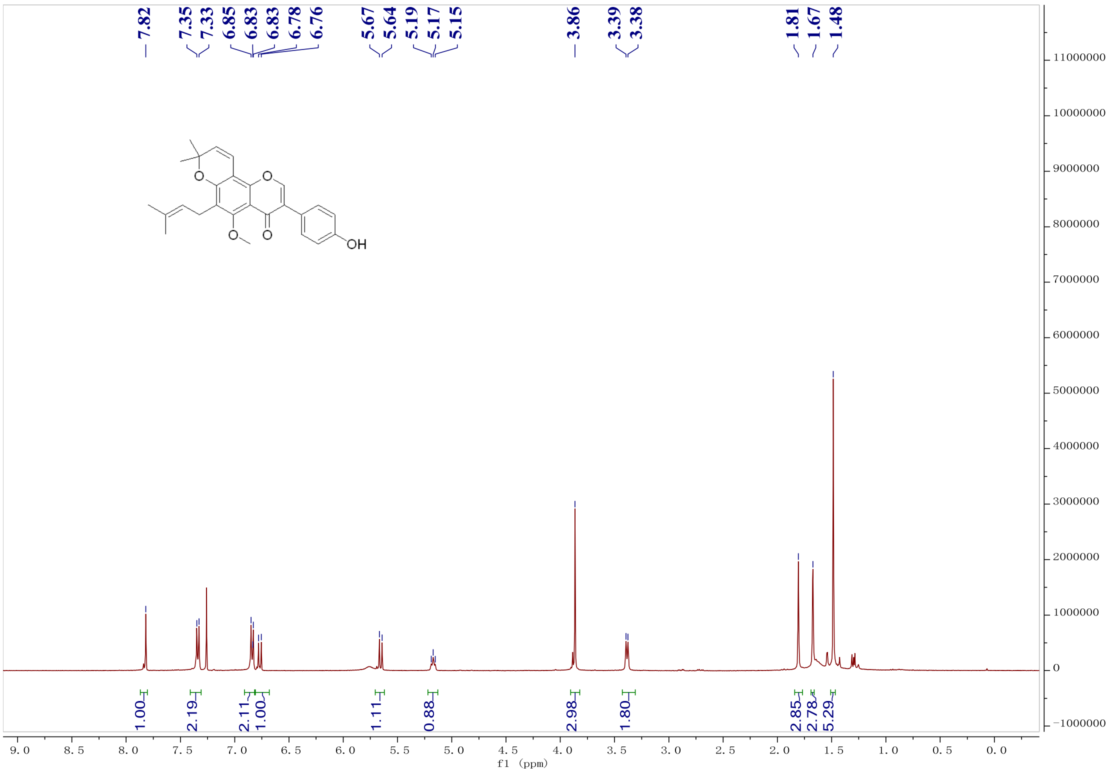

^{1}H NMR of compound **32**

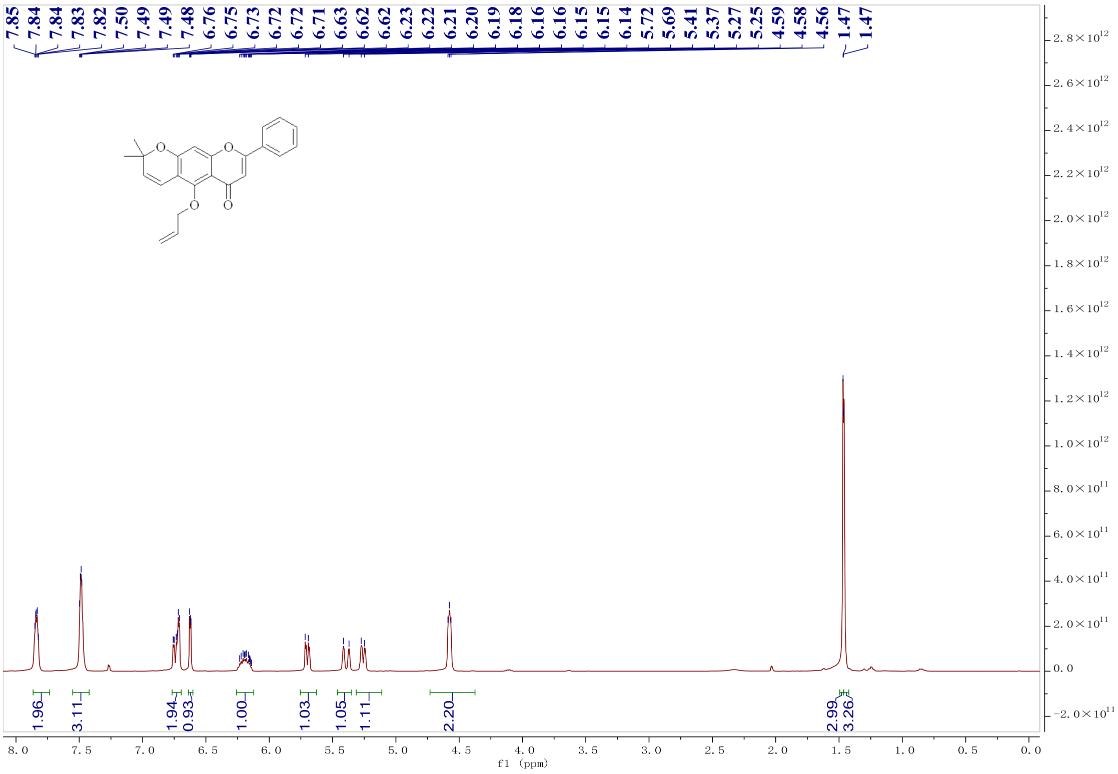

^{13}C NMR of compound **32**

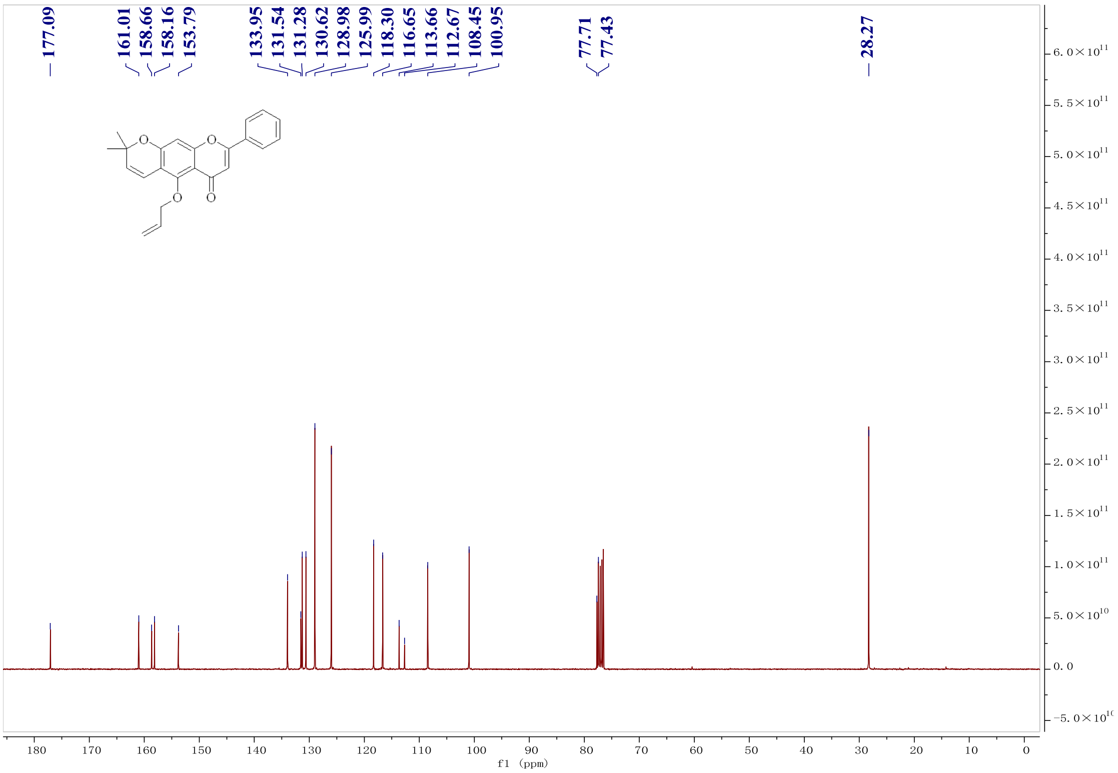

^{1}H NMR of compound **33**

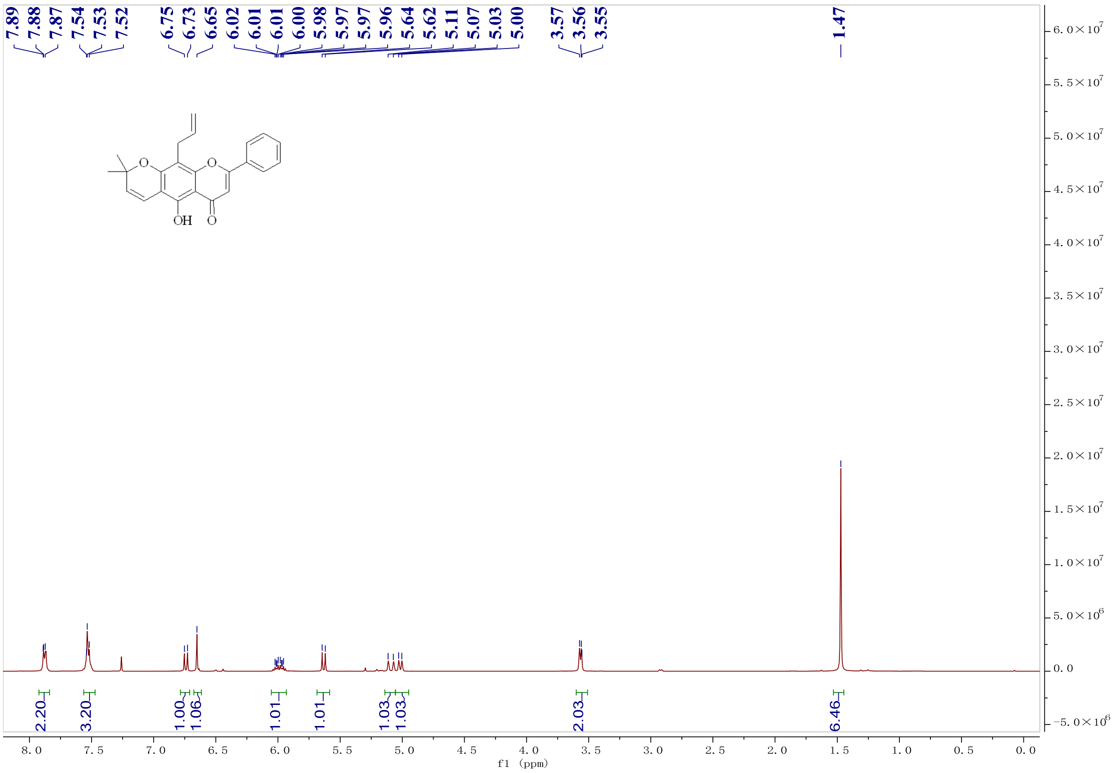

^{13}C NMR of compound **33**

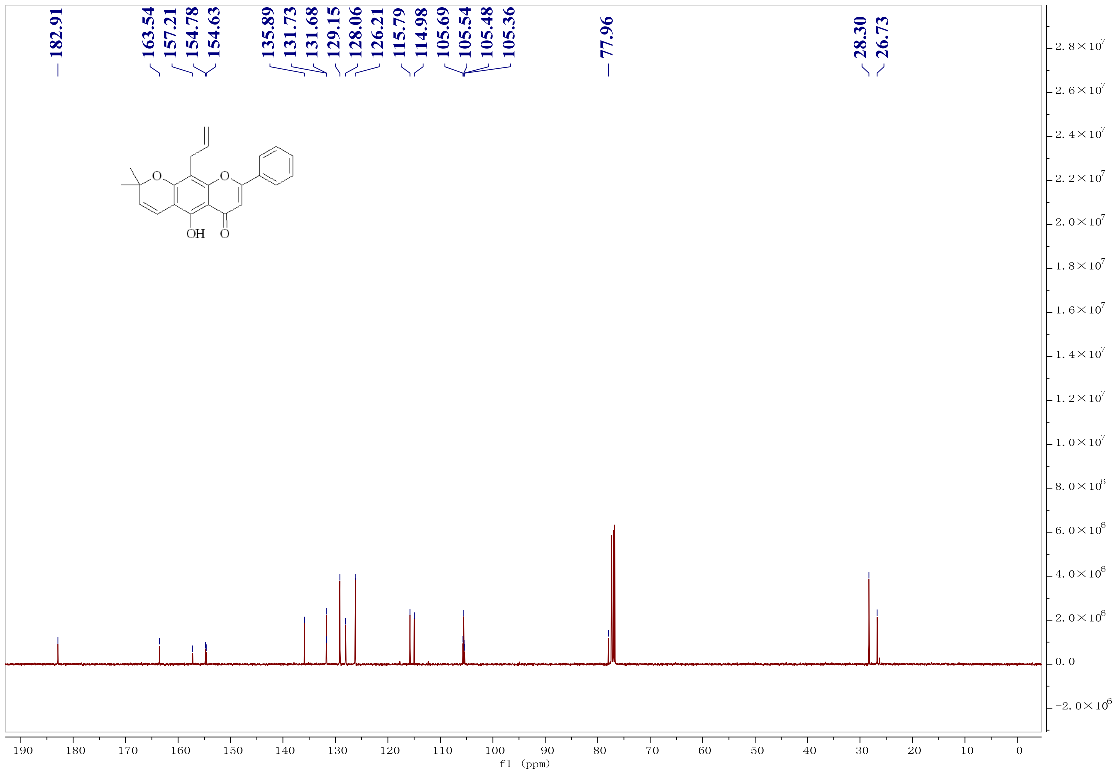

^{1}H NMR of compound **34a**

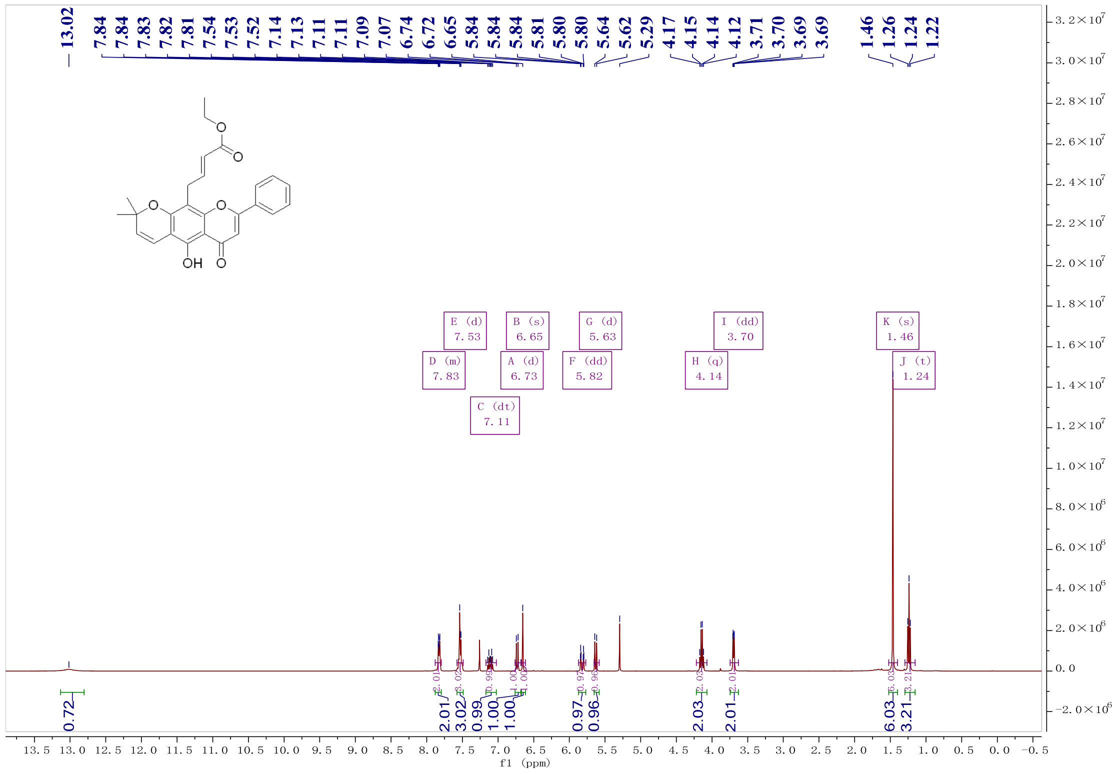

^{13}C NMR of compound **34a**

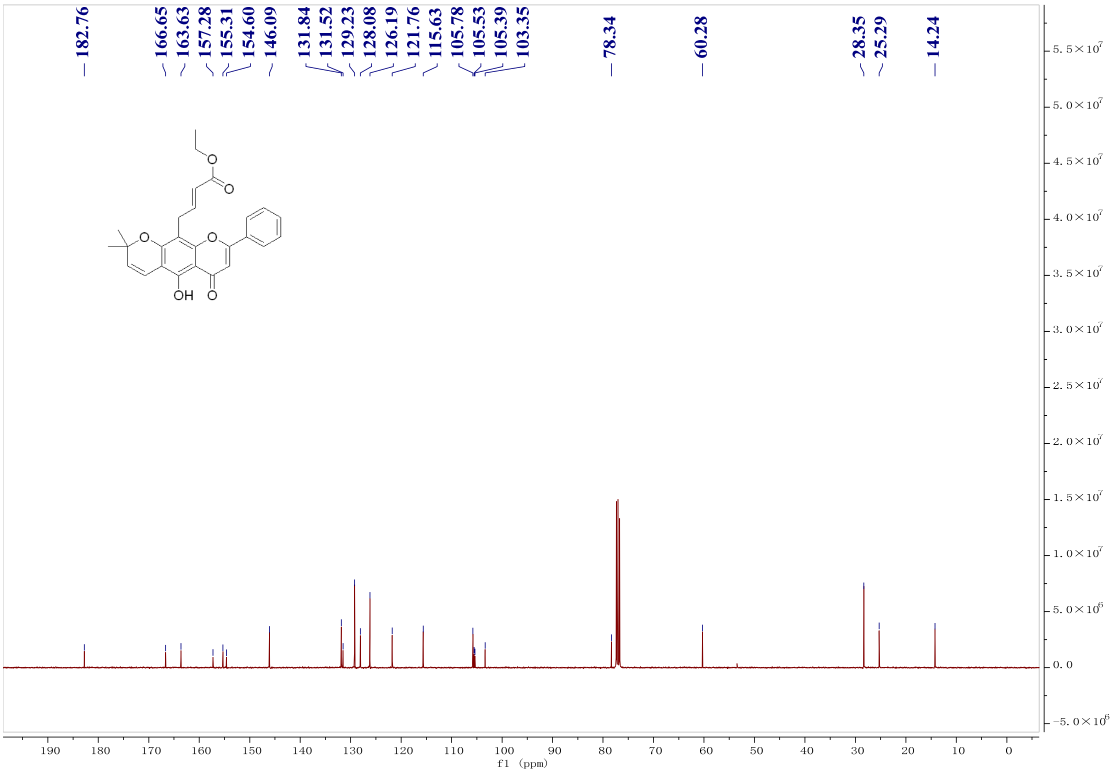

^{1}H NMR of compound **34b**

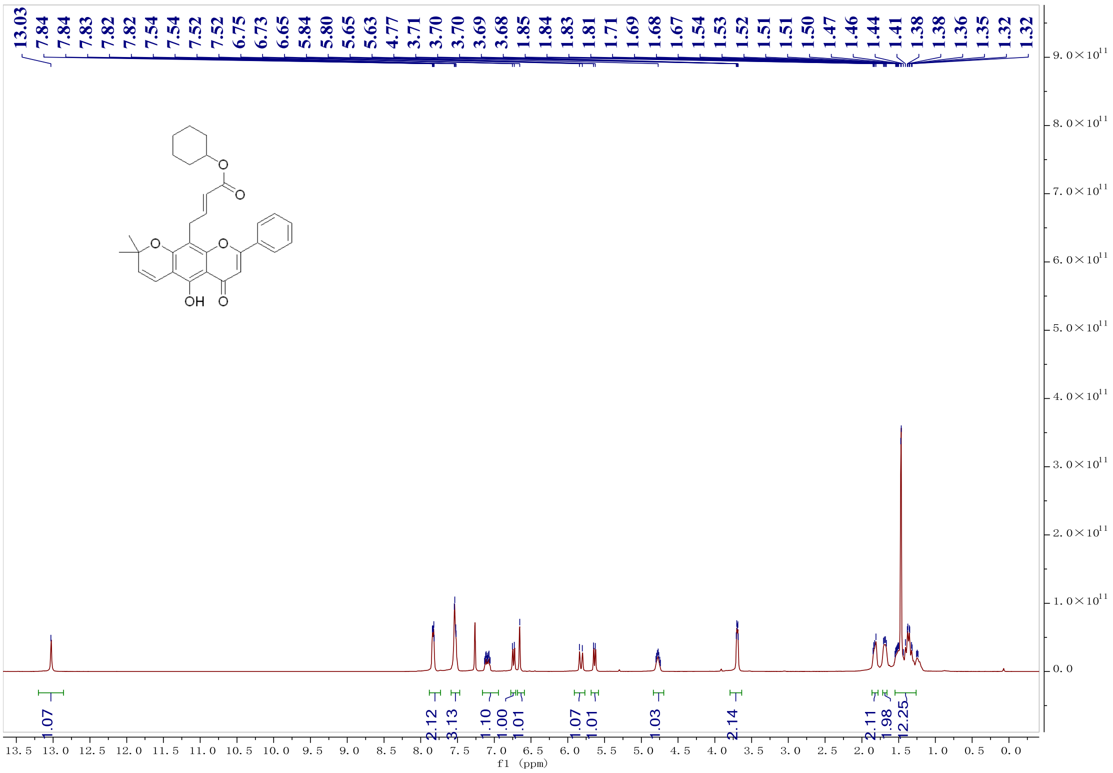

^{13}C NMR of compound **34b**

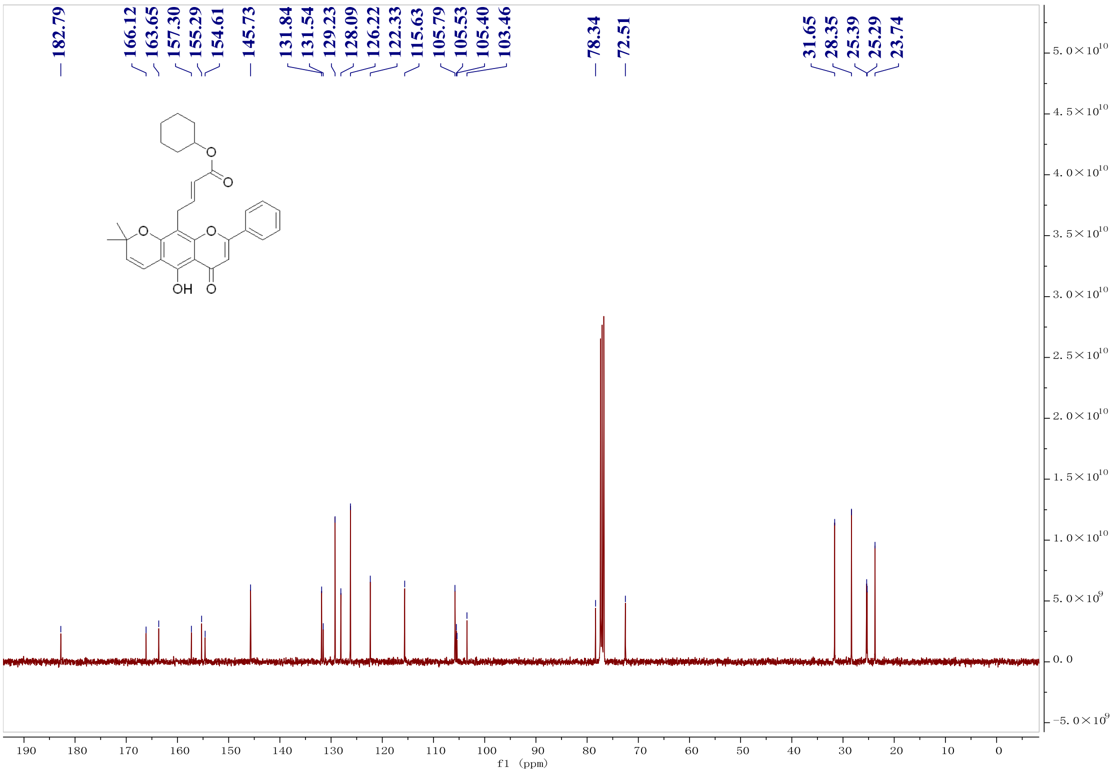

^{1}H NMR of compound **34c**

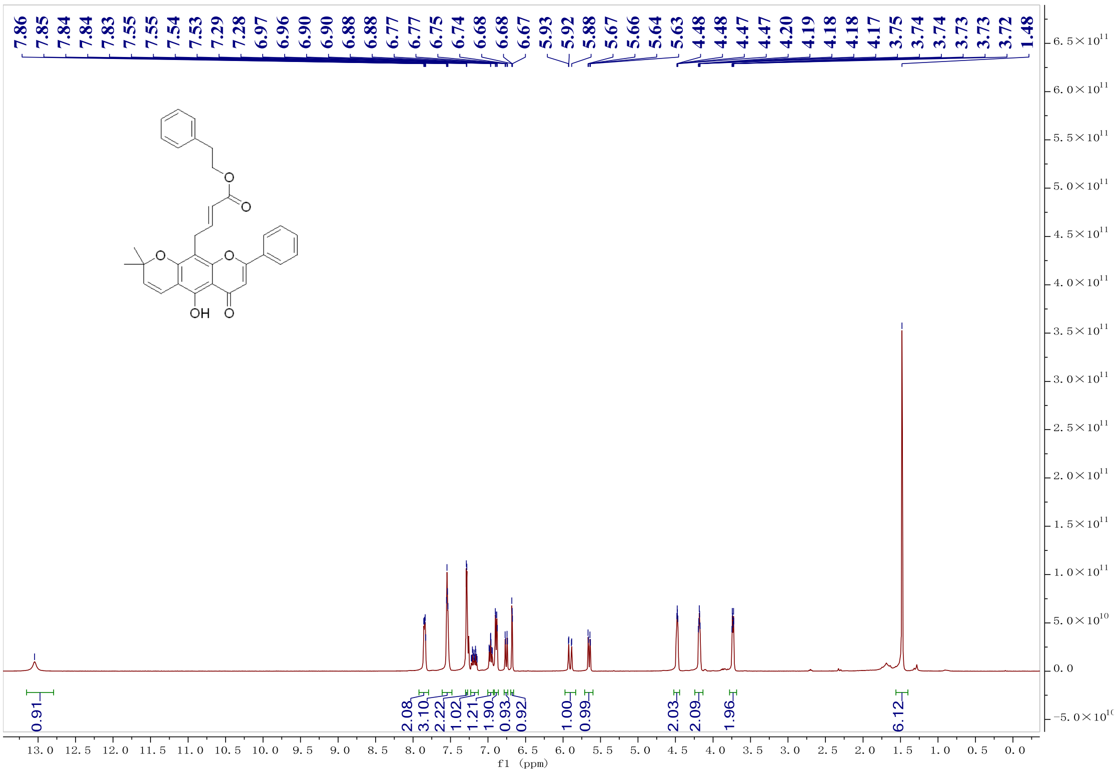

^{13}C NMR of compound **34c**

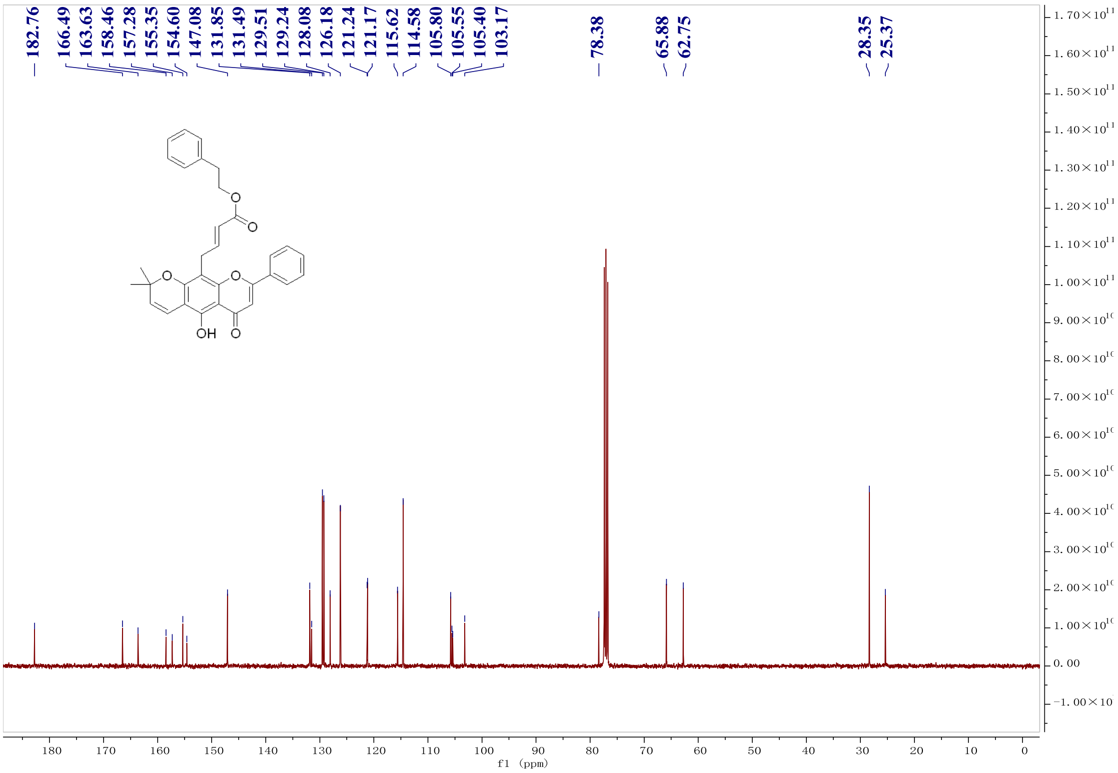

^{1}H NMR of compound **34d**

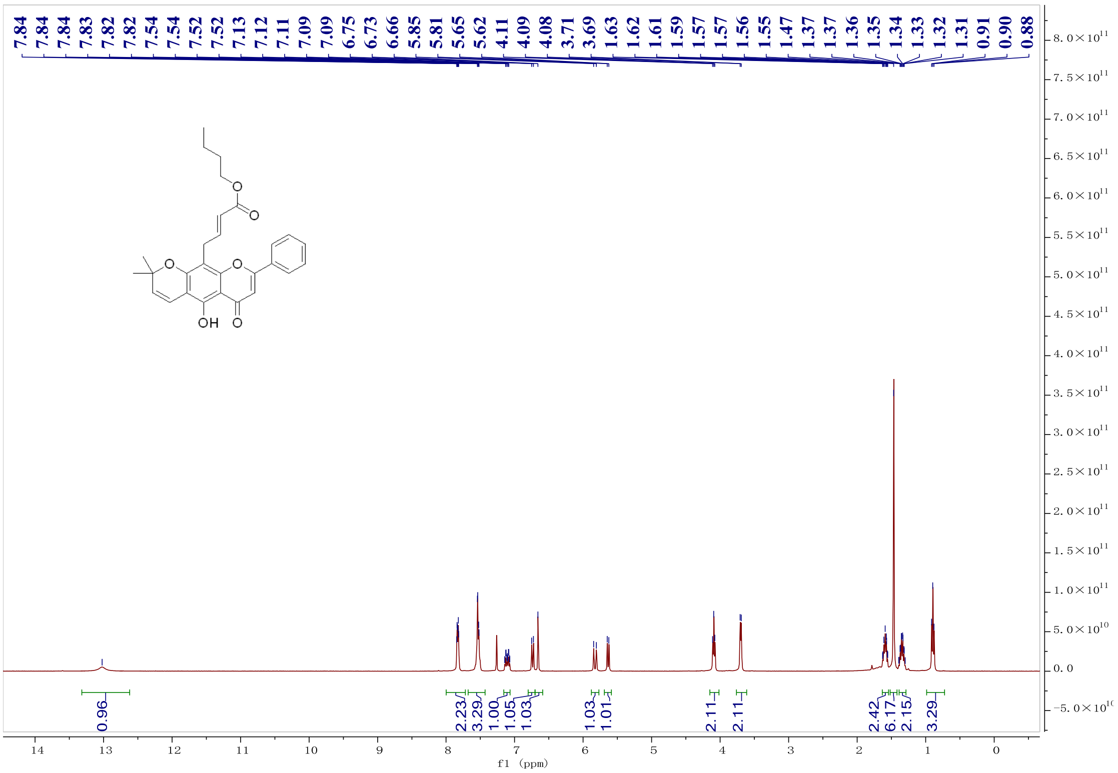

^{13}C NMR of compound **34d**

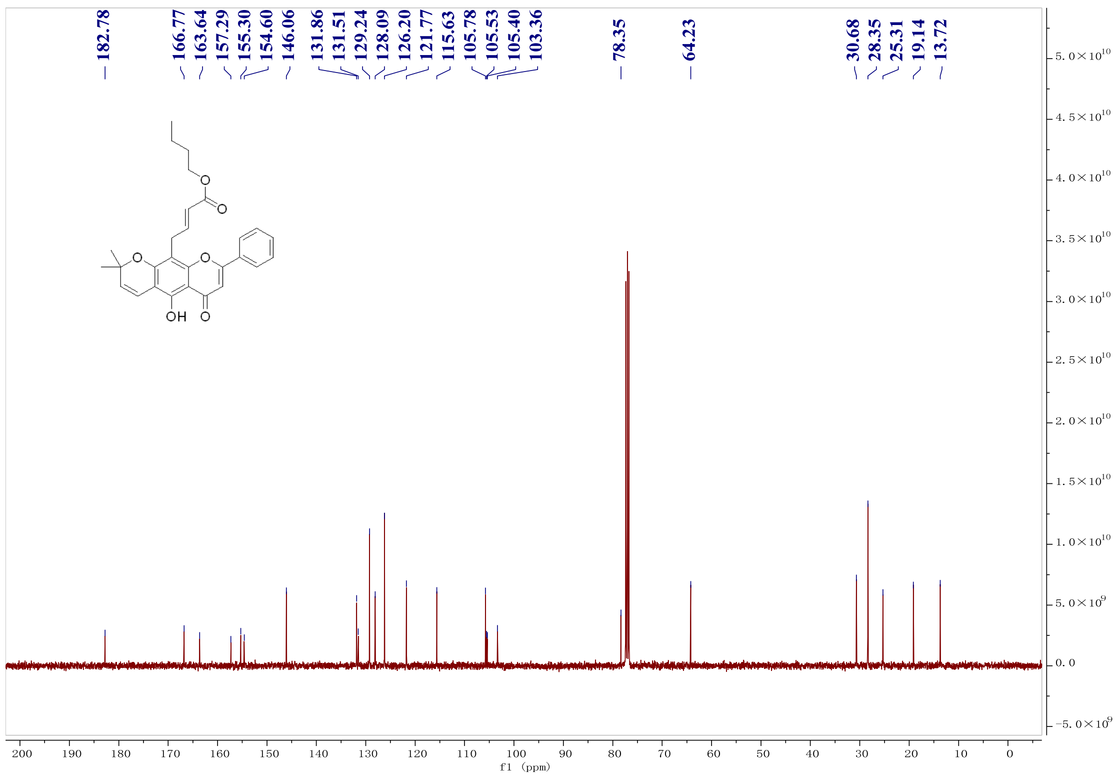

^{1}H NMR of compound **34e**

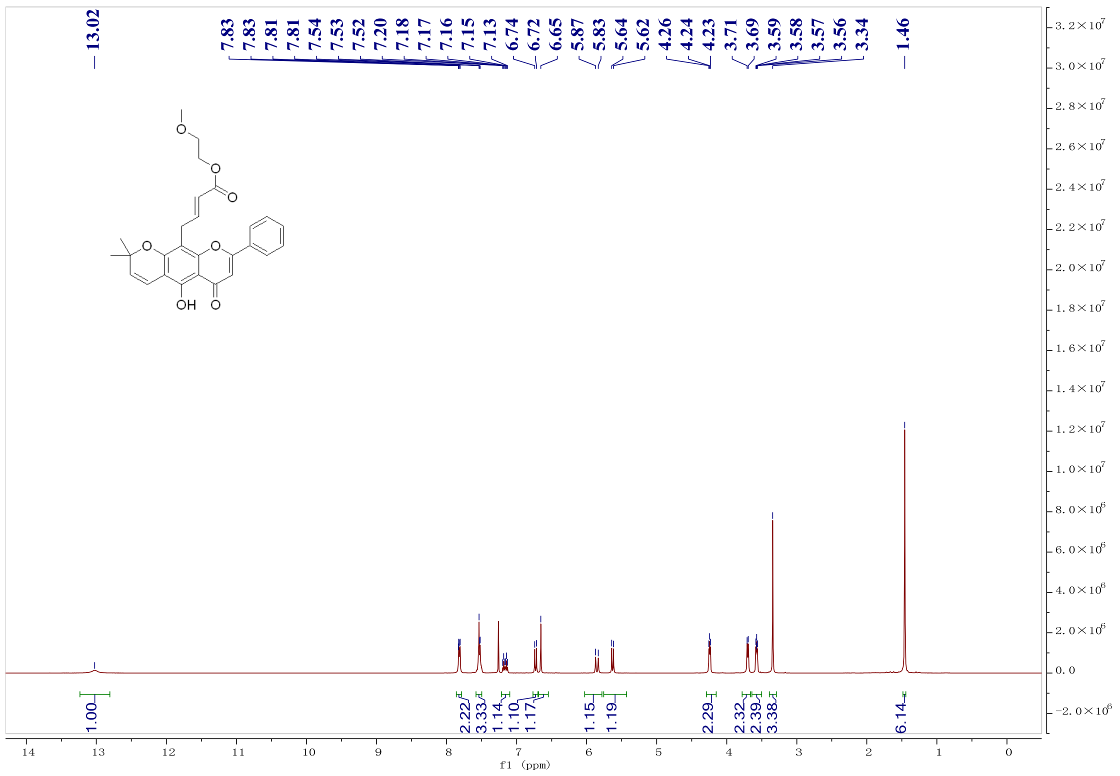

^{13}C NMR of compound **34e**

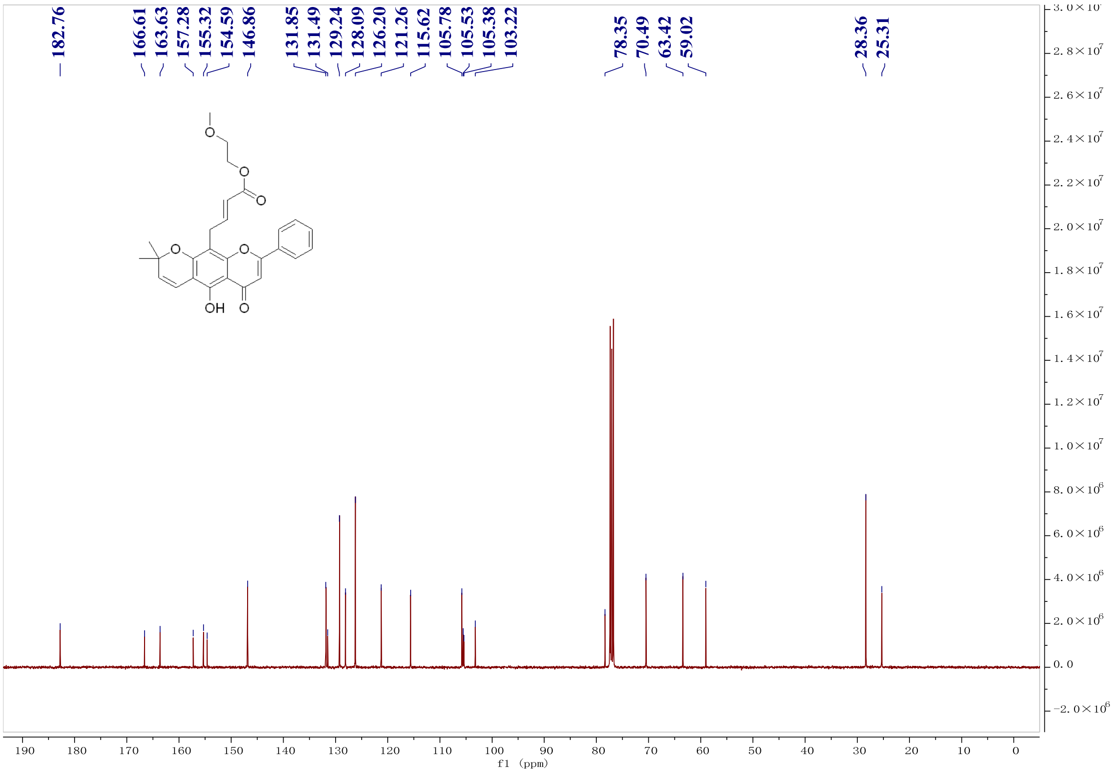

^{1}H NMR of compound **34f**

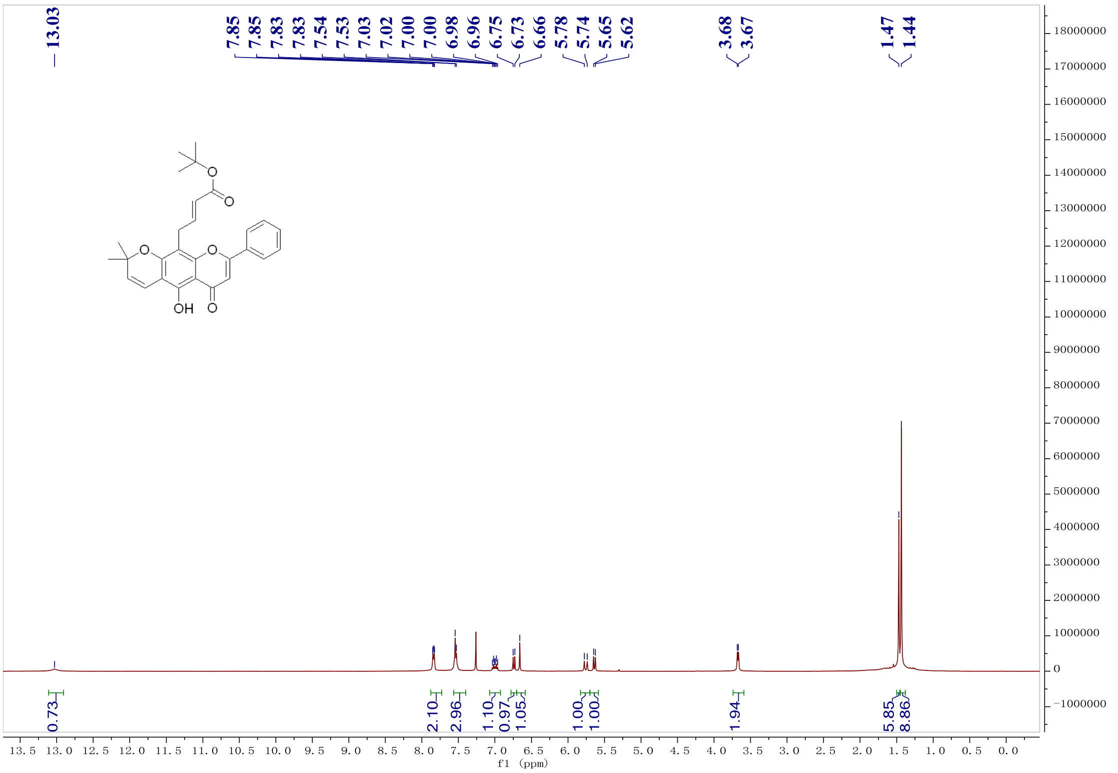

^{13}C NMR of compound **34f**

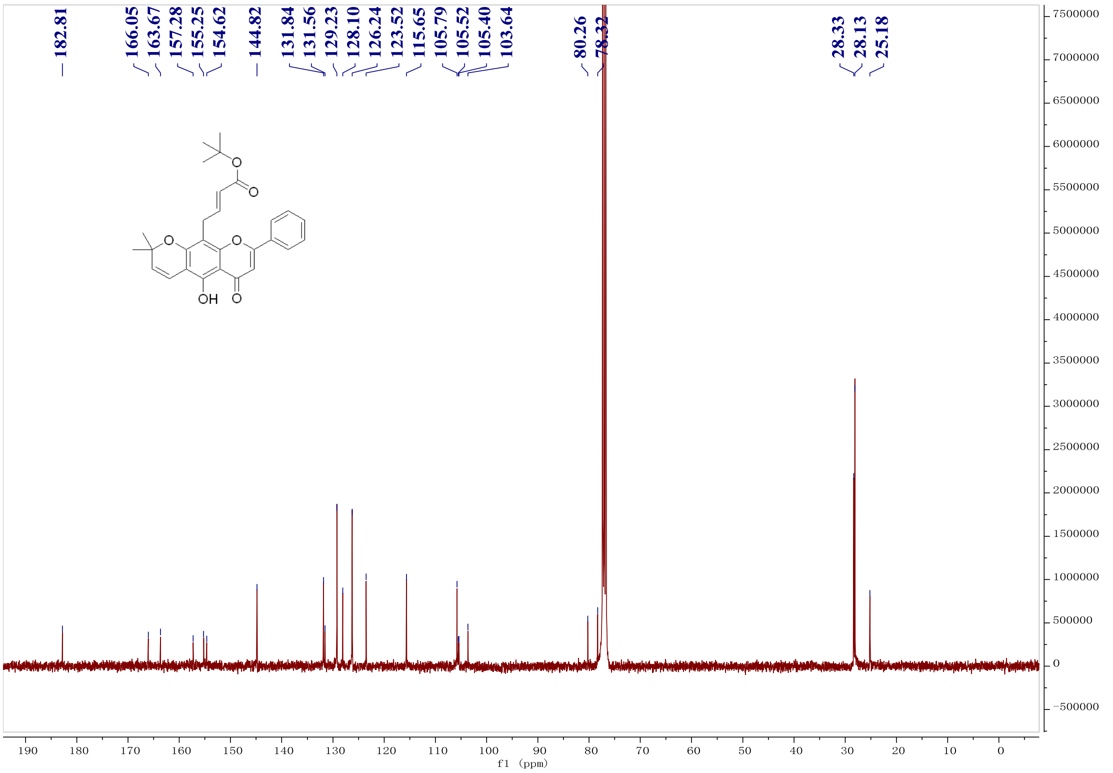

^{1}H NMR of compound **36**

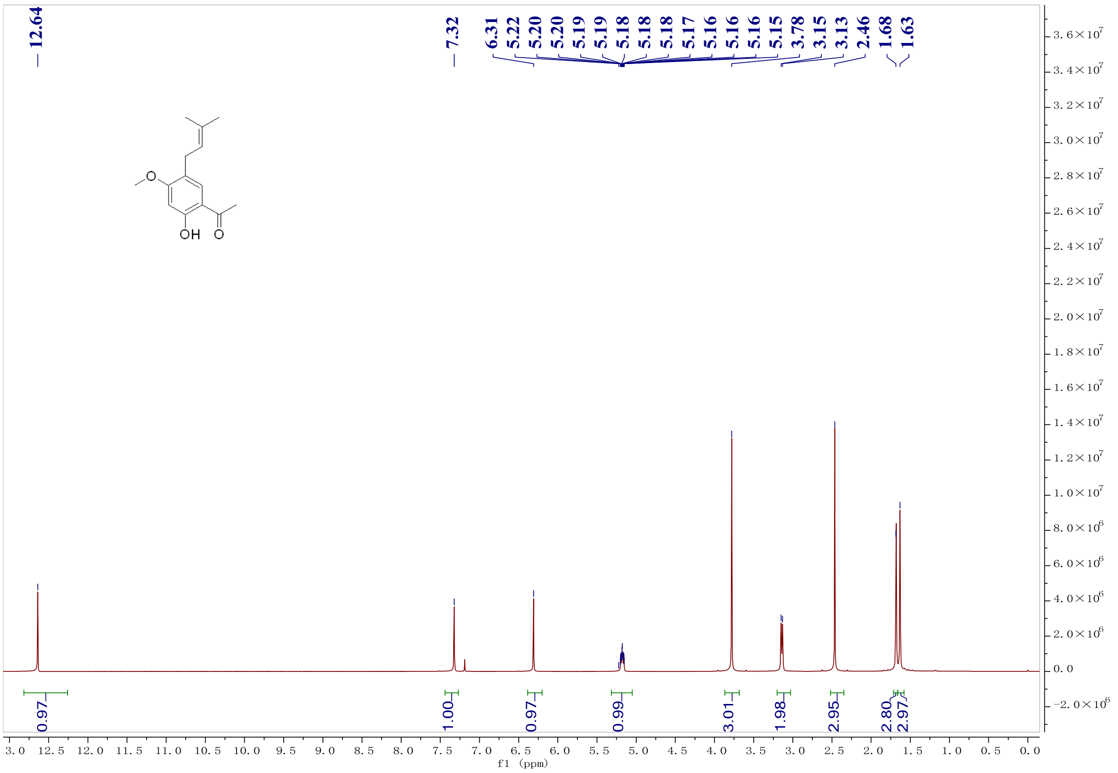

^{13}C NMR of compound **36**

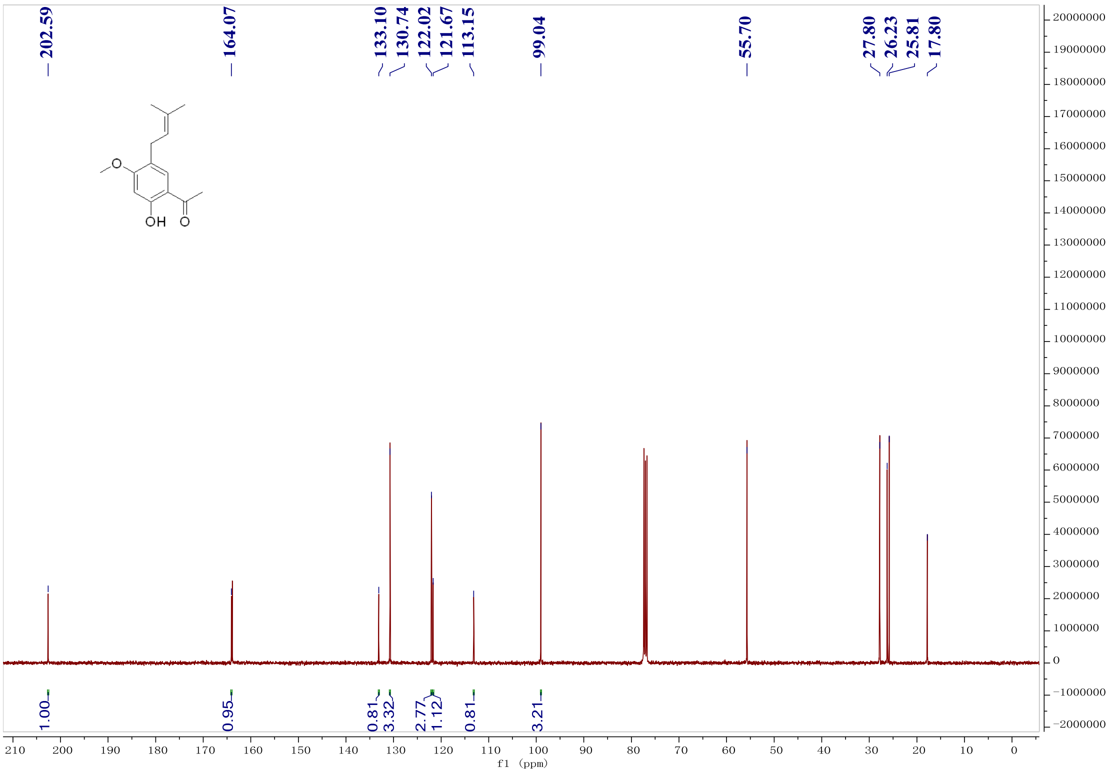

^{1}H NMR of compound **38a**

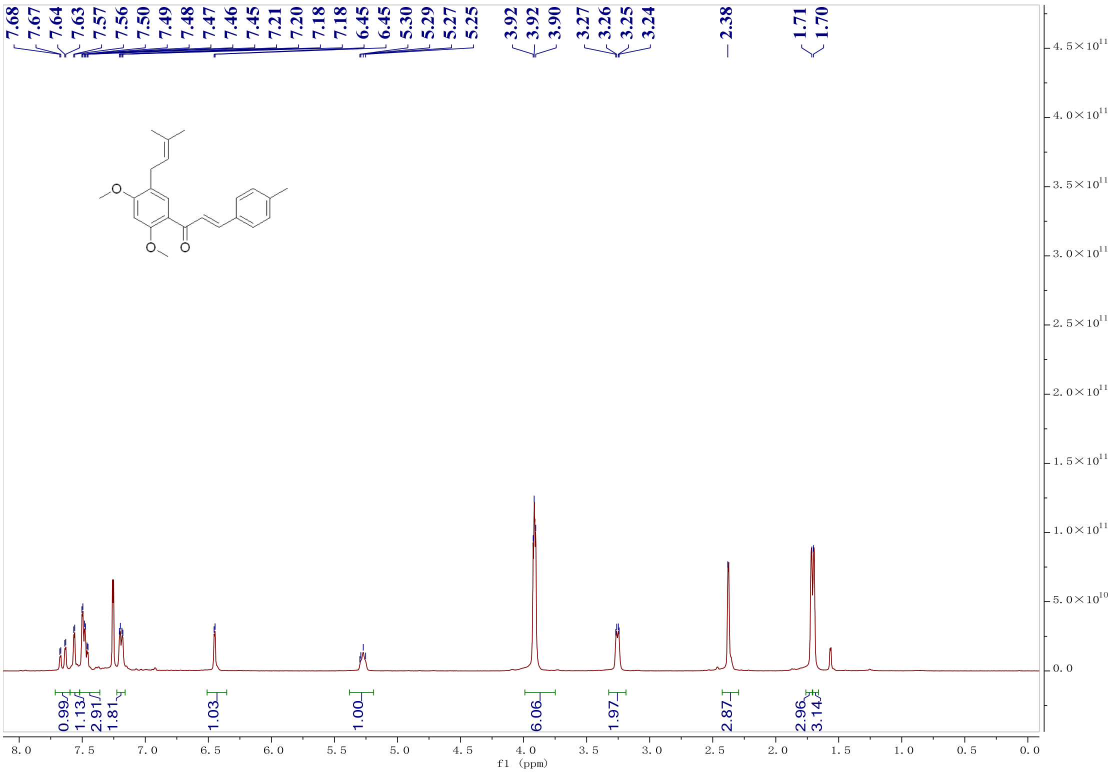

^{13}C NMR of compound **38a**

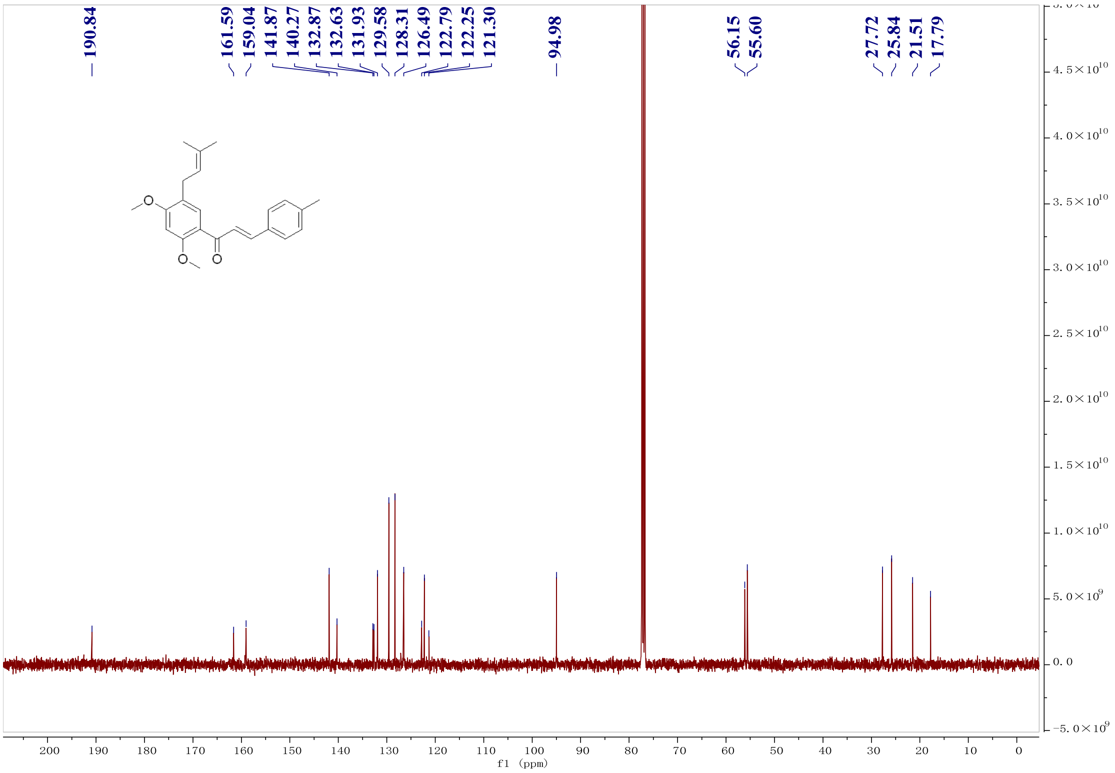

^{1}H NMR of compound **38b**

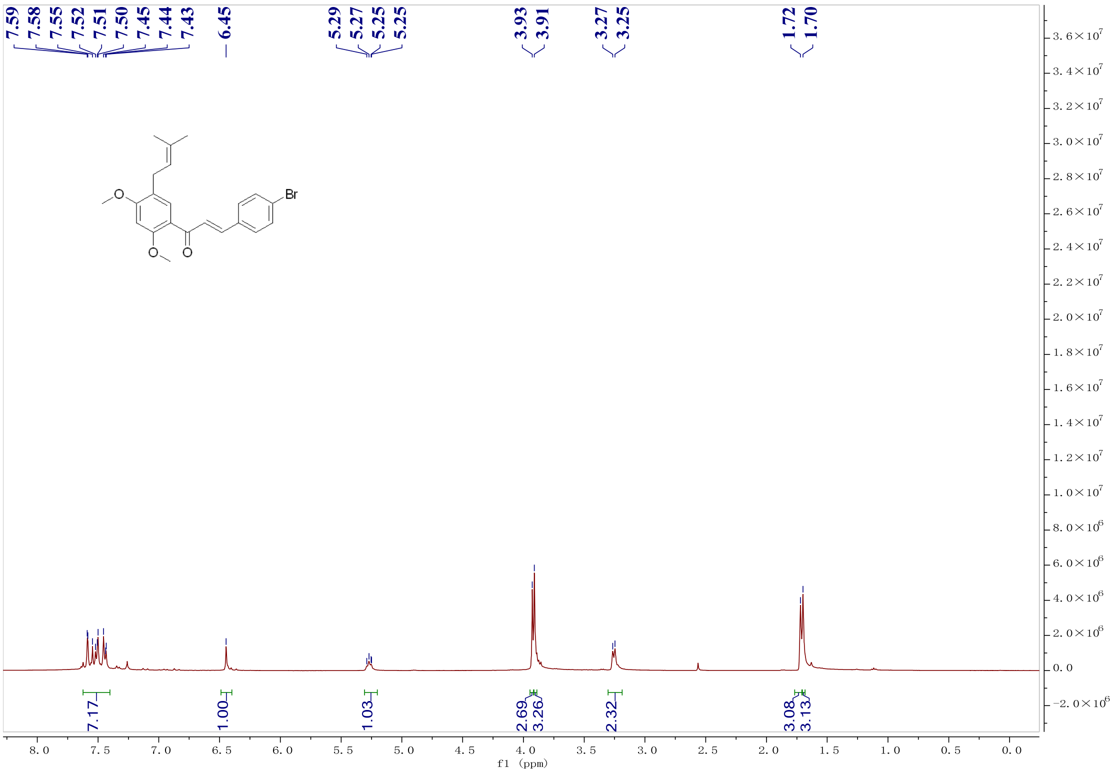

^{13}C NMR of compound **38b**

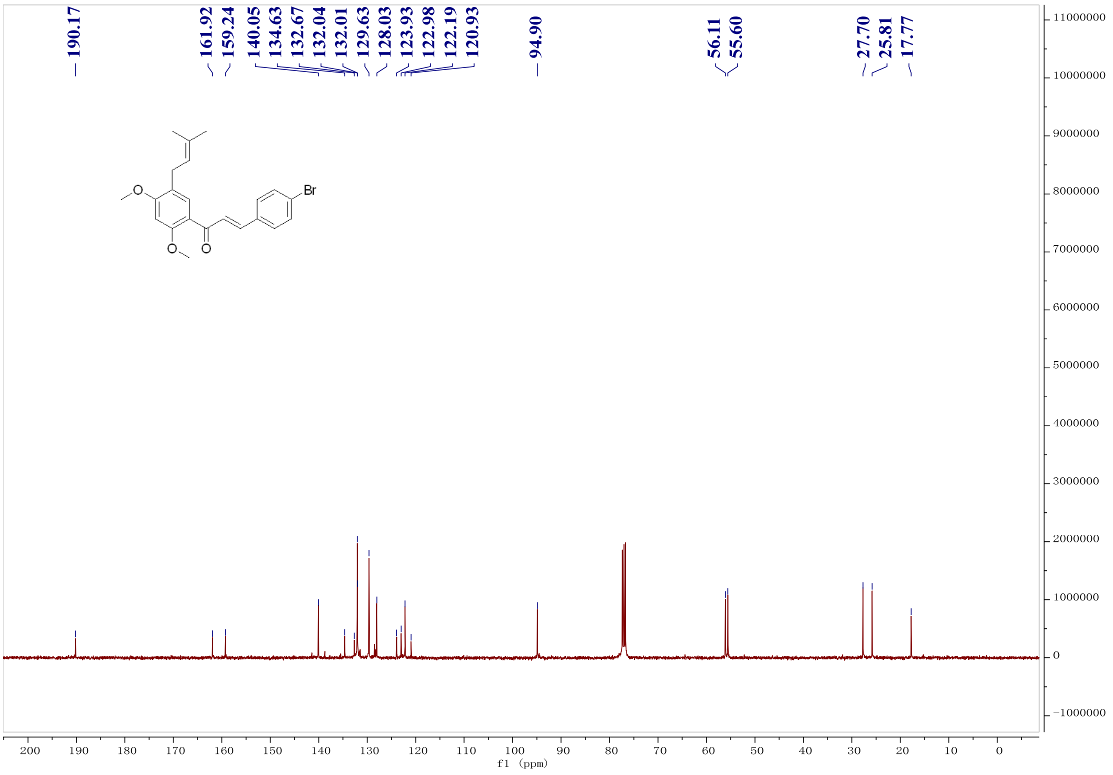

^{1}H NMR of compound **38c**

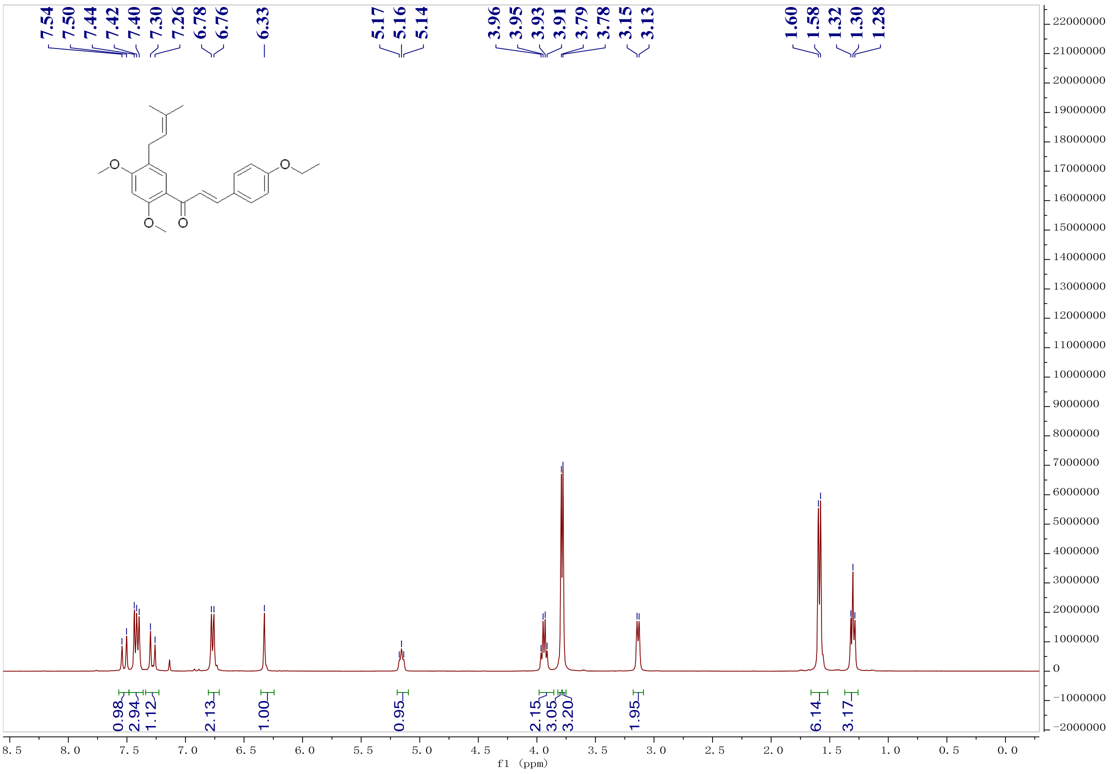

^{13}C NMR of compound **38c**

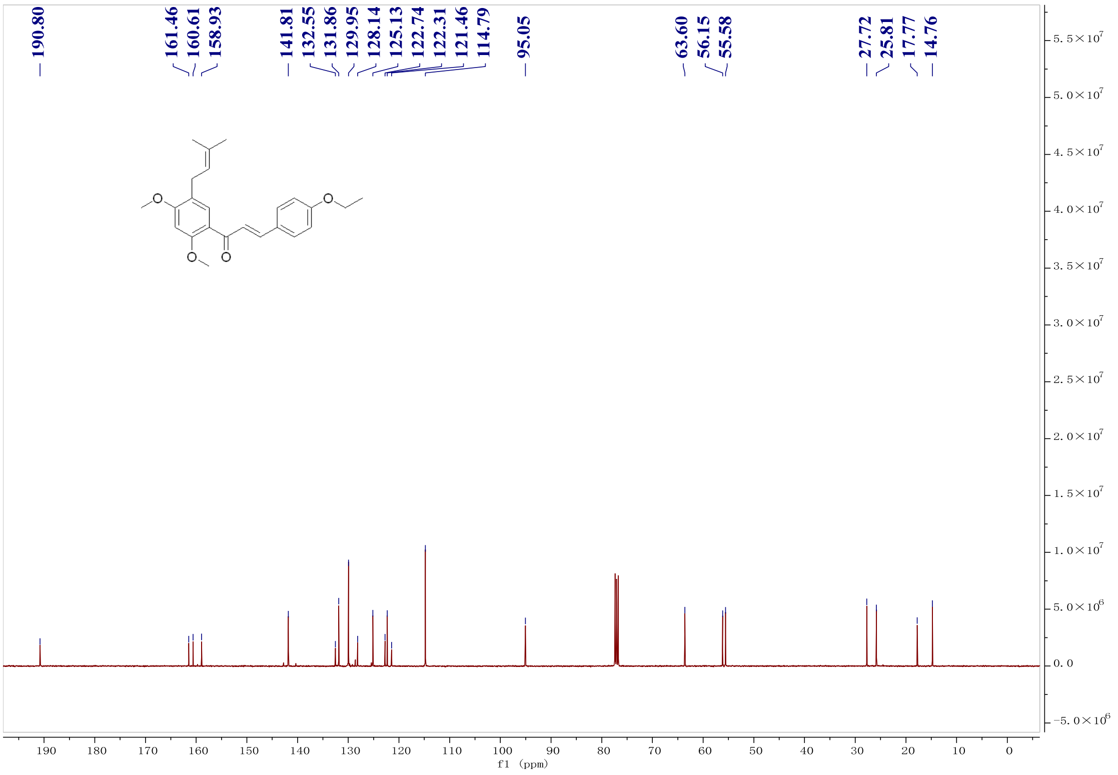

^{1}H NMR of compound **38d**

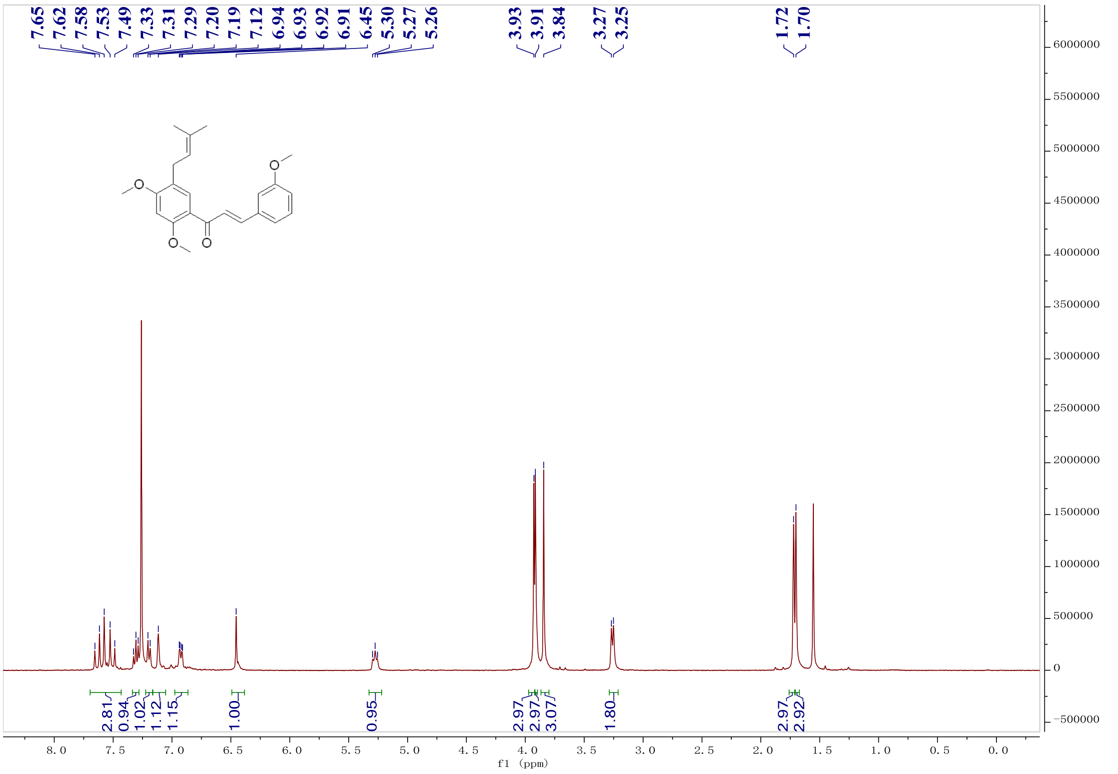

^{13}C NMR of compound **38d**

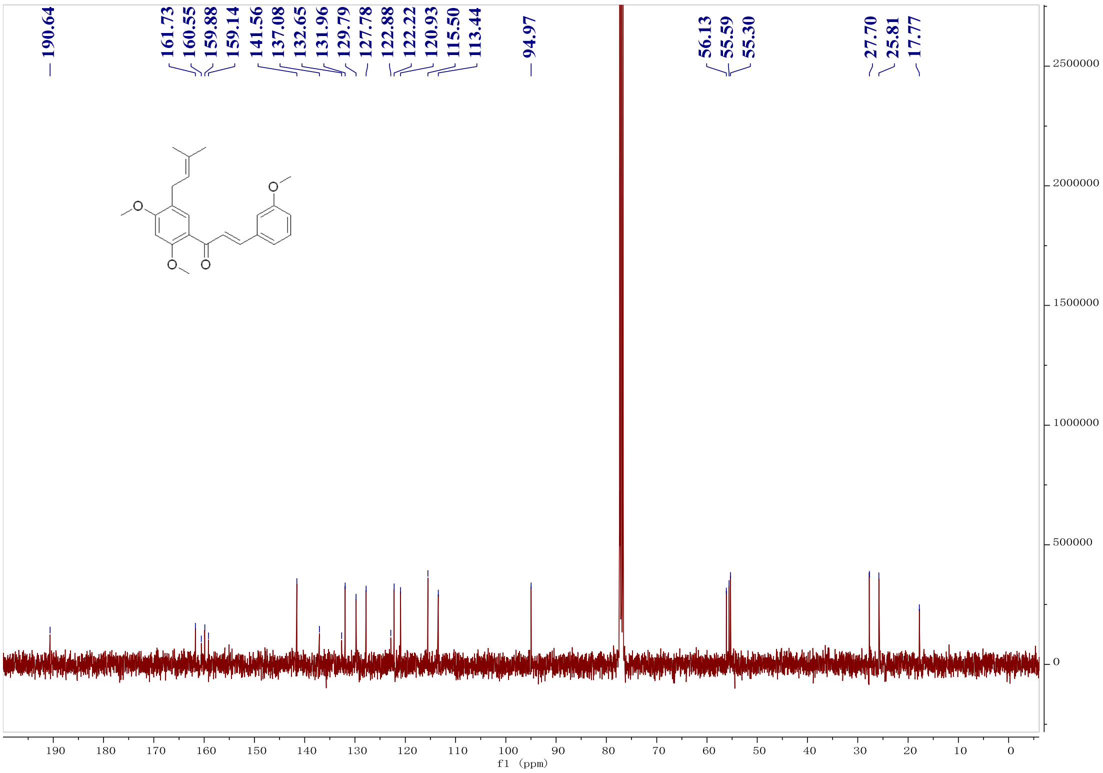

^{1}H NMR of compound **38e**

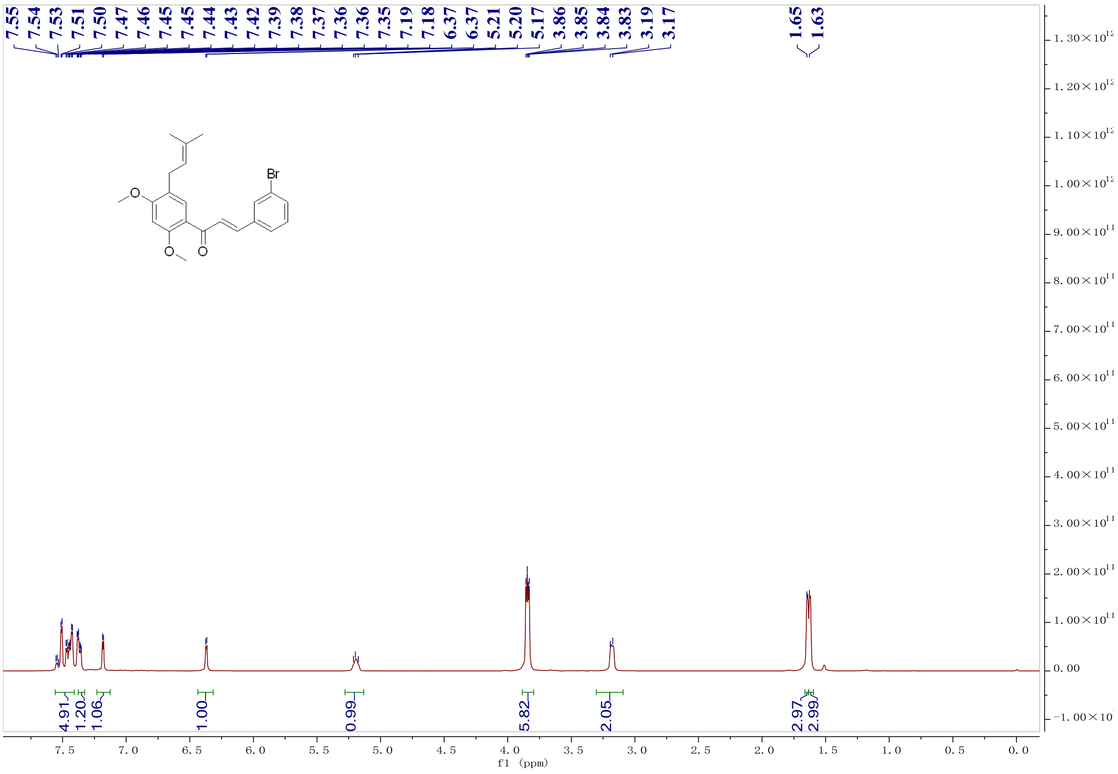

^{13}C NMR of compound **38e**

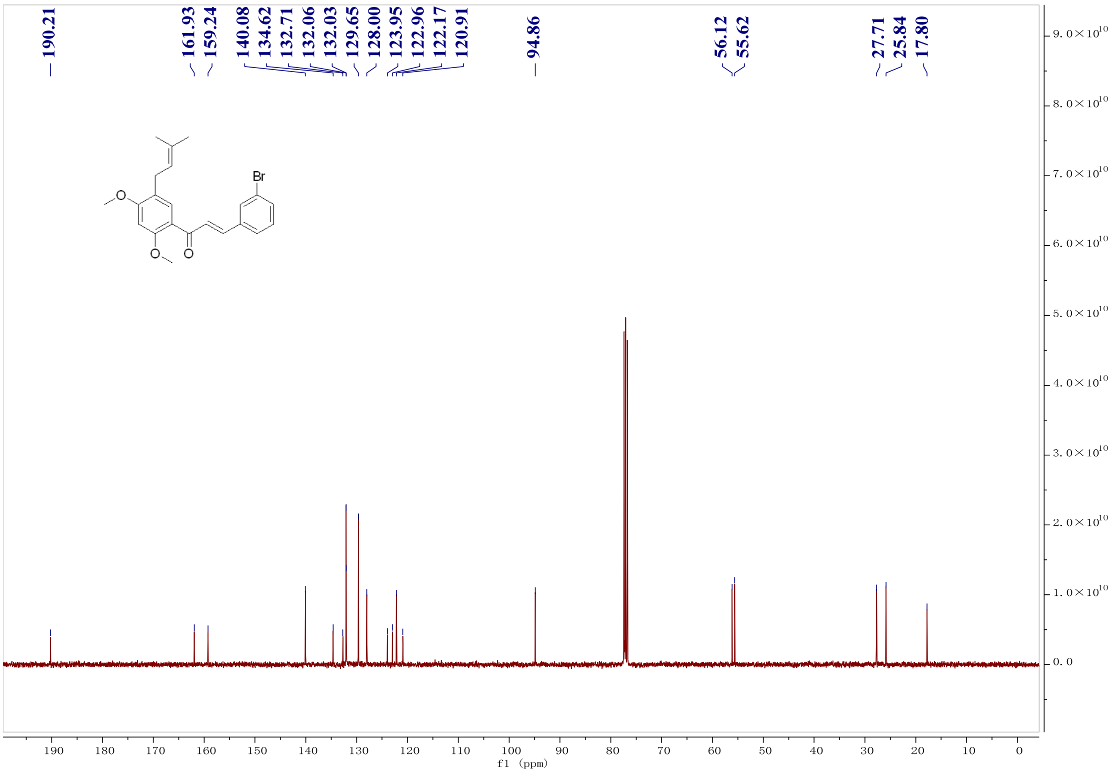

^{1}H NMR of compound **38f**

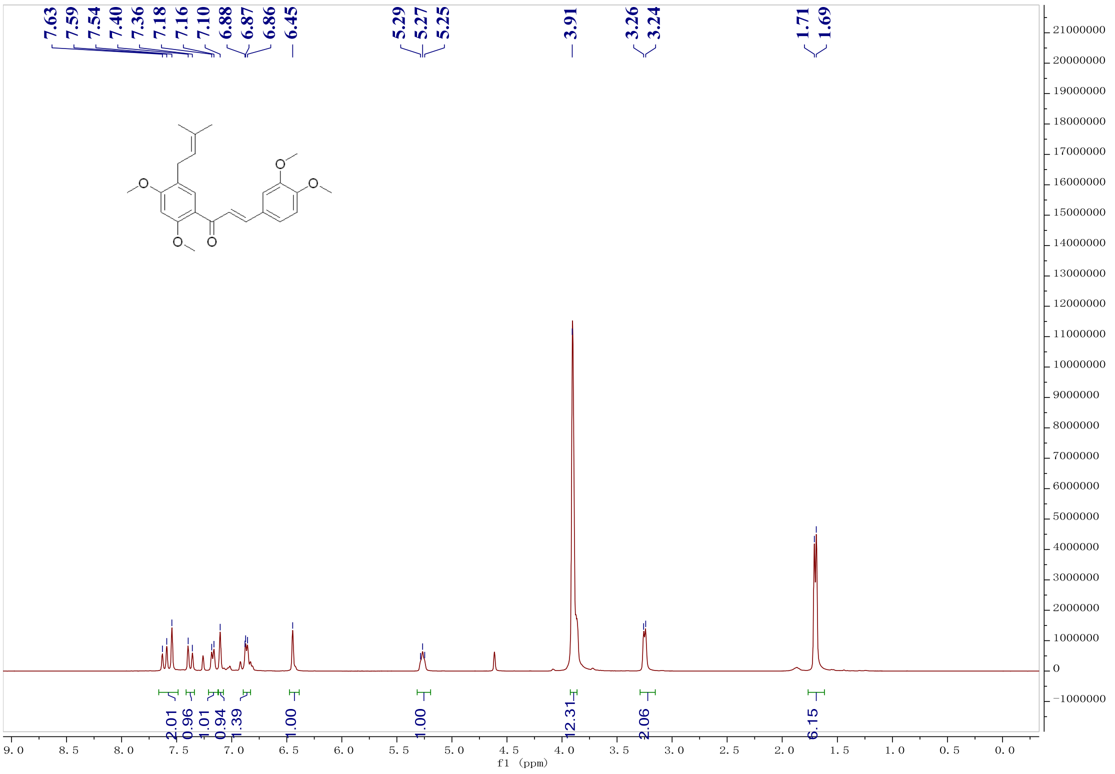

^{1}H NMR of compound **39a**

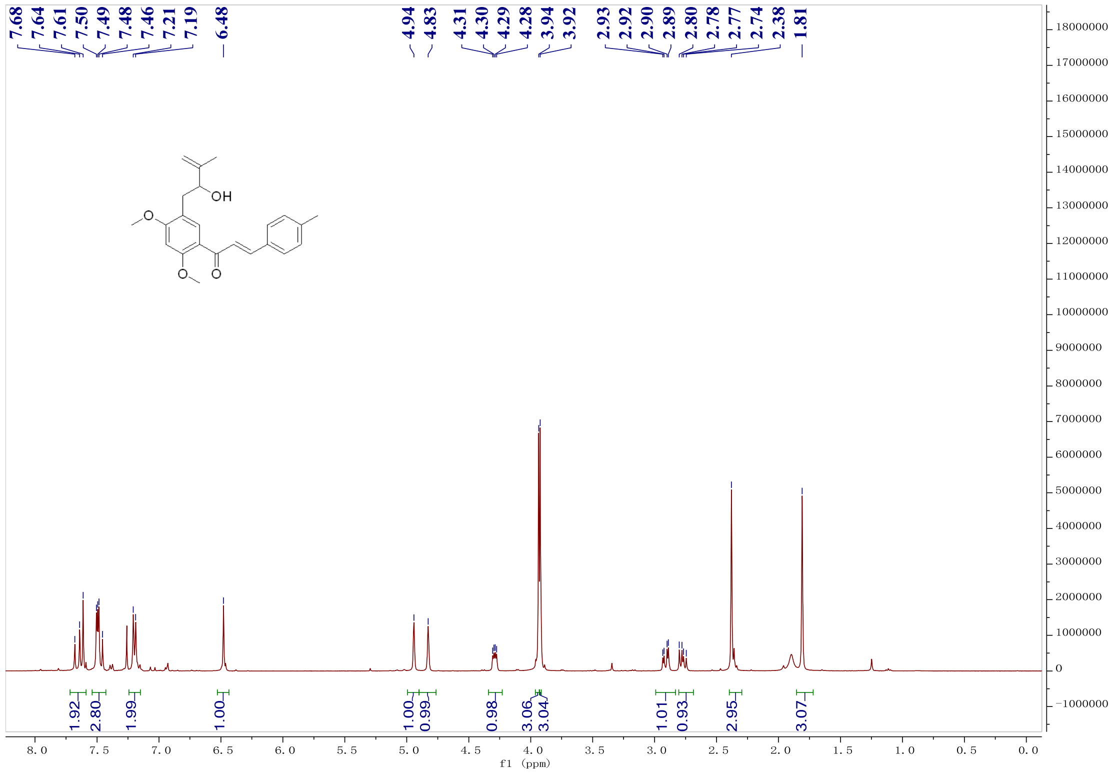

^{13}C NMR of compound **39a**

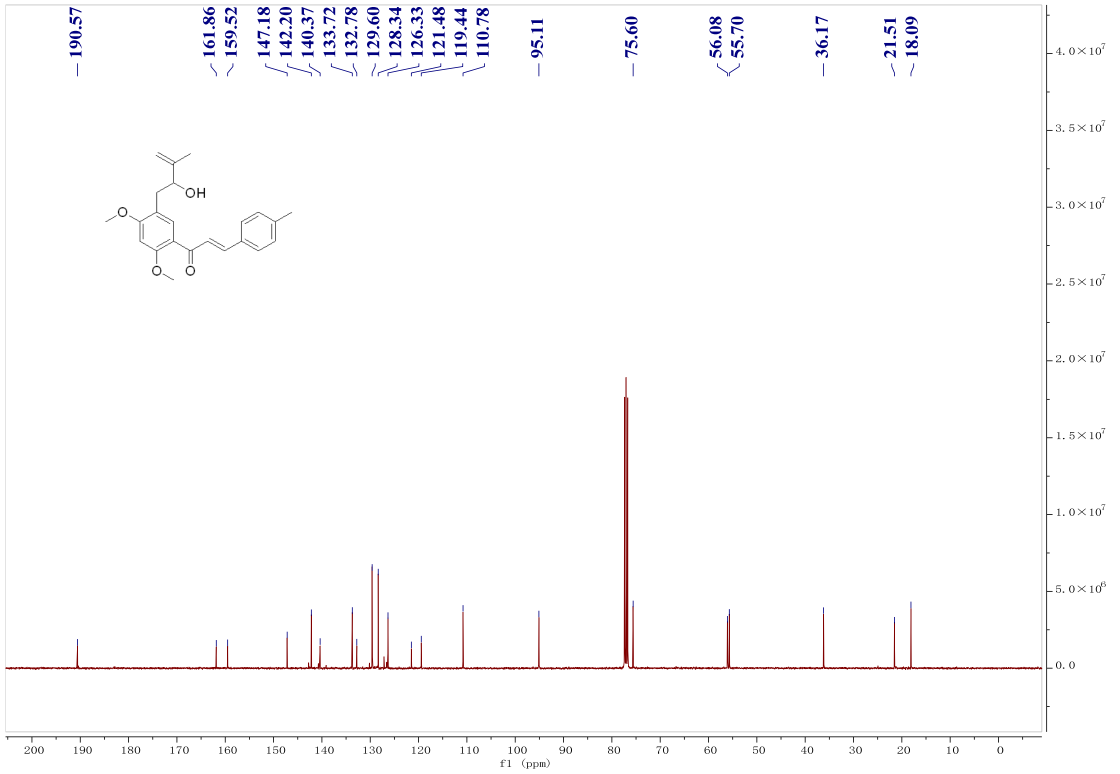

^{1}H NMR of compound **39b**

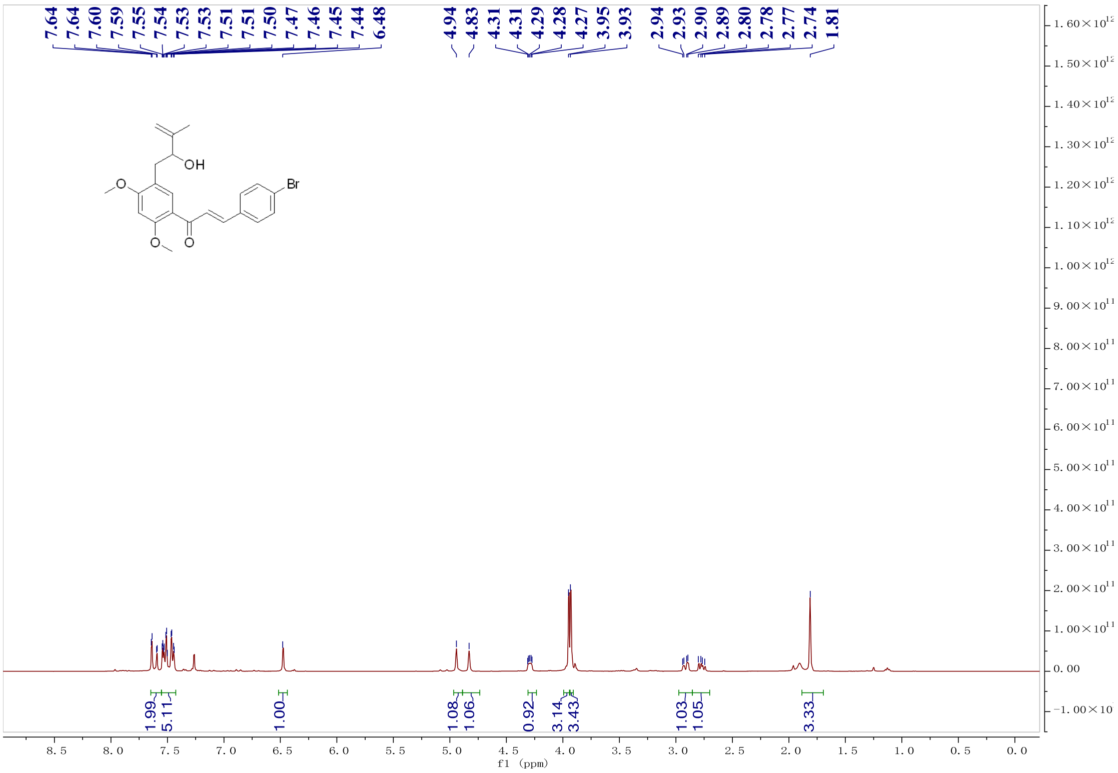

^{13}C NMR of compound **39b**

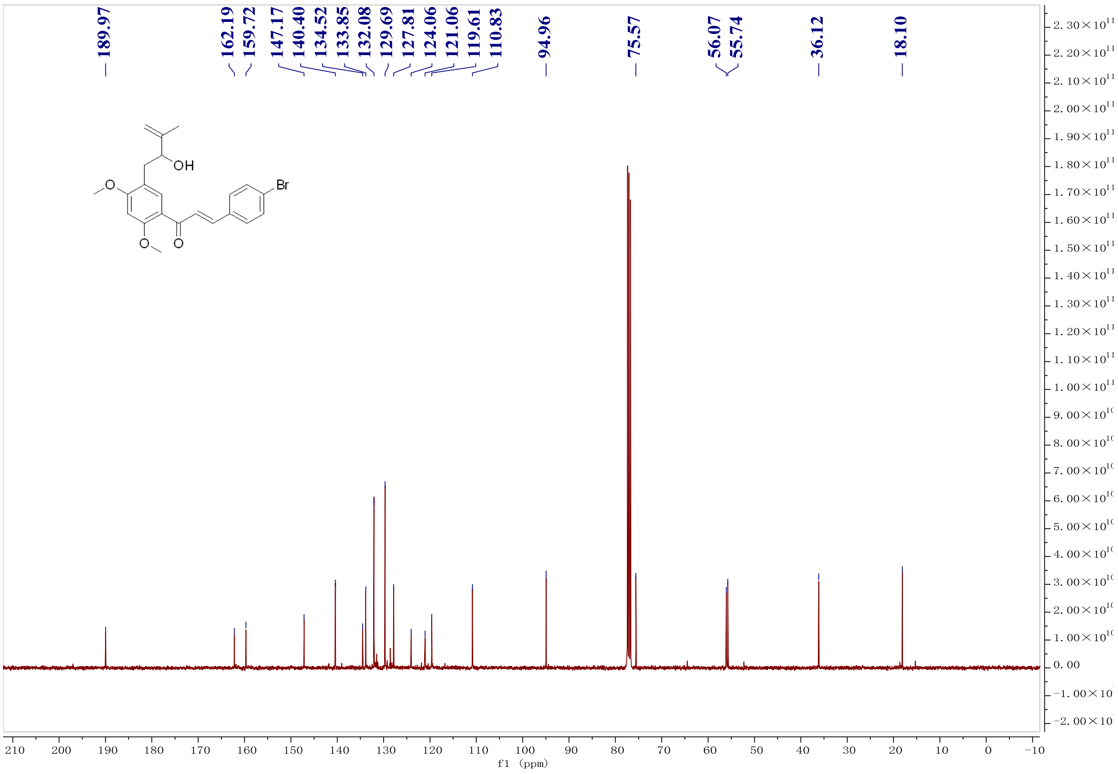

^{1}H NMR of compound **39c**

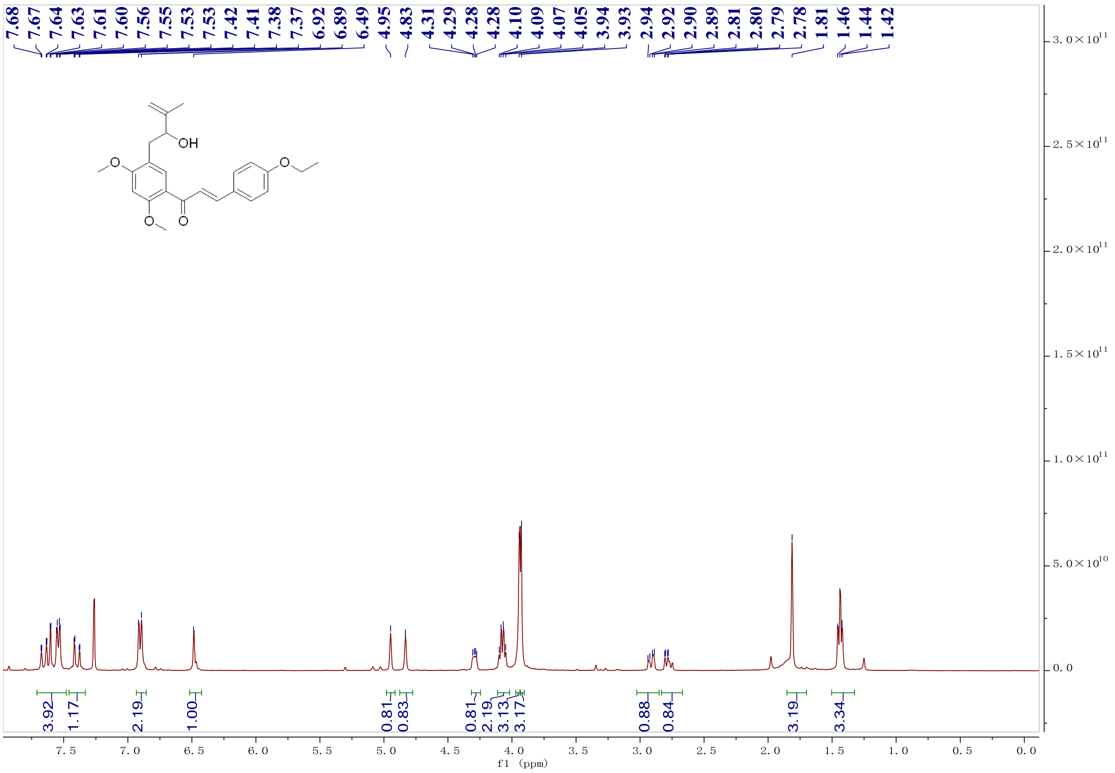

^{13}C NMR of compound **39c**

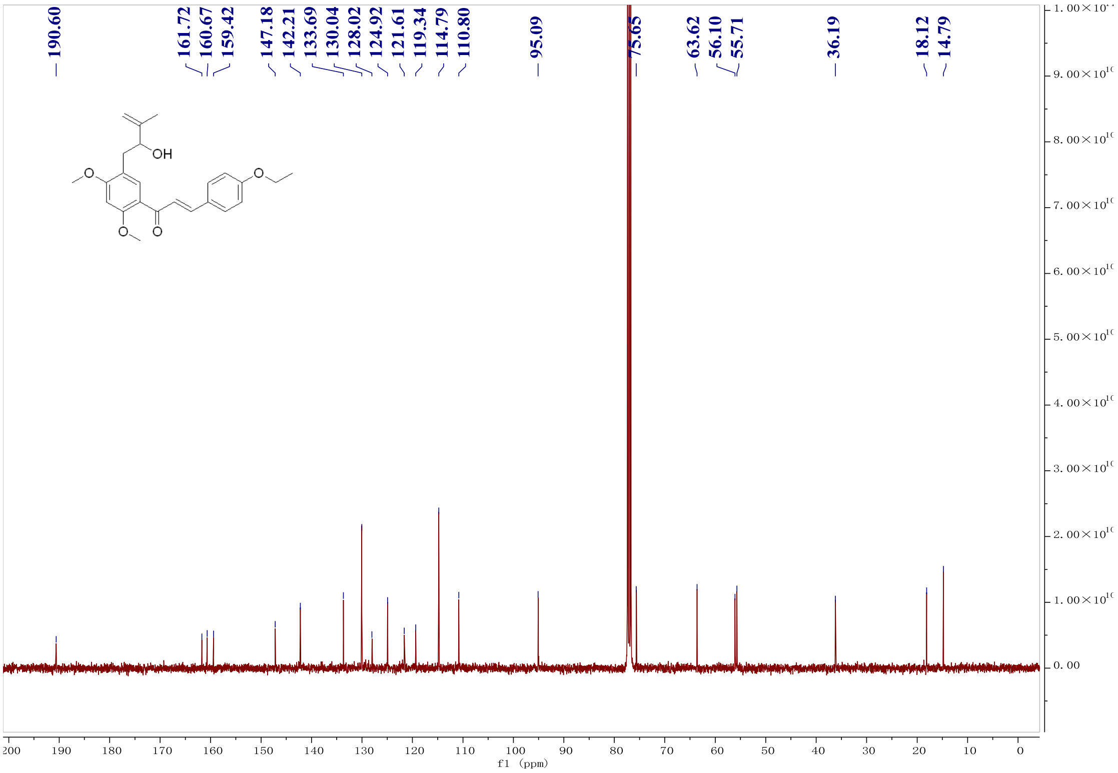

^{1}H NMR of compound **39d**

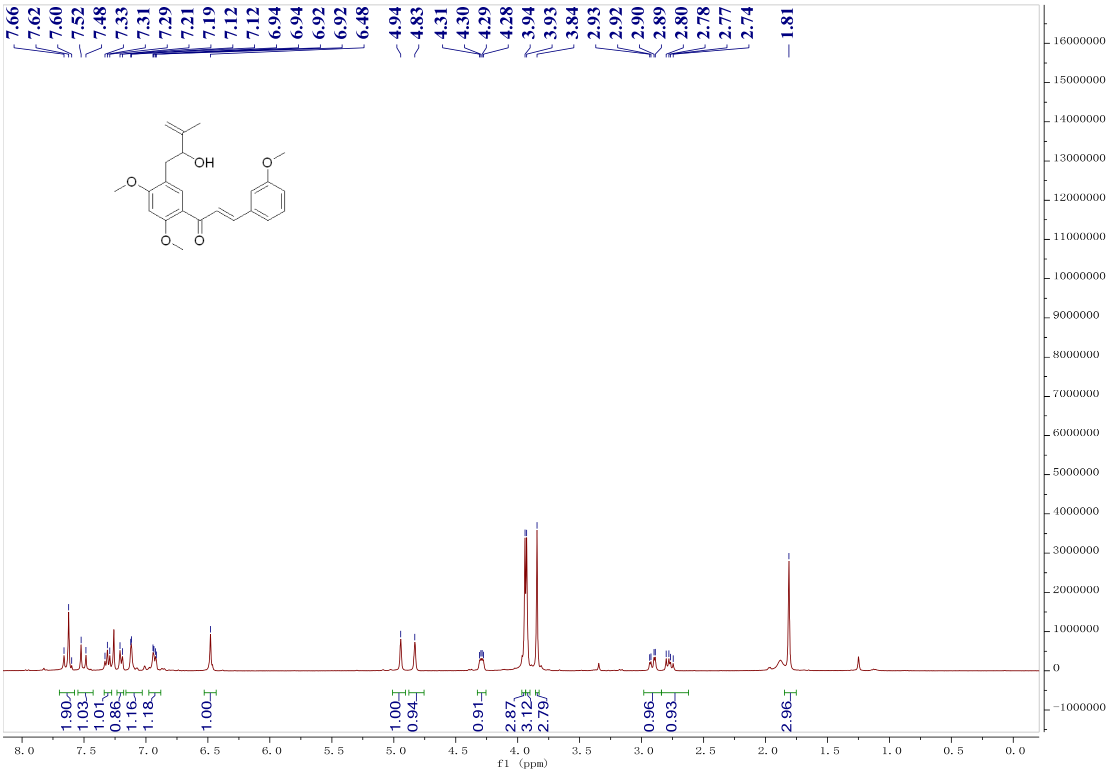

^{13}C NMR of compound **39d**

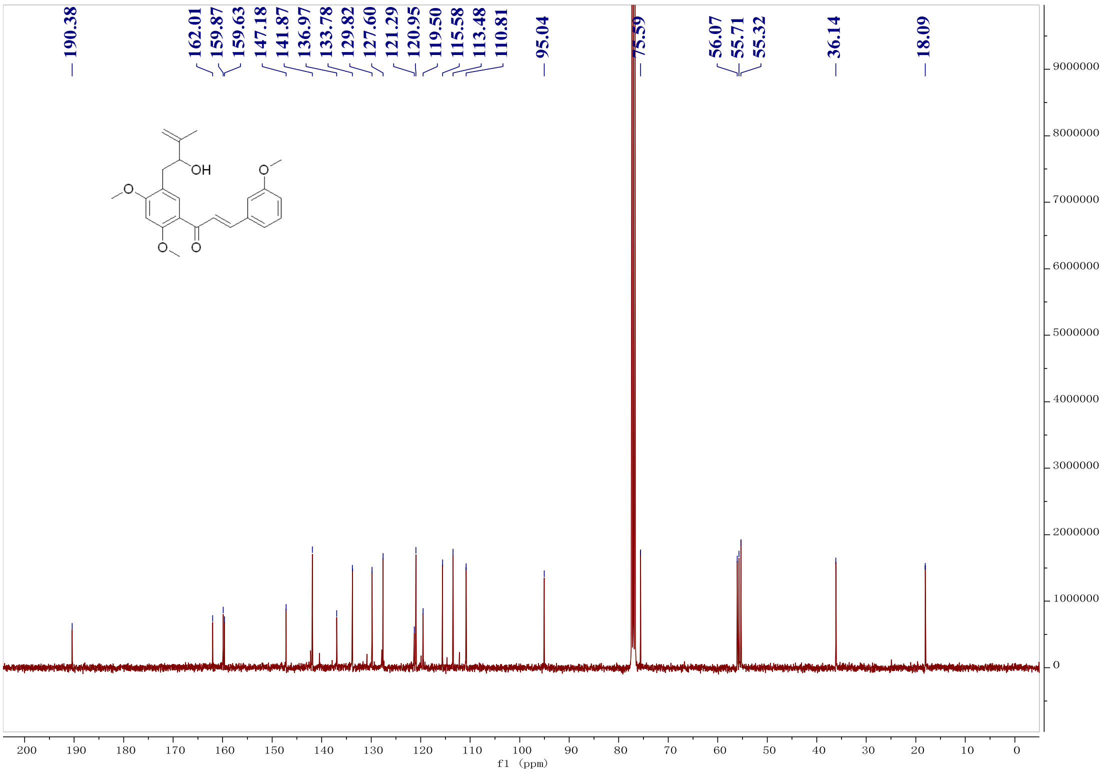

^{1}H NMR of compound **39e**

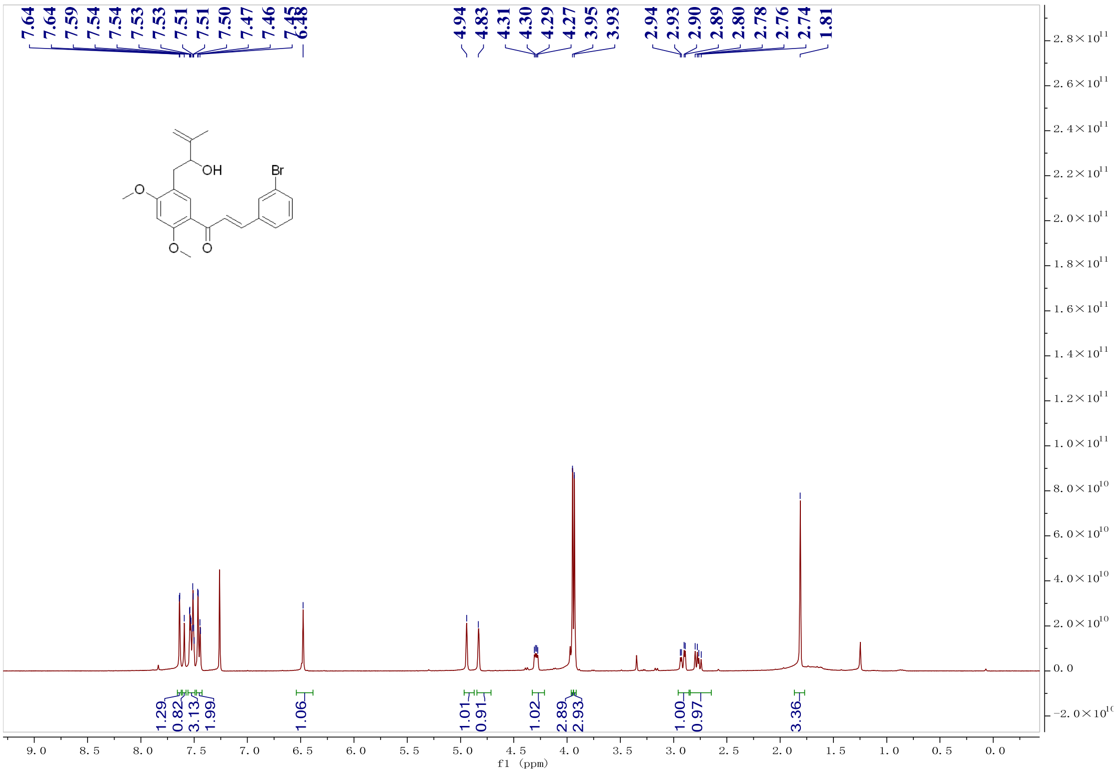

^{13}C NMR of compound **39e**

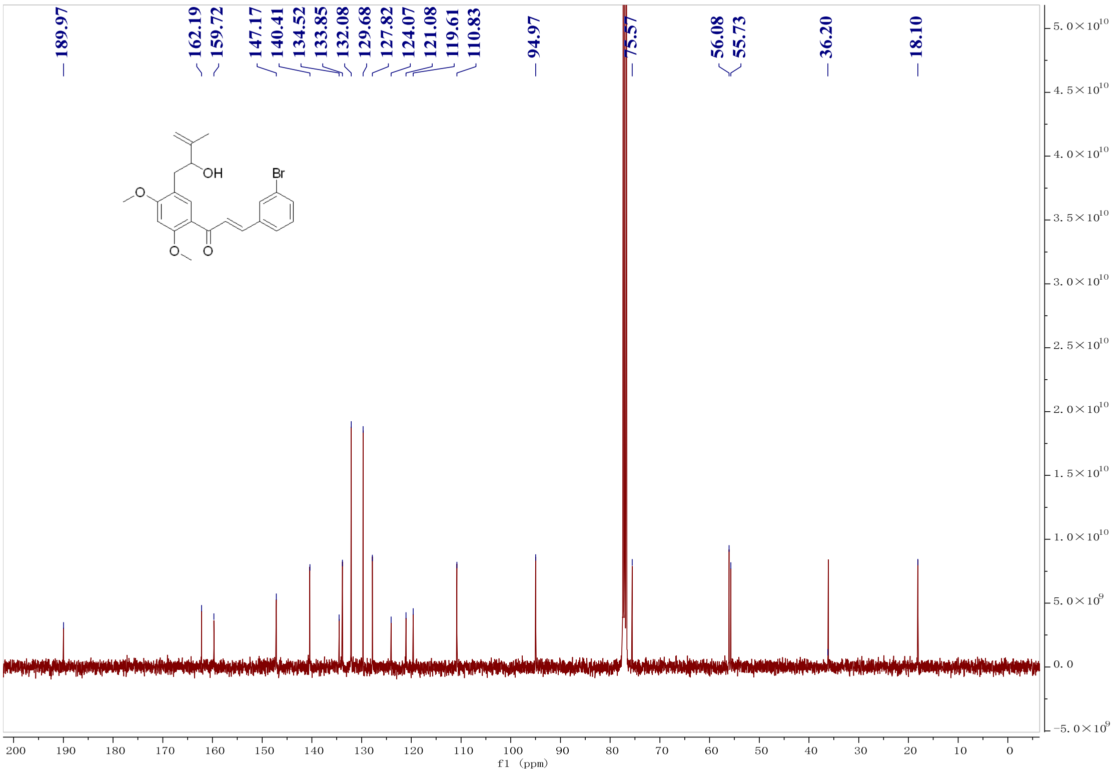

^{1}H NMR of compound **39f**

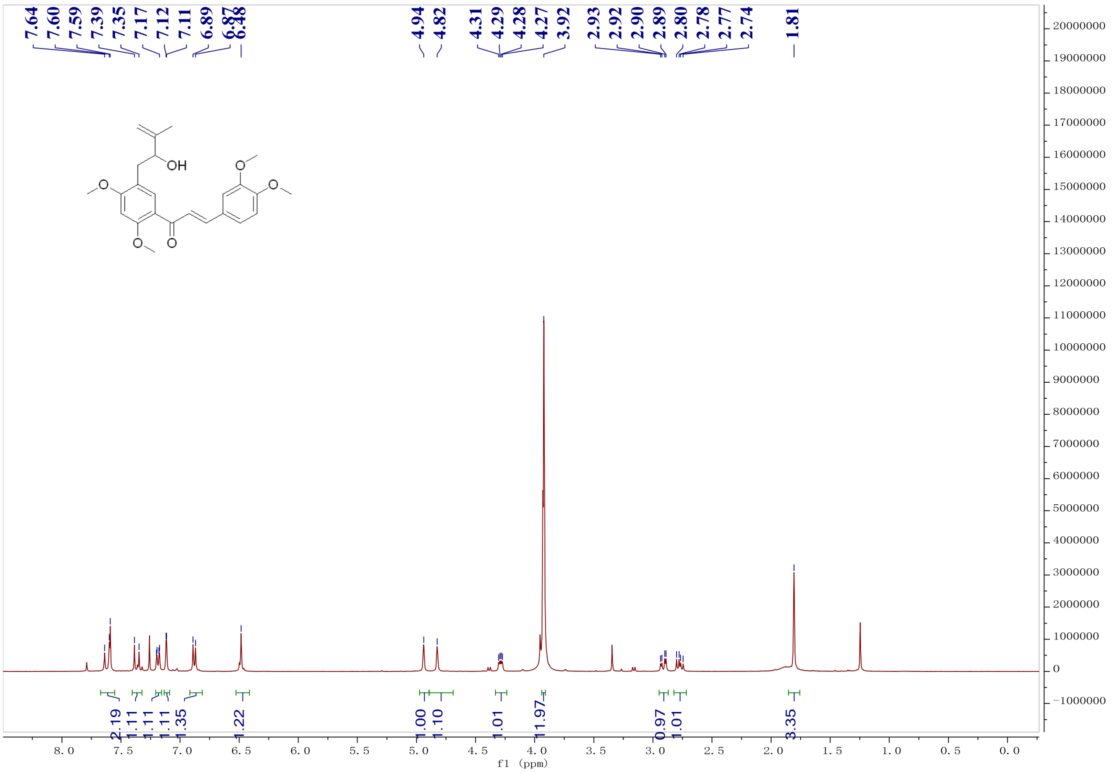

^{13}C NMR of compound **39f**

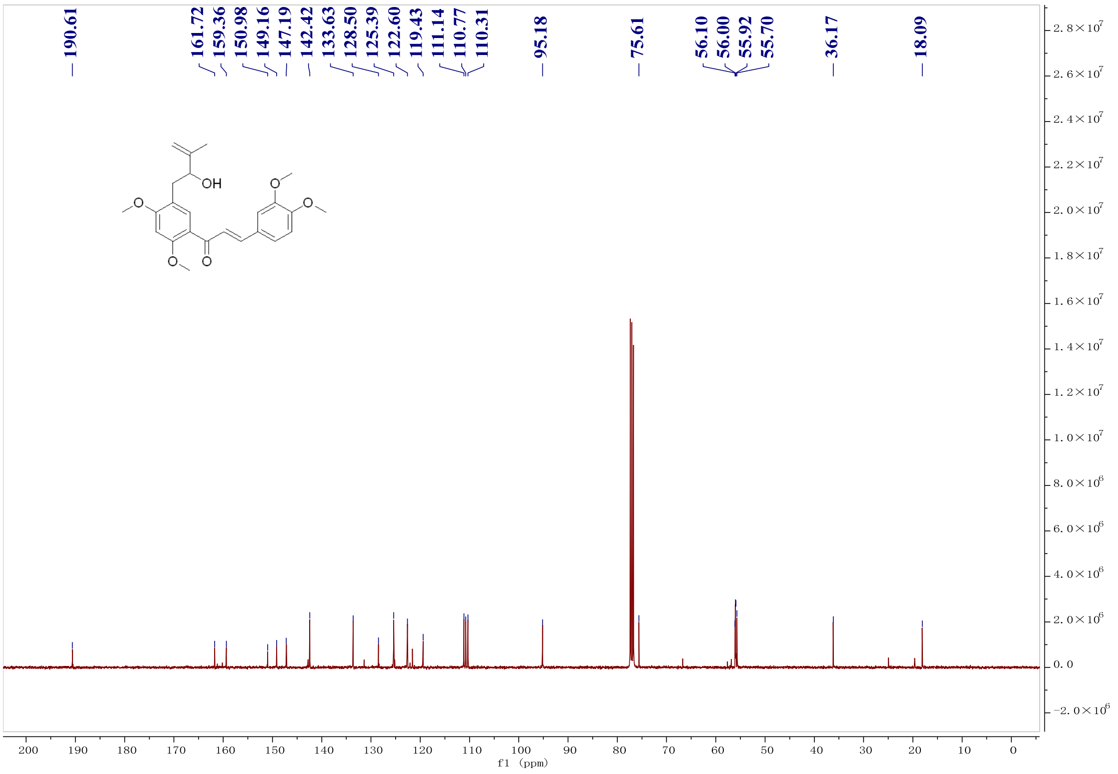

6. References

[1] Ribaudo, G.; Coghi, P.; Zanforlin, E.; Law, B. Y. K.; Wu, Y. Y. J.; Han, Y.; Qiu, A. C.; Qu, Y. Q.; Zagotto, G.; Wong, V. K. W., Semi-synthetic isoflavones as BACE-1 inhibitors against Alzheimer's disease. *Bioorganic Chemistry* **2019,** *87*, 474-483.

[2] Amolak C. J ;Bhola N. S.; Synthesis of Alpinum Isoflavone, Osajin, and Warangalone. *The Journal of Organic Chemistry* **1974**, 39 (15), 2215-2217.
[3] Bensinger, D.; Stubba, D.; Cremer, A.; Kohl, V.; Waßmer, T.; Stuckert, J.; Engemann, V.; Stegmaier, K.; Schmitz, K.; Schmidt, B., Virtual Screening Identifies Irreversible FMS-like Tyrosine Kinase 3 Inhibitors with Activity toward Resistance-Conferring Mutations. *Journal of Medicinal Chemistry* **2019,** *62* (5), 2428-2446.
[4] Dong, T.; Li, C.; Wang, X.; Dian, L.; Zhang, X.; Li, L.; Chen, S.; Cao, R.; Li, L.; Huang, N.; He, S.; Lei, X., Ainsliadimer A selectively inhibits IKKα/β by covalently binding a conserved cysteine. *Nature Communications* **2015,** *6* (1).
